# Sustainable Access to *N*‐Heterocycles: One‐Pot Cascade Synthesis of Pyrroles From Bio‐Derived Furans

**DOI:** 10.1002/anie.8203203

**Published:** 2026-09-08

**Authors:** Haifeng Qi, Germán López Robledo, Jianglin Duan, Yujing Ren, Kathrin Junge, Matthias Beller

**Affiliations:** ^1^ Leibniz‐Institut Für Katalyse Rostock Germany; ^2^ Interdisciplinary Research Center of Biology & Catalysis School of Life Science and Technology Northwestern Polytechnical University Xi'an China

**Keywords:** C─O cleavage, highly dispersed nanoparticles, Pt, selective hydrogenation, solid acid

## Abstract

The synthesis of pyrroles, fundamental nitrogen‐heterocyclic scaffolds in pharmaceuticals and materials science, remains a synthetic challenge, typically requiring pre‐functionalized precursors and multi‐step protocols. Here, we report an efficient cascade synthesis of *N*‐substituted pyrroles directly from bio‐derived furans and nitroarenes, enabled by a synergistic bifunctional catalyst. The catalyst, featuring highly dispersed platinum (Pt) clusters anchored on a Brønsted acid‐rich zeolite (HY), achieves near‐quantitative yields (up to 97%) under atmospheric H_2_ pressure. Systematic characterization, including aberration‐corrected transmission electron microscopy and x‐ray absorption spectroscopy, reveals that the Pt clusters provide the necessary moderate hydrogenation activity, thereby selectively reducing nitroarenes to anilines while leaving the furan ring intact. In contrast, Pt nanoparticles, owing to their excessive hydrogenation activity, lead to undesired over‐hydrogenation of both the furan and benzene rings. The zeolite's Brønsted acid sites subsequently facilitate a rapid ring‐opening/condensation sequence, enabling an oxygen‐to‐nitrogen transmutation to form pyrroles. This atom‐economic strategy tolerates a broad range of functional groups (>30 examples) and is further validated by the streamlined synthesis of a potent antimycobacterial drug candidate. Our findings demonstrate how modulating the electronic structure and ensemble configuration of noble metals at the atomic scale can unlock novel, sustainable transformation pathways for biomass‐derived platform chemicals.

## Graphical abstract

Highly dispersed platinum (Pt) clusters on Brønsted acid‐rich HY zeolite enable a one‐pot cascade conversion of bio‐derived furans and nitroarenes into pyrroles. Highly dispersed Pt sites ensure selective nitro hydrogenation, while Pt nanoparticles promote undesired over‐hydrogenation. The Pt–acid synergy drives ring opening and condensation, offering a sustainable route to diverse *N*‐heterocycles.

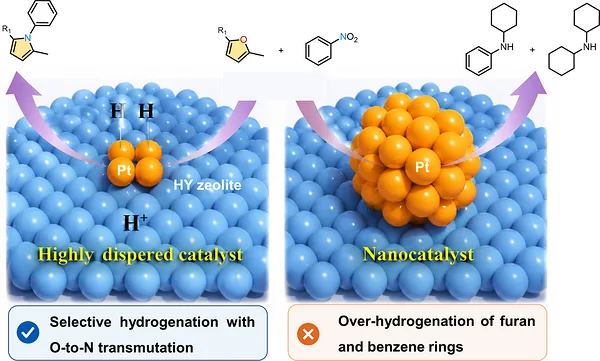

## 1 Introduction

Amines and nitrogen‐containing heterocycles constitute foundational motifs across chemical synthesis, materials science, and biological systems, with nitrogen atoms embedded in the vast majority of pharmaceuticals and functional molecules [1, 2, 3, 4, 5, 6]. Notably, nitrogen constitutes ∼3.0% of total human body mass, thereby underscoring the pivotal role of nitrogen‐bearing scaffolds in physiology and human health [7, 8]. Consistent with this, over 80% of the top 200 selling drugs in 2024 contain at least one nitrogen functionality [9], highlighting the importance of *N*‐based motifs in modern therapeutics. Among these, pyrroles represent a privileged class of heterocycles that are widely found in natural products, bioactive compounds, and advanced materials, where subtle structural variations can profoundly influence function (see selected examples in Figure 1a) [10, 11, 12]. Despite their importance, the synthesis of pyrroles remains largely constrained by classical condensation strategies, most notably the Paal–Knorr reaction, which relies on prefunctionalized 2,5‐dicarbonyl compounds and amines (Figure 1b) [13]. More recently, catalytic cascade approaches using diketones or dimethoxytetrahydrofuran in combination with nitroarenes have been developed (Figure 1b) [14, 15, 16, 17, 18]. All of these catalysts are characterized by the fact that they use formic acid or formic acid/base mixtures as reductants. There is one exception, however: a catalyst developed by us that works with molecular hydrogen and CO/H_2_O mixtures [16]. Moreover, in all reported procedures, whether they are catalytic or traditional synthetic methods, scarcely available and highly reactive yet unstable substrates such as diketones and dimethoxytetrahydrofuran are applied as starting materials, limiting their broader applicability. In parallel, an alternative approach pioneered by Kempe and colleagues, involving homogeneous or heterogeneous iridium‐catalyzed dehydrogenative coupling of secondary alcohols and amino alcohols, has emerged as an attractive alternative for the construction of pyrrole skeletons [19, 20].

**FIGURE 1:**
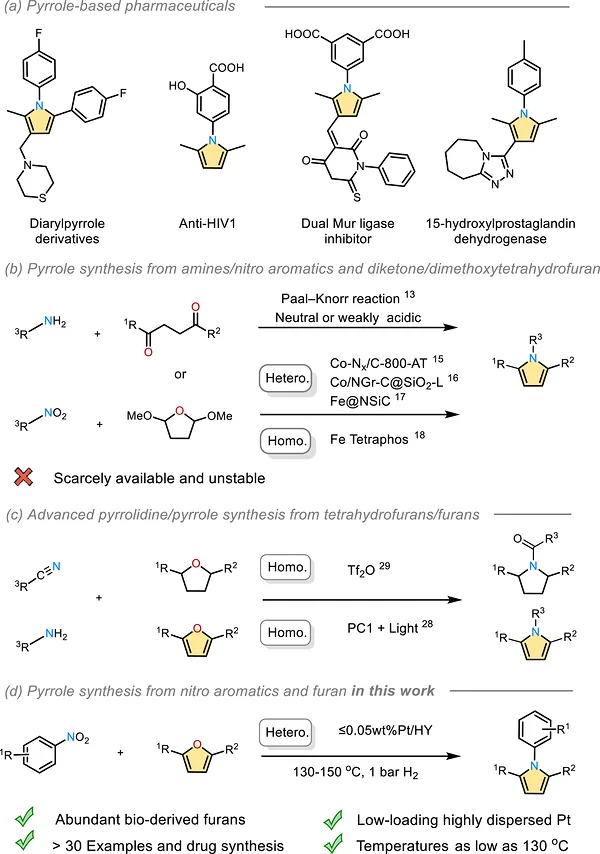
Past and present works of pyrrole synthesis. (a) Examples of pyrrole‐based pharmaceuticals. (b) Traditional pyrrole synthesis. (c) Catalytic heterogeneous and homogeneous systems for pyrrole synthesis from 2,5‐diketone/2,5‐dimethoxytetrahydrofuran and nitroarenes. (d) This work: pyrrole synthesis starting directly from furans and nitroarenes.

A more compelling strategy is to directly construct pyrroles from abundant feedstocks through cascade transformations that integrate multiple elementary steps within a single catalytic framework. In this context, bio‐derived furans represent particularly attractive starting materials, as they share the same five‐membered ring framework as pyrroles, differing only in the heteroatom (O vs. N) [21]. Furans are ubiquitous motifs in natural products and medicinal agents, and extensive methodologies have been developed for their peripheral C─H functionalization [22, 23, 24, 25]. Recent advances have demonstrated the feasibility of heterocycle interconversion from bio‐based platform molecules; for example, the transformation of furfural into six‐membered N‐heterocyclic piperidines has been achieved [26, 27]. In contrast, strategies enabling direct oxygen‐to‐nitrogen transmutation within the five‐membered ring framework remain largely underdeveloped. Notably, homogeneous catalytic systems have recently enabled oxygen‐to‐nitrogen single‐atom substitution in furan and tetrahydrofuran derivatives, affording pyrroles or pyrrolidines (Figure 1c) [28, 29]. However, these transformations generally require either molecular catalysts or activated furan substrates. The present catalytic system extends this strategy to simple alkyl‐substituted furans and nitroarenes, enabling their direct conversion into pyrroles through a heterogeneous cascade process without the need for activating functional groups. Achieving this goal is inherently challenging, as reported in previous studies [30, 31] and illustrated in Figure S1, it requires orchestrating multiple elementary steps within a single system, including nitroarene hydrogenation, selective furan ring opening to 2,5‐diketone intermediates, condensation with amines, and subsequent cyclization. Critically, the selective cleavage of the C─O bond in the furan ring under hydrogenation conditions represents a key bottleneck, demanding precise control over reaction pathways.

Here, we report a multifunctional catalytic system that enables the direct cascade synthesis of pyrroles from nitroarenes and furans in a single operation (Figure 1d). The catalyst, consisting of highly dispersed Pt species supported on a Brønsted acid‐rich zeolite, operates at ultra‐low metal loadings and mediates the sequential hydrogenation, ring‐opening, condensation, and cyclization steps with high efficiency. This atom‐economical transformation proceeds under relatively mild conditions and provides straightforward access to a broad range of structurally diverse N‐substituted pyrroles in good to excellent yields.

## 2 Results and Discussion

### 2.1 Catalyst Design and Structure–Selectivity Relationship

Initially, the catalytic system was designed to integrate selective nitroarene hydrogenation with furan activation and subsequent condensation–cyclization steps. Brønsted acid‐rich HY zeolite provides an effective platform for furan activation and C─O bond cleavage [31], while highly dispersed Pt species are uniquely capable of mediating chemoselective nitroarene hydrogenation [32, 33, 34]. Therefore, integrating these functionalities within a single catalyst enables a cooperative reaction network that is inaccessible to either component alone. On this basis, a series of Pt/HY catalysts were constructed (Figure 3a) and evaluated for their catalytic performance in the benchmark reaction involving 2,5‐dimethylfuran and nitrobenzene, which are known as representative biomass‐derived platform molecules and promising bulk chemicals, respectively [35, 36].

**FIGURE 2:**
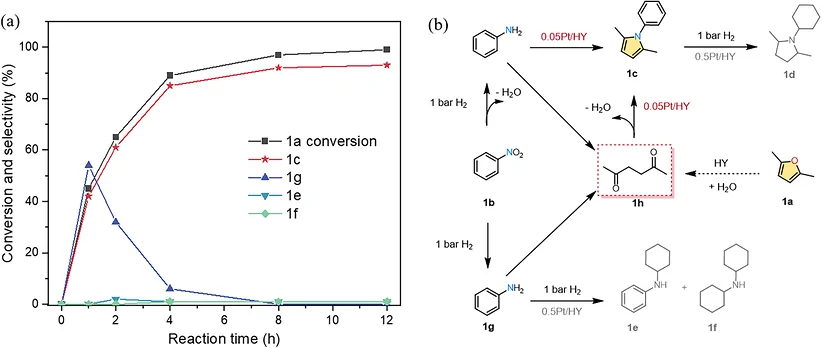
Kinetic profile and reaction mechanism. (a) Kinetic profile of the 0.05Pt/HY catalyst and (b) proposed catalyst–selectivity relationship. Reaction conditions: 0.25 mmol **1a**, 0.25 mmol **1b**, 100 mg catalyst, 1 mL toluene, 1 bar H_2_, 20 bar N_2_, 130°C, for a defined reaction time.

In order to achieve a high degree of selectivity in the hydrogenation of nitroarenes and to avoid the formation of undesired side‐products, the reaction was conducted at a low hydrogen pressure of 1 bar H_2_. As shown in Table 1, when the Pt loading is set at 1%, products **1e** and **1f** are mainly formed, resulting from hydrogenation of nitrobenzene and subsequent coupling, next to small amounts of the over‐hydrogenated product **1d**. This can possibly be explained by the high hydrogenation capability of Pt nanoparticles (Table 1, entry 1) [37]. Consequently, we systematically reduced the Pt loading to enhance its dispersion and modulate its hydrogenation activity. Notably, with the gradual decrease in Pt loading, the yield of the target product **1c** gradually increases, while the formation of products **1e** and **1f** is suppressed (Table 1, entries 2–7). At a Pt loading of 0.05%, exclusively pyrrole **1c** derived from nitrobenzene with dimethylfuran is obtained as the primary product (Table 1, entry 5)! We conclude that at a Pt loading as low as 0.05%, Pt is predominantly in a highly dispersed state, leading to reduced hydrogenation activity and thus preventing over‐hydrogenation. To prove this hypothesis, we further decreased the Pt loading to 0.01% and 0.001%, resulting in the production of the target product without significant over‐hydrogenation products. Considering the ultralow Pt loading in the ppm range, the attainment of a highly dispersed state is facilitated, resulting in inherently superior selective hydrogenation performance. From an economic perspective, the trace Pt metal usage enhances the feasibility and attractiveness of industrial processes. To validate the respective roles of Pt and HY in the reaction, several comparative experiments were conducted. In the absence of Pt metal in the catalyst, no hydrogenation products were observed, underscoring the indispensability of Pt species for nitrobenzene hydrogenation (Table 1, entry 8). Furthermore, when Pt was loaded onto alumina and silica, the yield of the target product notably decreased, with over‐hydrogenated coupling products from nitrobenzene emerging as the main products (Table 1, entries 9 and 10), indicating a synergistic role of Brønsted acid sites in promoting pyrrole formation. To substantiate this speculation, Pt‐loaded alumina and silica were physically mixed with HY support, resulting in an increased yield of the target product, indicative of HY's ability to catalyze the coupling of aniline and dimethylfuran (Table 1, entries 11 and 12). We further investigated 0.05Pt/HY‐30 catalysts with a higher SiO_2_/Al_2_O_3_ ratio of 30, which possess a lower Brønsted acid‐site density. Interestingly, pyrrole **1c** remained the major product with a yield of 89%, suggesting that the presence of Brønsted acid sites, rather than simply their density, is critical for the hydrolytic ring‐opening process. In contrast, 0.05Pt/NaY, which lacks Brønsted acid sites, gave only a low yield of pyrrole, while nitrobenzene hydrogenation products and their coupling products became dominant (Table S1). These results highlight the essential role of Brønsted acid sites in promoting the ring‐opening of the furan ring.

**TABLE 1:** Catalytic condensation of 2,5‐dimethylfuran with nitrobenzene over various catalysts^a^.

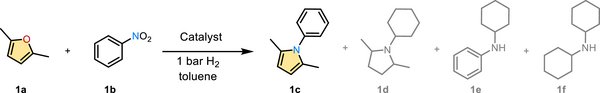					
Entry	Catalysts^b^	Yield (%)^d^			
		**1c**	**1d**	**1e**	**1f**
1	1Pt/HY	n.d.	9	63	20
2	0.5Pt/HY	15	11	37	25
3	0.2Pt/HY	23	2	21	32
4	0.1Pt/HY	81	n.d.	2	14
5	0.05Pt/HY	93	n.d.	1	1
6	0.01Pt/HY	94	n.d.	1	1
7	0.001Pt/HY	97	n.d.	n.d.	n.d.
8	HY	n.d.	n.d.	n.d.	n.d.
9	0.05Pt/Al_2_O_3_	21	n.d.	69	n.d.
10	0.05Pt/SiO_2_	33	n.d.	54	n.d.
11^c^	0.05Pt/Al_2_O_3_ + HY	67	n.d.	24	6
12^c^	0.5Pt/Al_2_O_3_ + HY	25	21	36	15

^a^ Reaction conditions: 0.25 mmol **1a**, 0.25 mmol **1b**, 100 mg catalyst, 1 mL toluene, 1 bar H_2_, 20 bar N_2_ (introduced to suppress solvent evaporation), 130°C, 12 h.

^b^ Pt‐loading corresponds to its theoretical value.

^c^ 100 mg catalyst + 100 mg HY.

^d^ GC yield; n.d.: not detected.

To investigate the reaction pathway for the formation of pyrrole from 2,5‐dimethylfuran **1a** and nitroarene **1b**, a kinetic profile was done (Figures 2 and S2). As seen in Figure 2a, there is no apparent induction period needed for the catalysis. After 1 h, the nitroarene was fully hydrogenated to yield aniline. Then, after 4 h, the pyrrole yield increased to more than 80%. No other intermediate could be detected over the entire course of the reaction for the 0.05Pt/HY catalyst. In contrast, undesired hydrogenation of both the furan and benzene rings occurs over the 0.5Pt/HY catalyst (Figure S2). Further validation experiments were conducted to investigate the individual elementary steps involved in the cascade process. HY zeolite was found to catalyze the coupling of aniline and 2,5‐dimethylfuran to afford the desired pyrrole product at reaction temperatures above 130°C (Figure S3), consistent with literature reports where acid can catalyze a similar process [38]. Considering that 2,5‐hexanedione **1h** would be a potential intermediate from ring‐opening of **1a** based on previous reports [39], it was used directly as a substrate in the reaction with aniline in the presence of HY zeolite (Figure S4). As expected, a 98% yield of pyrrole **1c** was obtained at a reaction temperature above 120°C. In addition, 0.05Pt/HY and 0.5Pt/HY were applied to compare their performance in the hydrogenation of nitrobenzene. It was observed that 0.05Pt/HY selectively formed aniline, while in the case of 0.5Pt/HY, over‐hydrogenation of aniline occurred, leading to coupling and partial hydrogenation of the aromatic ring (Figure S5). Notably, nitroarene hydrogenation readily occurred at temperatures as low as 40°C. These results indicate that hydrogen activation and nitroarene hydrogenation are kinetically facile under the reaction conditions and are therefore unlikely to be rate‐limiting. Given that the transformation involving 2,5‐dimethylfuran requires the highest reaction temperature, we infer that the hydrolytic ring‐opening of 2,5‐dimethylfuran to form the reactive 2,5‐hexanedione intermediate is the most energy‐demanding step in the overall cascade pathway. Based on these control experiments, a reaction mechanism was proposed (Figure 2b). The selective hydrogenation of nitroarenes **1b** to aniline initially happened on 0.05Pt/HY, while 0.5Pt/HY exhibited strong hydrogenation ability and led to coupling and over‐hydrogenation of aniline. During the reaction, water is generated in situ from both nitroarene hydrogenation and the subsequent condensation of **1h** with aniline. According to previous studies [28, 40], the ring‐opening of **1a** proceeds via a Brønsted acid‐catalyzed hydrolytic pathway involving water. Therefore, the water formed in situ can facilitate the hydrolysis of **1a** into the proposed intermediate **1h** over HY. The resulting **1h** then rapidly undergoes condensation with aniline to produce pyrrole **1c**. This condensation step is expected to proceed rapidly, consistent with the absence of detectable 2,5‐hexanedione in the kinetic profiles (Figure 2a), again suggesting that the hydrolytic ring‐opening of 2,5‐dimethylfuran is likely the most energy‐demanding step in the overall cascade process.

### 2.2 Structural Characterization and Their Correlation With Catalytic Performance

To elucidate the origin of the distinct catalytic performance of the Pt/HY catalysts, the structural evolution of Pt species with varying loadings was first examined by x‐ray diffraction (XRD). As shown in Figure 3b and Figure S6, no diffraction peaks corresponding to Pt species are detected at loadings below 0.5 wt%, indicating a high dispersion of Pt. Upon increasing the loading to 0.5 wt%, a weak diffraction peak at 39.7° corresponding to Pt (111) emerges, which becomes more pronounced at 1 wt%, evidencing the formation of Pt nanoparticles. This structural evolution correlates well with the observed catalytic selectivity. Specifically, at loadings ≥ 0.5 wt%, Pt predominantly exists as nanoparticles with strong hydrogenation capability, leading to over‐hydrogenation. In contrast, at lower loadings, Pt species are highly dispersed, favoring selective hydrogenation of nitro groups. Based on these distinct catalytic behaviors, 0.5Pt/HY and 0.05Pt/HY were selected for detailed structural analysis. Although catalysts with lower Pt loadings (e.g., 0.01Pt/HY and 0.001Pt/HY) also exhibited excellent catalytic performance, their Pt contents were insufficient to provide reliable signals for advanced characterization techniques such as x‐ray absorption spectra (XAS) and aberration‐corrected high‐angle annular dark‐field scanning transmission electron microscopy (HAADF‐STEM).

**FIGURE 3:**
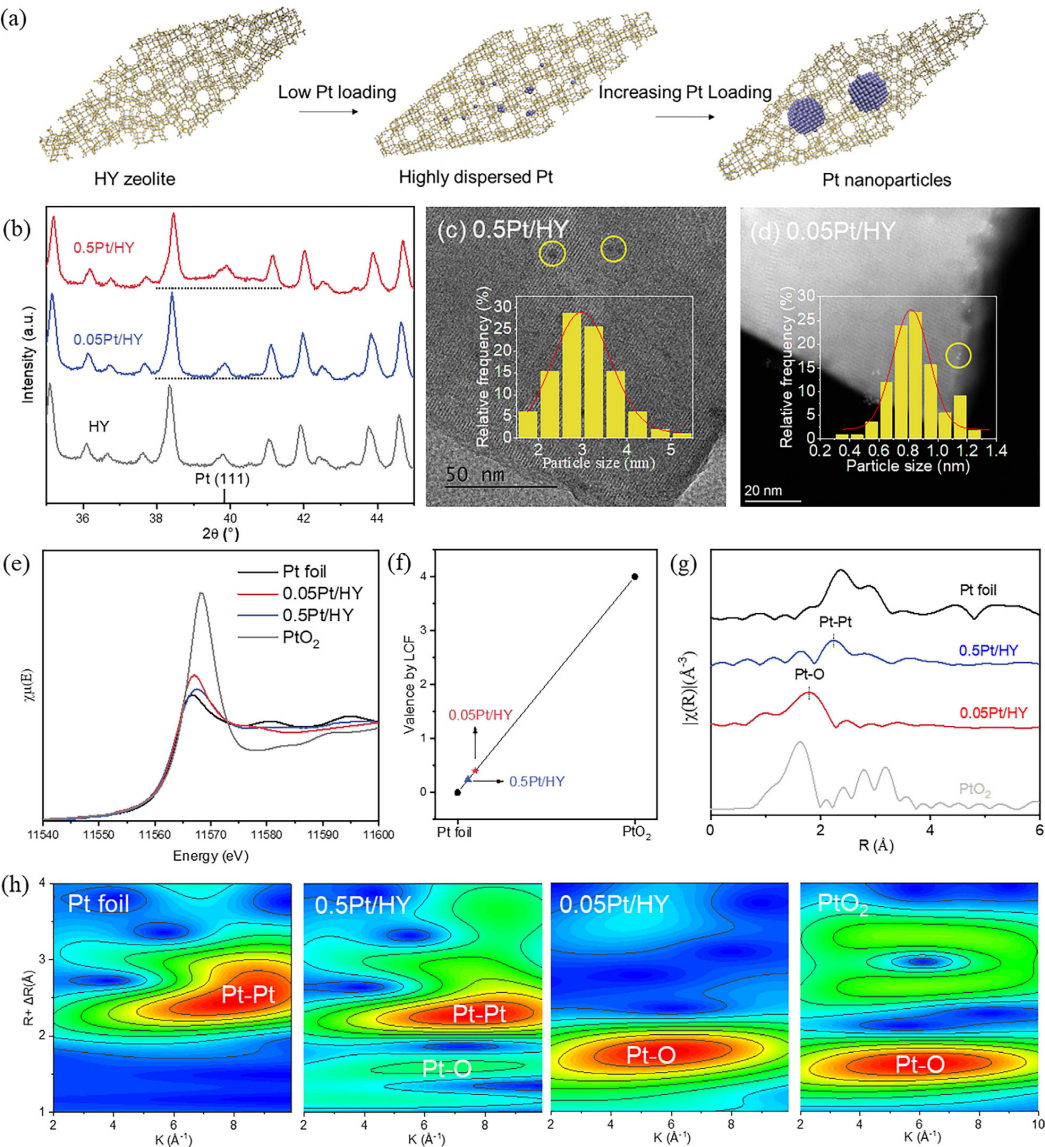
Characterization of Pt/HY catalysts. (a) Synthetic process of Pt/HY catalysts, (b) XRD patterns, Spherical aberration‐corrected transmission electron microscopy images of (c) 0.5Pt/HY and (d) 0.05Pt/HY, (e) the normalized XANES spectra at the Pt L_III_‐edge, (f) linear combination fitting of XANES, (g) the *k^2^*‐weighted Fourier transform of EXAFS spectra in *r*‐space, (h) wavelet transform of the *k^2^*‐weighted EXAFS signal of 0.5Pt/HY, 0.05Pt/HY and references.

Figures 3c,d and S7 display aberration‐corrected HAADF‐STEM images of the 0.5Pt/HY and 0.05Pt/HY catalysts. For the 0.5Pt/HY sample, the average size of Pt nanoparticles is 3 nm, suggesting the formation of a Pt–Pt ensemble structure. In sharp contrast, small ∼0.8 nm clusters can be observed in 0.05Pt/HY with weak Pt signals. According to the relationship between Pt particle size and dispersion ($D=6*\left(v_{m}/a_{m}\right)\approx 1.29/d_{s}$, *D* is the dispersion, *a*_m_ (Å^2^) is the surface area of an atom, *v*_m_ (Å^3^) is the volume of an atom, and *d* (nm) is the particle diameter) [41], such ultrasmall Pt clusters are expected to exhibit exceptionally high dispersion. Although this empirical relationship becomes less rigorous in the sub‐nanometer regime, the average particle size of ∼0.8 nm indicates that the vast majority of Pt atoms are located at or near the cluster surface. Therefore, 0.05Pt/HY can be reasonably described as a highly dispersed Pt cluster catalyst with an exceptionally high fraction of surface‐exposed Pt atoms.

XAS at the Pt L_III_‐edge was performed to provide further evidence about the oxidation states and the local coordination of Pt atoms [42]. In the Pt L_III_‐edge x‐ray absorption near‐edge structure (XANES) spectra (Figure 3e), the white‐line intensity of 0.05Pt/HY is slightly higher than that of 0.5Pt/HY but lower than that of PtO_2_, indicating partially oxidized Pt species with a positive charge state in 0.05Pt/HY, in agreement with x‐ray photoelectron spectroscopy results (Figure S8). To further quantify the relative contributions of different Pt species, linear combination fitting (LCF) analysis of the XANES spectra was performed (Figures 3f and S9, and Table S2). The experimental spectrum of both catalysts can be reasonably reproduced using Pt foil and PtO_2_ as reference compounds, indicating that Pt in 0.05Pt/HY possesses a more positive average electronic state than that in 0.5Pt/HY. Consistently, the white‐line intensity of 0.5Pt/HY is close to that of Pt foil, suggesting Pt nanoparticles are most likely formed in the 0.5Pt/HY sample. Further insights were obtained from extended x‐ray absorption fine structure (EXAFS) analysis (Figure 3g); the *k* ^2^‐weighted Fourier transform of the EXAFS in *r*‐space of the 0.5Pt/HY displays a prominent peak at 2.26 Å (not phase‐corrected), which could be ascribed to Pt–Pt coordination, confirming the presence of Pt nanoparticles. In contrast, 0.05Pt/HY displays a pattern quite different from that of 0.5Pt/HY, with a prominent peak at 1.78 Å and a weak feature at 2.44 Å (both not phase‐corrected), corresponding to Pt–O and minor Pt–Pt coordination, respectively.

To further distinguish Pt–O and Pt–Pt coordination, wavelet transform (WT) analysis of the Pt L_III_‐edge EXAFS oscillations was performed, as WT provides enhanced resolution in both *k* and *r* spaces (Figure 3h) [43]. A pronounced lobe at (2.5 and 8.2 Å^−1^), characteristic of Pt–Pt contributions, is clearly visible in the WT contour plots of both the Pt foil standard and the 0.5Pt/HY sample, confirming the dominance of Pt–Pt coordination in the 0.5Pt/HY sample. In contrast, the WT lobe of the 0.05Pt/HY sample shifts to lower *r* and *k* values (1.8 and 5.5 Å^−1^), indicative of predominant Pt–O coordination. Consistently, the best‐fit EXAFS analysis of 0.05Pt/HY yields coordination numbers (CN) of 3.4 for Pt–O and 1.1 for Pt–Pt (Figure S10a,b and Table S3), suggesting the presence of highly dispersed Pt clusters.

By comparison, the 0.5Pt/HY sample exhibits spectra resembling those of Pt foil in both *k*‐ and *r*‐space, with fitting results showing a Pt–O CN of 0.5 and a Pt–Pt CN of 5.6 (Figure S10c,d and Table S3), consistent with the formation of Pt nanoparticles in the STEM result.

Based on the above structural differences (Figure 3) and distinct catalytic performances (Table 1), it can be inferred that ultrasmall Pt clusters with partial positive charge are proposed to possess moderate hydrogenation activity, which could selectively hydrogenate nitro groups while leaving the furan ring intact. In contrast, metallic Pt nanoparticles exhibit much stronger hydrogenation activity under the same conditions, leading to non‐selective over‐hydrogenation of not only the furan ring but also the benzene ring, thereby reducing the reaction selectivity.

### 2.3 Substrate Scope, Functional Group Tolerance, and Synthetic Utility

Nitrogen‐containing pyrroles represent privileged scaffolds in modern pharmaceuticals, functional materials, and bioactive molecules [44]. Accordingly, the development of general and robust strategies for their construction from readily available feedstocks remains highly desirable. To evaluate the scope and generality of the present catalytic system, a diverse set of nitroarenes was examined under standard conditions without further optimization (Figure 4). For substrate scope evaluation, 0.01Pt/HY was selected as the standard catalyst because it provided excellent activity and selectivity while maintaining an ultralow Pt loading. In addition, a slight excess of furan (1.2 equiv.) and a prolonged reaction time (24 h) were employed to ensure complete conversion of nitroarene substrates and reliable product isolation across substrates with diverse electronic and steric properties. A wide range of alkyl‐substituted nitrobenzenes and nitronaphthalene derivatives smoothly underwent the transformation, affording the corresponding N‐substituted pyrroles in good to excellent yields (**1c**–**4c**). Notably, the reaction exhibits broad functional‐group tolerance, accommodating synthetically versatile substituents such as halides, carbonyl groups, and methoxy moieties (**5c**–**13c**). These functionalities provide opportunities for subsequent derivatization via cross‐coupling, nucleophilic substitution, or carbonyl transformations, thereby enabling further molecular diversification.

**FIGURE 4:**
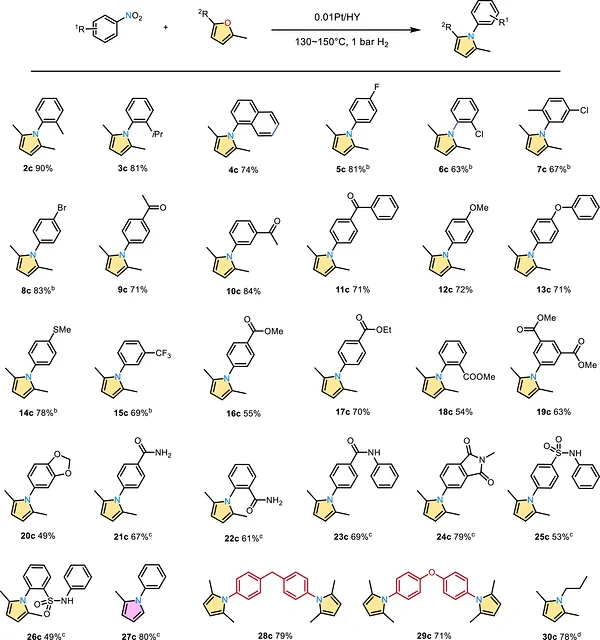
Substrate scope: condensation of different furans with nitrobenzene bearing various functional groups. ^a^Reaction conditions: 0.3 mmol **1a** (furans), 0.25 mmol **1b** (nitroarenes), 100 mg 0.01Pt/HY (0.02 mol% Pt relative to nitroarene), 1 mL toluene, 1 bar H_2_, 20 bar N_2_, 130°C, 24 h; ^b^140°C; ^c^150°C; ^d^GC yield; isolated yield is based on nitroarenes. NMR spectra of the products can be found in Figures S17–S48.

More challenging substrates bearing electron‐rich or strongly electron‐withdrawing groups, including methylthio, trifluoromethyl, ether, and ester substituents, were also compatible, delivering the desired pyrrole products in moderate to good yields (**14c**–**20c**). The tolerance of such groups is particularly noteworthy, as they often interfere with catalytic hydrogenation processes through coordination, electronic perturbation, or competitive reduction pathways. These results highlight the chemoselectivity of the present system toward nitro group reduction while preserving other sensitive functionalities.

Primary amides, which are ubiquitous in amino acids, pharmaceuticals, and biologically active molecules, are traditionally challenging substrates in transition‐metal‐catalyzed reactions due to their protic nature and strong coordination ability. Encouragingly, the present catalytic system tolerates primary amides well, furnishing the corresponding products in good yields (**21c**–**26c**). This feature significantly expands the applicability of the methodology to more complex and biologically relevant molecules.

The versatility of this protocol is further demonstrated by its applicability to more demanding substrates. For instance, challenging substrates such as 2‐methylfuran (**27c**), sterically hindered dinitro‐substituted aromatics (**28c**–**29c**), and even aliphatic nitroalkanes, exemplified by 1‐nitropropane (**30c**), which are generally less explored in such cascade transformations, can be effectively converted under the present mild reaction conditions. This highlights the efficiency and broad applicability of the catalytic system in enabling multi‐step cascade transformations.

Beyond substrate scope, the structural diversity of the obtained pyrroles underscores their synthetic value. The products incorporate a wide range of functional groups (e.g., halides, esters, amides, and heterocycles), rendering them versatile intermediates for downstream functionalization. Moreover, the construction of extended π‐conjugated systems (red‐highlighted examples) suggests potential applications in organic electronics, fluorescence materials, and charge‐transport systems [45, 46]. The ability to directly access such structurally diverse pyrroles from simple nitroarenes and furans provides a powerful platform for rapid molecular diversification and late‐stage functionalization.

### 2.4 Late‐stage Functionalization of Bioactive Molecules and Synthetic Applications

To further highlight the applicability of this tandem catalytic system toward structurally complex and functionally sensitive molecules relevant to life sciences, late‐stage reductive coupling of bioactive compounds was investigated (Figure 5a). Notably, nimesulide (**31b**), nimodipine (**32b**), and cilnidipine (**33b**) underwent smooth transformation with 2,5‐dimethylfuran (**1a**), affording the corresponding pyrrole derivatives in good yields (61%–72%). These results highlight the compatibility of the catalytic system with pharmaceutically relevant scaffolds, providing a practical platform for the rapid generation of pyrrole‐based analogues. It is noteworthy that partial hydrogenation of the pyrrole ring was observed in the case of **33c**, reflecting the delicate balance between activity and chemoselectivity in such complex molecular settings.

**FIGURE 5:**
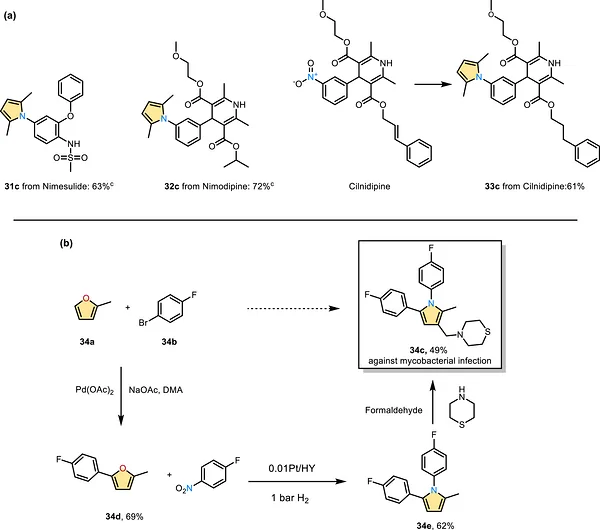
Modification and synthesis of bioactive molecules. (a) Condensation of complex molecules with 2,5‐dimethylfuran^a^; (b) bottom‐up synthesis of pharmaceutical derivatives^b^. ^a^Reaction conditions: 0.5 mmol **1a** (furans), 0.25 mmol **1b** (nitroarenes), 100 mg 0.01Pt/HY (0.02 mol% Pt), 1 mL toluene, 1 bar H_2_, 20 bar N_2_, 150°C, 24 h. ^b^Reaction conditions: step 1: 0.1 mg Pd(OAc)_2_, 1 mmol 2‐methylfuran **34a**, 1 mmol 1‐bromo‐4‐fluorobenzene **34b**, 1 mmol NaOAc, 10 mL DMA, 150°C, 24 h; step 2: obtained **34d** 2‐(4‐fluorophenyl)‐5‐methylfuran, 1 equiv. 1‐fluoro‐4‐nitrobenzene, 100 mg 0.01Pt/HY, 1 mL toluene, 140°C, 24 h; step 3: obtained **34e** 1,2‐bis‐(4‐fluoro‐phenyl)‐5‐methyl‐1H‐pyrrole, 1 equiv. thiomorpholine, 1 equiv. formaldehyde (40% in water), 5 mL acetic acid, room temperature, 6 h.

Beyond reactivity, catalyst stability and reusability are critical for practical applications. As shown in Figure S11, the highly dispersed Pt/HY catalyst can be readily recycled for up to three cycles without significant loss of activity. Although deactivation occurs upon further reuse, catalytic performance can be partially restored via a simple proton‐exchange treatment, underscoring the robustness and regenerability of the system. More importantly, the recovery of catalytic activity after restoration of the Brønsted acid sites further highlights their important role in the reaction, particularly in the acid‐catalyzed furan ring‐opening process.

To highlight this atom‐efficient tandem pyrrole synthetic method, we focused on the synthesis of a target compound of significant practical importance, such as 4‐[1,5‐bis‐(4‐fluoro‐phenyl)‐2‐methyl‐1H‐pyrrol‐3‐ylmethyl]‐thiomorpholine **34c**, which is an important drug for the treatment of mycobacterial infection (Figure 1a) [47]. In the first step, 5‐methylfuran **34a** reacted with 1‐bromo‐4‐fluorobenzene **34b** to form the coupling product 2‐(4‐fluorophenyl)‐5‐methylfuran **34d** in 69% yield (Figure 5b, NMR, Figure S12). In the following step, this substituted furan (**34d**) was reacted with 1‐fluoro‐4‐nitrobenzene, applying our pyrrole synthetic method to yield 62% of 1,2‐bis‐(4‐fluorophenyl)‐5‐methyl‐1H‐pyrrole **34e** (Figure 5b, NMR, Figure S13), which is superior to previous synthetic approaches that rely on less readily available 2,5‐dione‐based substrate (Figure S16) [48]. Finally, the reaction with formaldehyde and thiomorpholine at room temperature produced the target product **34c** (Figure 5b, NMR, Figures S14 and S15), which is an effective drug candidate against mycobacterial infection related to BM 212 [49].

## 3 Conclusion

In summary, we have developed a bifunctional catalytic system based on highly dispersed Pt clusters anchored on a Brønsted acid‐rich HY zeolite, enabling the direct cascade synthesis of highly functionalized pyrroles from bio‐derived furans and nitroarenes in a one‐pot process. Mechanistic investigations reveal a clear catalytic synergy: the ultrasmall Pt clusters with partially positive electronic states mediate the selective hydrogenation of nitroarenes to anilines under mild conditions (1 bar H_2_), while the Brønsted acid sites promote furan ring opening and subsequent condensation. Crucially, the absence of extended Pt–Pt ensembles suppresses undesired over‐hydrogenation, in contrast to conventional Pt nanoparticles. The method features broad substrate scope (> 30 examples), high tolerance toward sensitive functional groups, and compatibility with complex bioactive molecules, enabling late‐stage functionalization and rapid molecular diversification. Furthermore, the ppm‐level noble metal loading, utilization of renewable furan feedstocks, and elimination of prefunctionalized intermediates collectively highlight the sustainability and practical relevance of this approach. This work establishes highly dispersed metal catalysis as a powerful platform for tandem transformations and provides a general strategy for the efficient synthesis of *N*‐heterocycles. We anticipate that this concept will be broadly applicable to other cascade reactions and valuable organic transformations.

## Author Contributions

**Haifeng Qi**: investigation, writing – original draft, methodology, formal analysis, conceptualization. **Germán López Robledo**: investigation, validation. **Jianglin Duan**: investigation, validation, methodology, writing – review and editing. **Yujing Ren**: investigation, methodology, visualization, writing – review and editing. **Kathrin Junge**: conceptualization, supervision, writing – review and editing. **Matthias Beller**: conceptualization, supervision, writing – review and editing, funding acquisition.

## Conflicts of Interest

The authors declare no conflicts of interest.

## Supporting information




**Supporting File**: anie74606‐sup‐0001‐SuppMat.pdf.

## Data Availability

The data that support the findings of this study are available in the Supporting Information of this article
