# Supplementary material for: Sustainable Access to N‐Heterocycles: One‐Pot Cascade Synthesis of Pyrroles From Bio‐Derived Furans
Source: Angew Chem Int Ed Engl. 2026 Sep 8;65(44):e8203203. doi: 10.1002/anie.8203203 (PMC13703903; doi:10.1002/anie.8203203)
Supplement: Supplementary file 1 [file ANIE-65-e8203203-s001.pdf]

Sustainable Access to N-Heterocycles: One-Pot Cascade Synthesis of Pyrroles from Bio-derived Furans

Haifeng Qi,[a] Germán López Robledo,[a] Jianglin Duan,[a,b] Yujing Ren,[b] Kathrin Junge,[a],* Matthias Beller [a],*

[a] Dr. H. Qi, G. López Robledo, J. Duan, Prof. K. Junge,* Prof. M. Beller,*
Leibniz-Institut für Katalyse
Albert-Einstein-Straße 29a, Rostock 18059, Germany
E-mail: kathrin.junge@catalysis.de, matthias.beller@catalysis.de

[b] J. Duan, Prof. Y. Ren
Interdisciplinary Research Center of Biology & Catalysis, School of Life Science and Technology
Northwestern Polytechnical University
Xi'an 710072, China

Inventory of Supporting Information

Methods..3

Supplementary Figure 1..5

Supplementary Figure 2..6

Supplementary Figure 3..7

Supplementary Figure 4..8

Supplementary Figure 5..9

Supplementary Figure 6..10

Supplementary Figure 7..11

Supplementary Figure 8..12

Supplementary Figure 9..13

Supplementary Figure 10..14

Supplementary Figure 11..15

Supplementary Figure 12..16

Supplementary Figure 13..17

Supplementary Figure 14..18

Supplementary Figure 15..19

Supplementary Figure 16..20-21

Supplementary Table 1..22

Supplementary Table 2..23

Supplementary Table 3..24

Supplementary References..25

NMR results..26-59

Methods

Materials. Platinum(0) 1,3-diethenyl-1,1,3,3-tetramethyldisiloxane, 2,5-dimethyl furan, nitrobenzene, and toluene were purchased from Sigma-Aldrich. Zeolite Y, hydrogen (SiO_2/Al_2O_3 = 5.1, 30) and Zeolite Y, sodium (SiO_2/Al_2O_3 = 5.1) were purchased from Alfa Aesar. All the other chemical reagents were used as received without further purification.

Catalyst preparation. All catalysts were prepared by incipient wetness impregnation. As an example, for the preparation of 0.01Pt/HY catalyst, 42 mg Platinum (0) 1,3-diethenyl-1,1,3,3-tetramethyldisiloxane were added to 1.5 mL toluene and sonicated for 10 min, followed by addition of 1 g Zeolite Y, hydrogen (SiO_2/Al_2O_3 = 5.1). Then the mixture was dried in an oven at 80 °C for 12 h. The obtained solid was ground to powder and then transferred to the tube furnace and then heated to 400 °C in air atmosphere at a ramp of 5 °C /min and was maintained at this temperature for 2 h. After cooling to room temperature, the gas was switched to hydrogen atmosphere and then heated to 200 °C in hydrogen atmosphere at a ramp of 5 °C /min and was held at 200 °C for 2 h. After being cooled to room temperature, the obtained sample was defined as 0.01Pt/HY catalyst, where 0.01 indicates a Pt loading of 0.01 wt%.

Catalytic reaction tests. In the typical reaction, 0.25 mmol 2,5-dimethylfuran, 0.25 mmol nitrobenzene, 100 mg 0.01Pt/HY catalyst, and 1 mL toluene were put into an 8 mL vial fitted with magnetic stirring bar and septum cap. A needle was inserted in the septum which allows gaseous reagents to enter. The vials (up to seven) were set in an alloy plate and then placed into a 300 mL steel Parr autoclave. After sealing the autoclave, the autoclave was purged with N_2 for three times and H_2 for three times and charged with 1 bar H_2 and 20 bar N_2 at room temperature, where the N_2 pressure was applied to suppress solvent evaporation during the reaction. Then the reaction mixture was stirred at a rate of 500 rpm and heated at 130 °C for 12 h. After the reaction, the crude media were dried by anhydrous Na_2SO_4. Then the isolated product was obtained by column purification using pentane/EtOAc mixture as eluent and submitted to NMR analysis.

The conversion of nitrobenzene ($X_{nitrobenzene}$) and the yield of pyrrole ($Y_{pyrrole}$) were determined according to the following equations:

$$\mathbf{X_{nitrobenzene}}\ (\%) = \frac{\mathbf{mol_{nitrobenzene\ consumed}}}{\mathbf{mol_{nitrobenzene\ fed}}} \times \mathbf{100} \qquad (1)$$

$$\mathbf{Y_{pyrrole}}\ (\%) = \frac{\mathbf{mol_{Pyrrole\ produced}}}{\mathbf{mol_{nitrobenzene\ fed}}} \times \mathbf{100} \qquad (2)$$

X-ray diffraction (XRD) analysis was carried out on a PANalytical X'pert diffractometer using Cu Kα radiation source (λ = 0.15432 nm) with a scanning angle (2θ) of 10°- 80°, operated at 40 kV and 40 mA.

The **aberration-corrected high-angle annular dark-field scanning transmission electron microscopy (HAADF-STEM)** experiments were performed on a JEOL JEM-2100F microscope operated at 200 kV, equipped with an Oxford Pentafet Ultrathin Window (UTW) Detector. Before microscopy examination, the sample was ultrasonically dispersed in ethanol for 15-20 min, and then a drop of the suspension was dropped on a copper TEM grid coated with a thin holey carbon film.

X-ray absorption spectra (XAS) including X-ray absorption near edge structure (XANES) and extended X-ray absorption fine structure (EXAFS) at Pt L_{III} edge of the samples were measured at the beamline

14W of Shanghai Synchrotron Radiation Facility (SSRF) in China. The output beam was selected by Si (111) monochromator, and the energy was calibrated by Pt foil. The data were collected at room temperature under transmission mode. Athena software package was employed to process the XAS data.

NMR spectra were recorded at room temperature in $CDCl_3$/d^6-DMSO on 300/400 MHz Bruker DRX-300/400 NMR spectrometers.

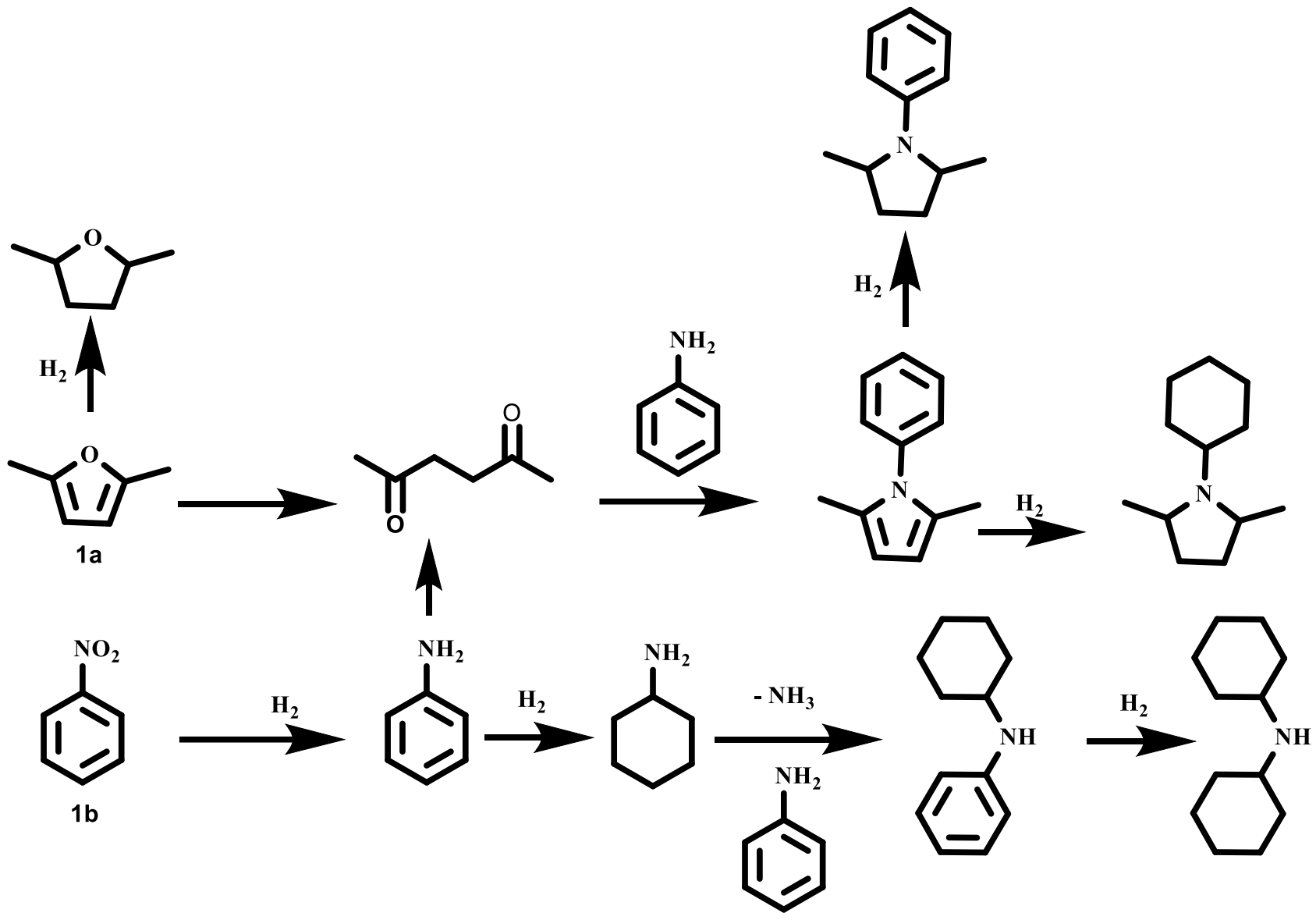

Supplementary Figure 1. The reaction network of transformation of nitroarene and furan in the presence of H_2.

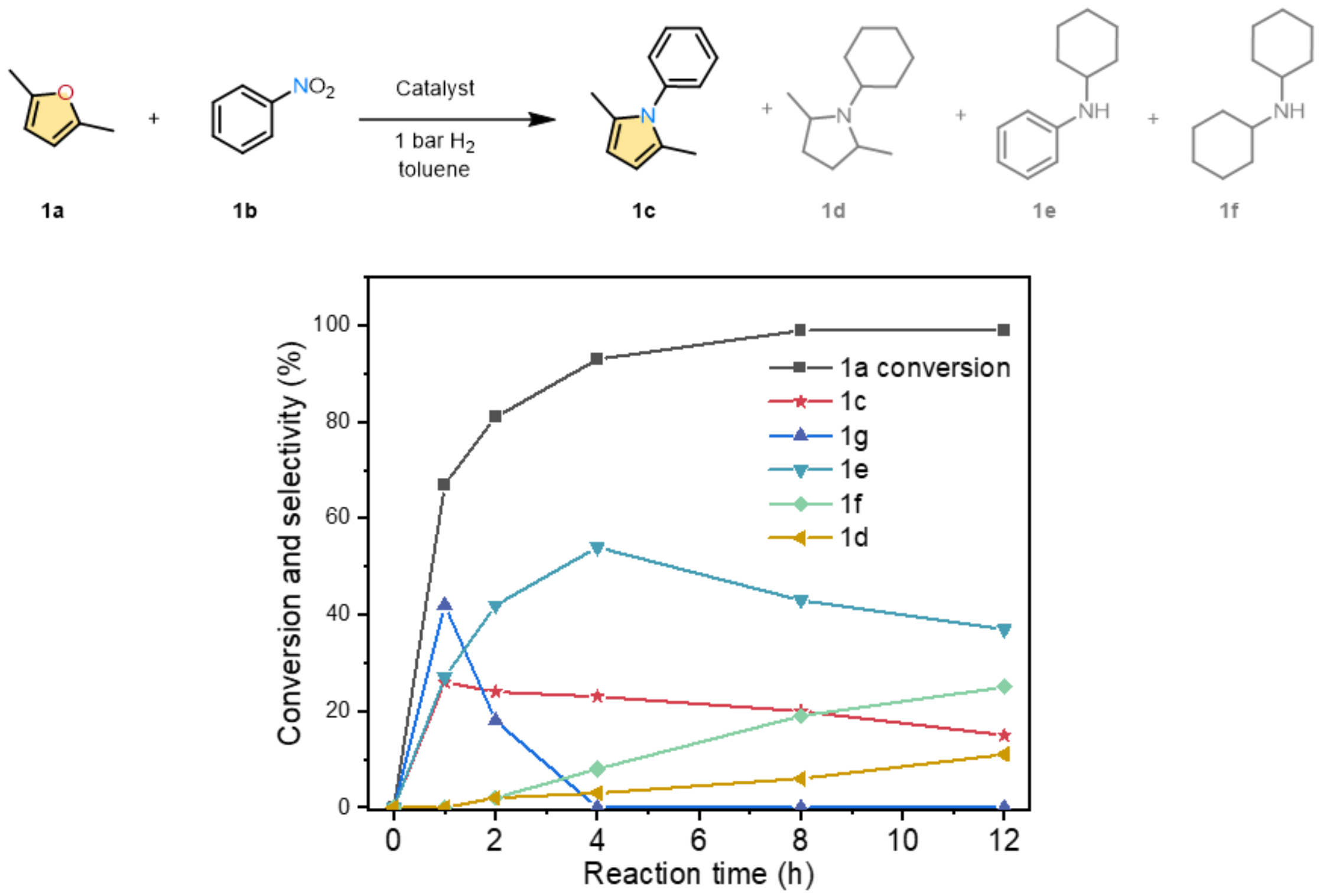

Supplementary Figure 2. Kinetic profile of 0.5Pt/HY catalyst. Standard reaction conditions: 0.25 mmol 1a, 0.25 mmol 1b, 100 mg 0.5Pt/HY catalyst, 1 mL toluene, 1 bar H_2, 20 bar N_2, 130 °C, defined time.

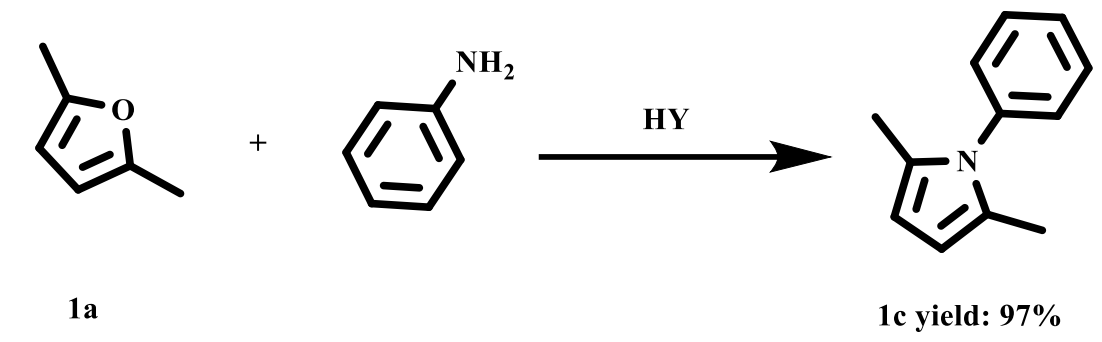

Supplementary Figure 3. Coupling reaction of aniline and 2,5-dimethylfuran in the presence of HY zeolite. Standard reaction conditions: 0.25 mmol 2,5-dimethylfuran **1a**, 0.25 mmol aniline, 100 mg HY, 1 mL toluene, 20 bar N_2, 130 °C, 12 h.

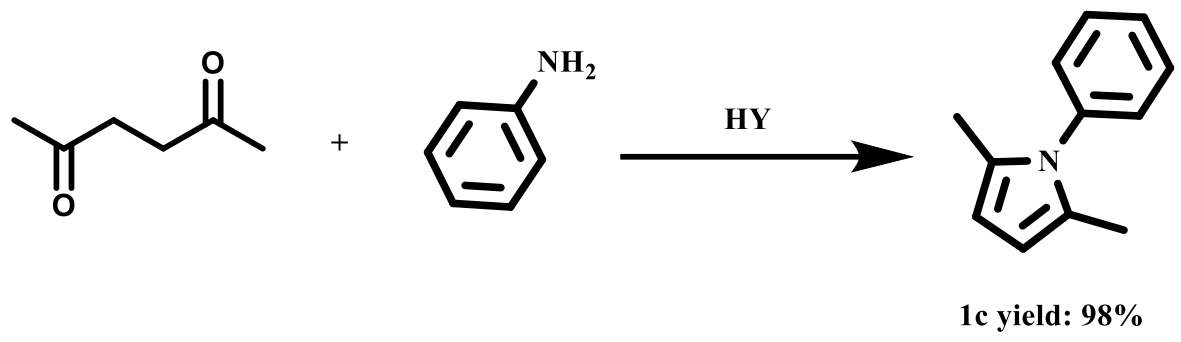

Supplementary Figure 4. Coupling reaction of 2,5-hexanedione and aniline in the presence of HY zeolite. Standard reaction conditions: 0.25 mmol 2,5-hexanedione, 0.25 mmol aniline, 100 mg HY, 1 mL toluene, 20 bar N_2, 130 °C, 12 h.

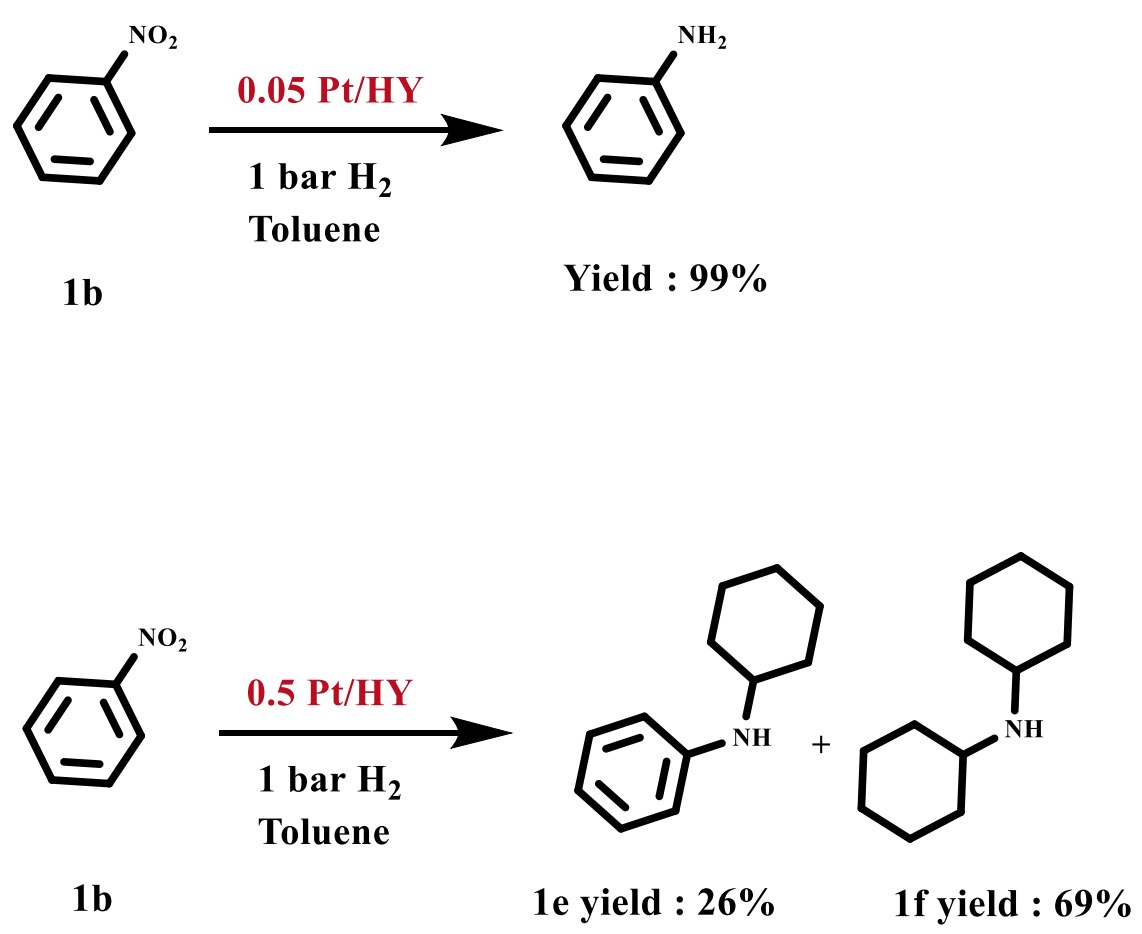

Supplementary Figure 5. Hydrogenation of nitrobenzene using 0.05Pt/HY and 0.5Pt/HY as catalysts. Reaction conditions: 0.25 mmol nitrobenzene **1b**, 0.02 mol% Pt relative to nitrobenzene, 1 mL toluene, 1 bar H_2, 20 bar N_2, 130 °C, 12 h.

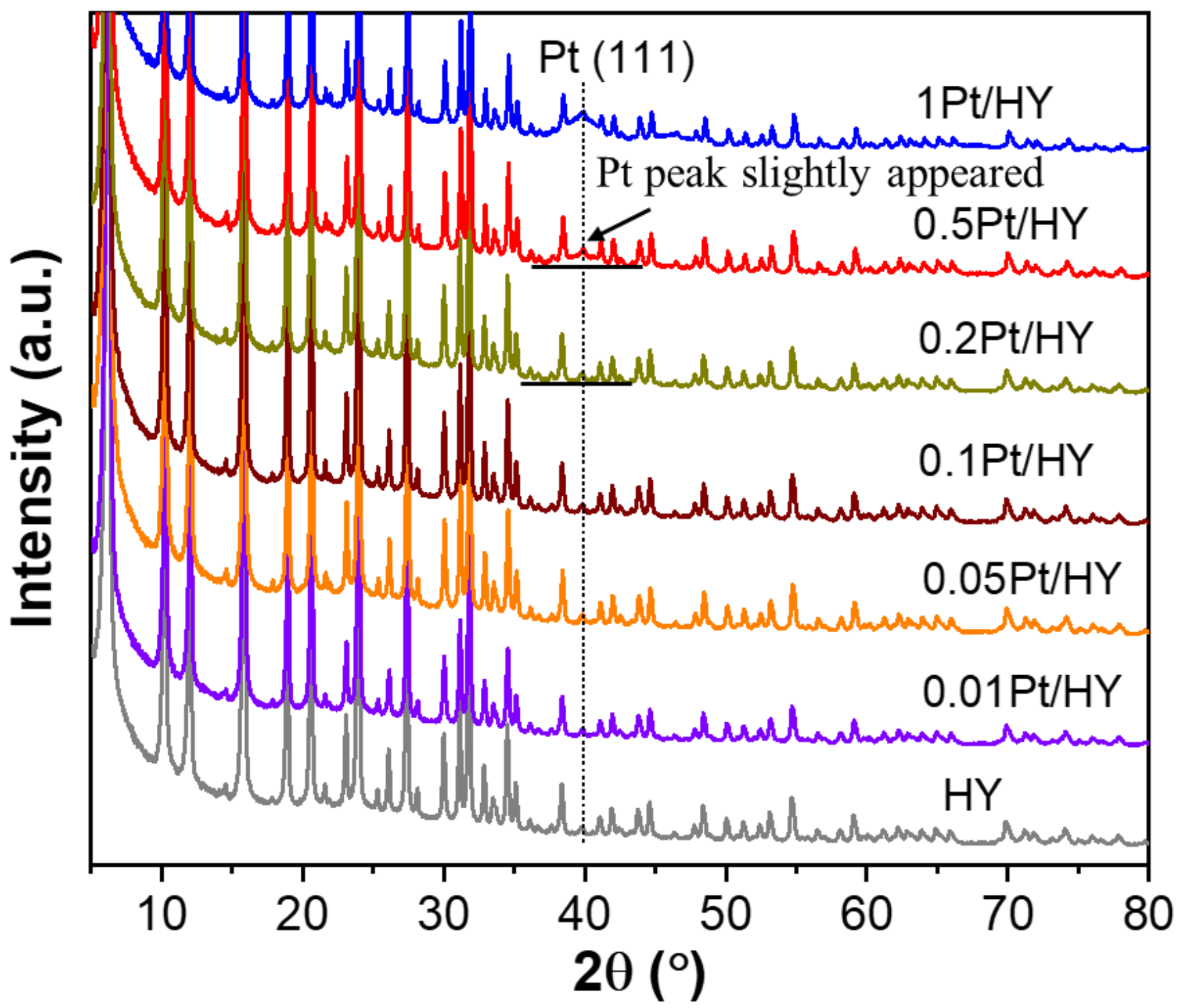

Supplementary Figure 6. XRD patterns of different Pt-loading Pt/HY catalysts.

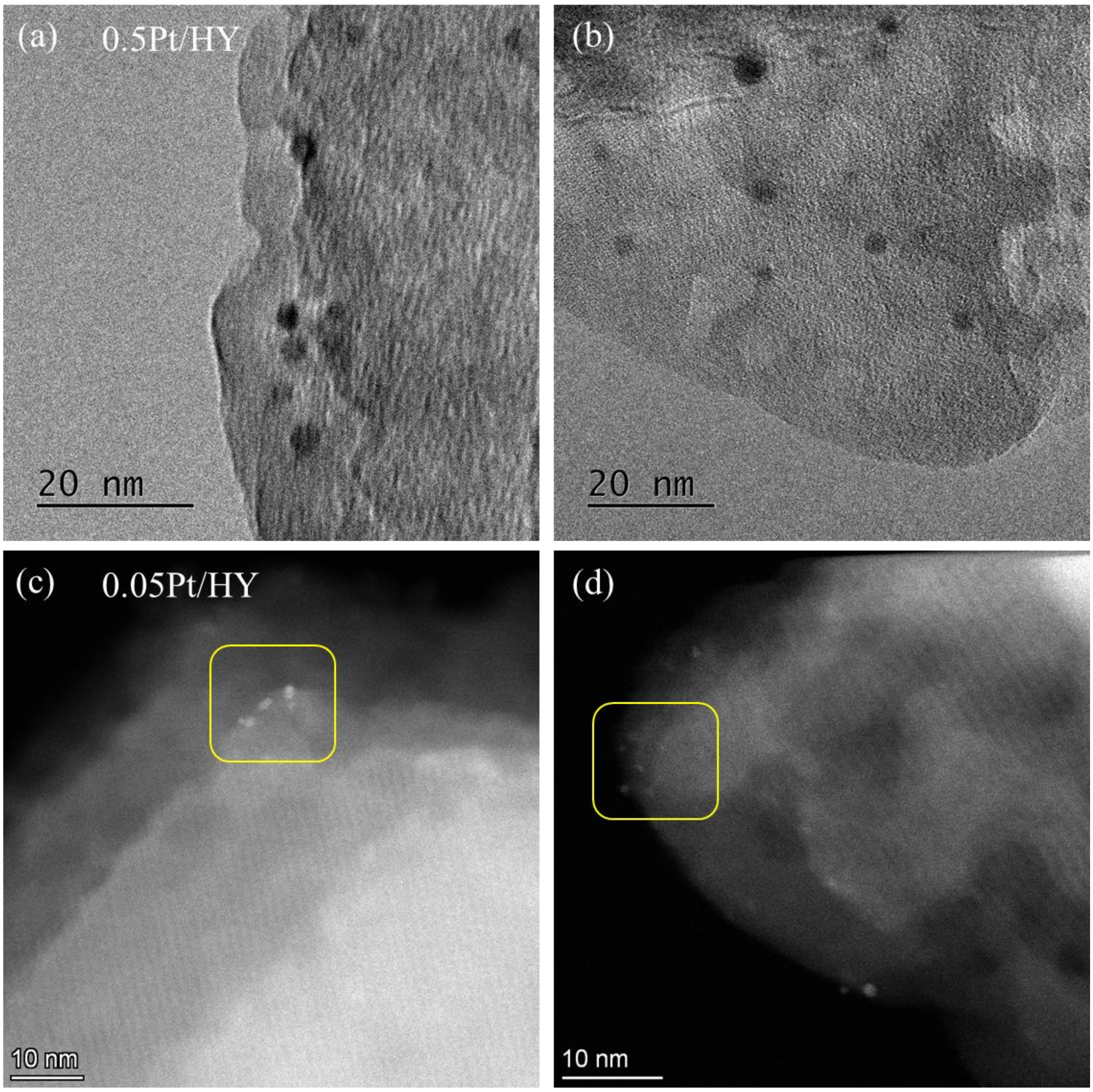

Supplementary Figure 7. AC STEM images of 0.5Pt/HY (a-b) and 0.05Pt/HY (c-d) catalysts.

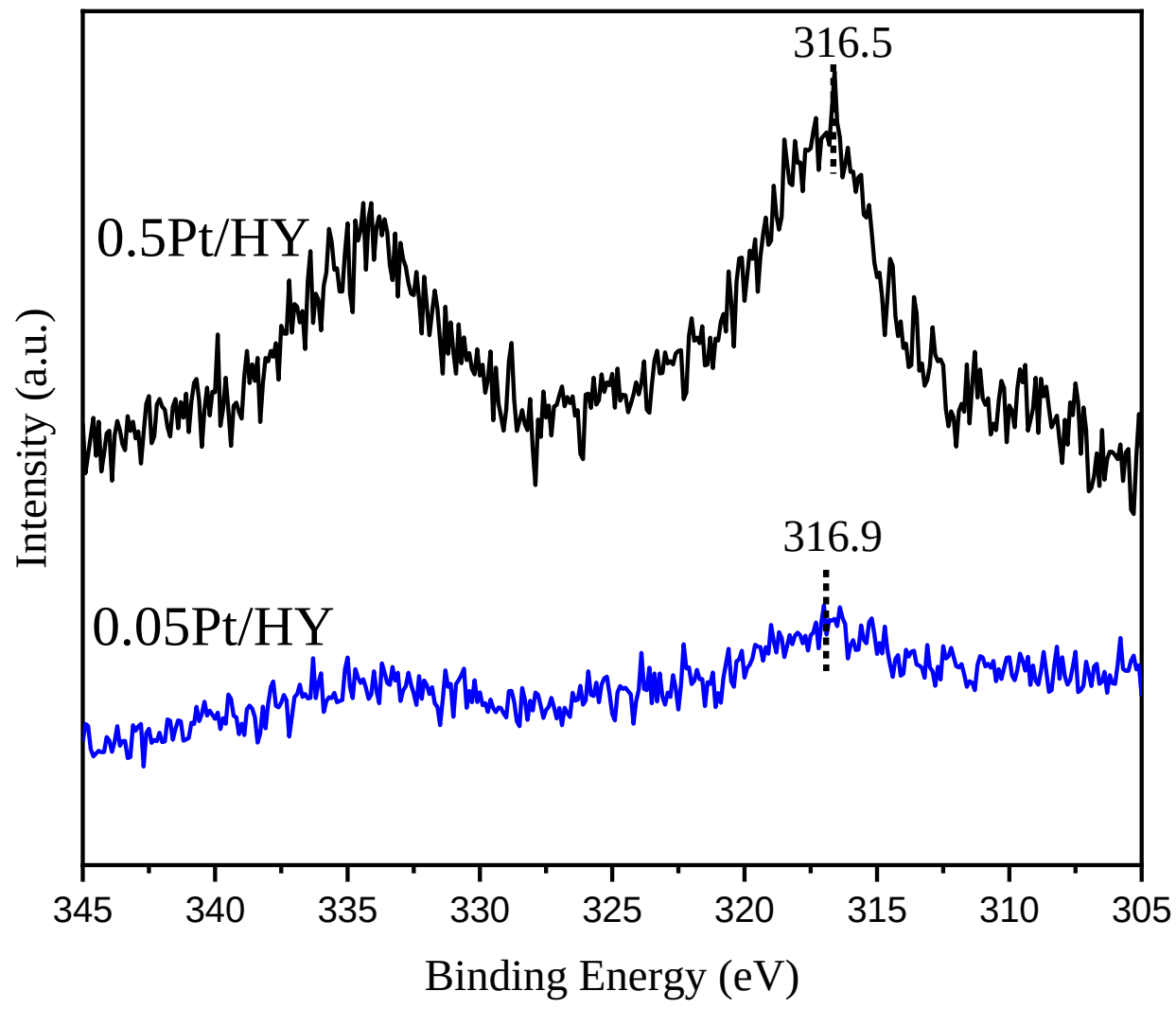

Supplementary Figure 8. Pt *4d* X-ray photoelectron spectroscopy of 0.05Pt/HY and 0.5Pt/HY catalysts.

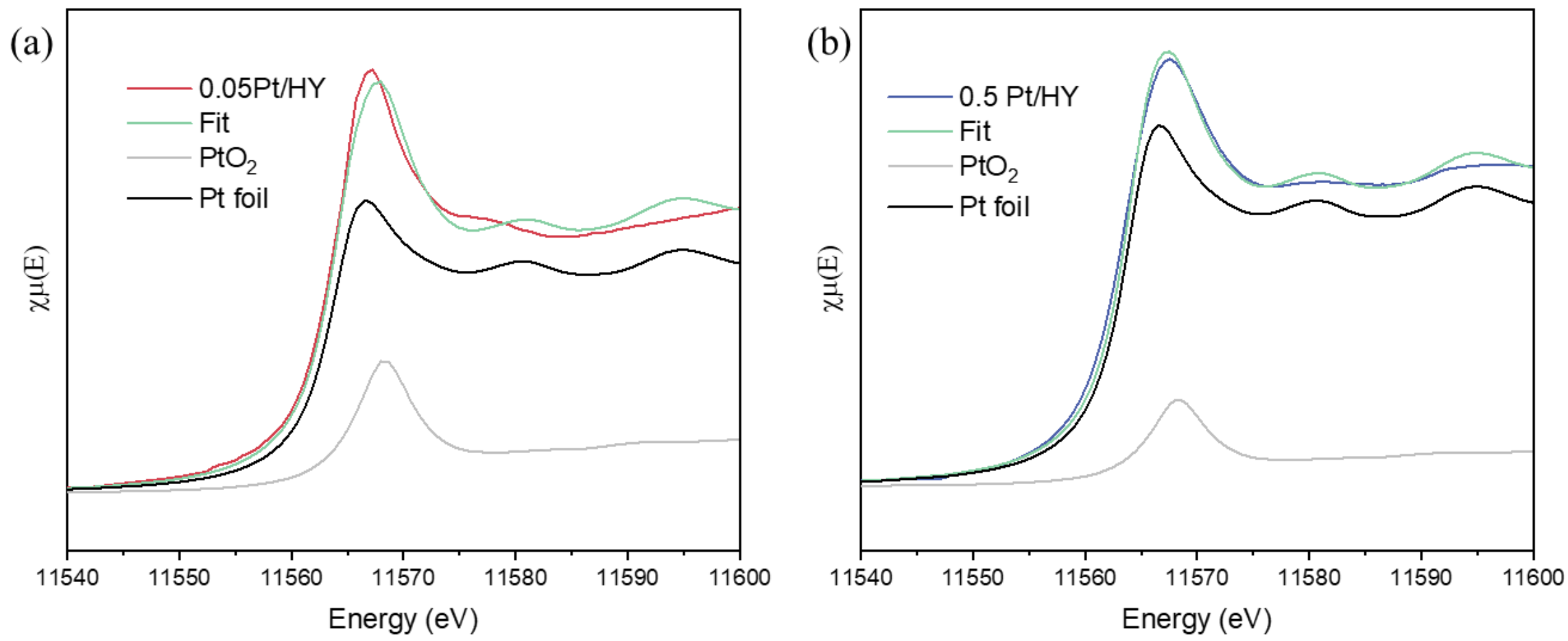

Supplementary Figure 9. Linear combination fitting analysis of X-ray absorption near-edge structure spectra for (a) 0.05Pt/HY and (b) 0.5Pt/HY catalysts.

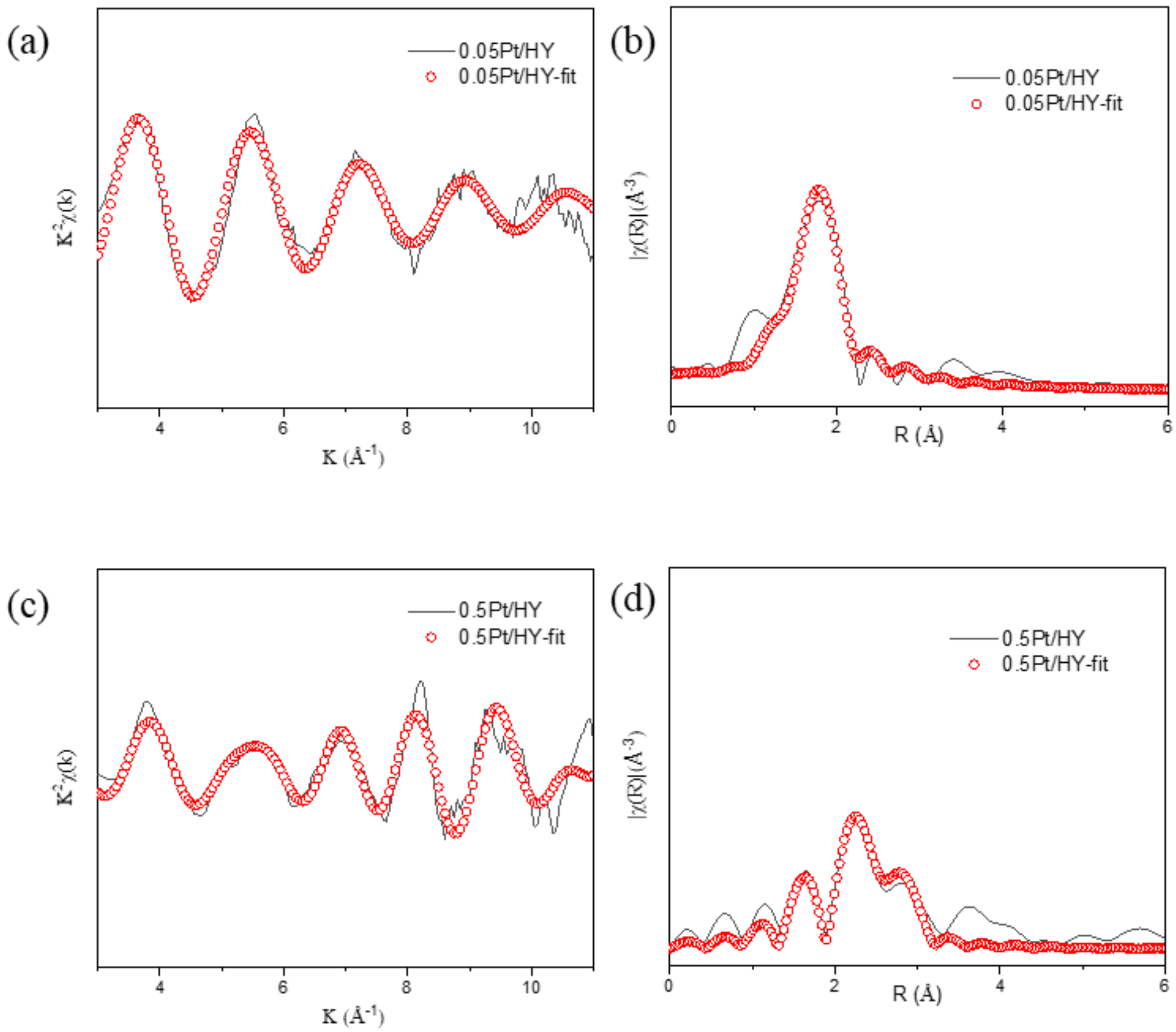

Supplementary Figure 10. EXAFS fitting curve of k^2 weighted *k*-space (a) and *R*-space (b) in 0.05Pt/HY catalyst; EXAFS fitting curve of k^2 weighted *k*-space (c) and *R*-space (d) in 0.5Pt/HY catalyst.

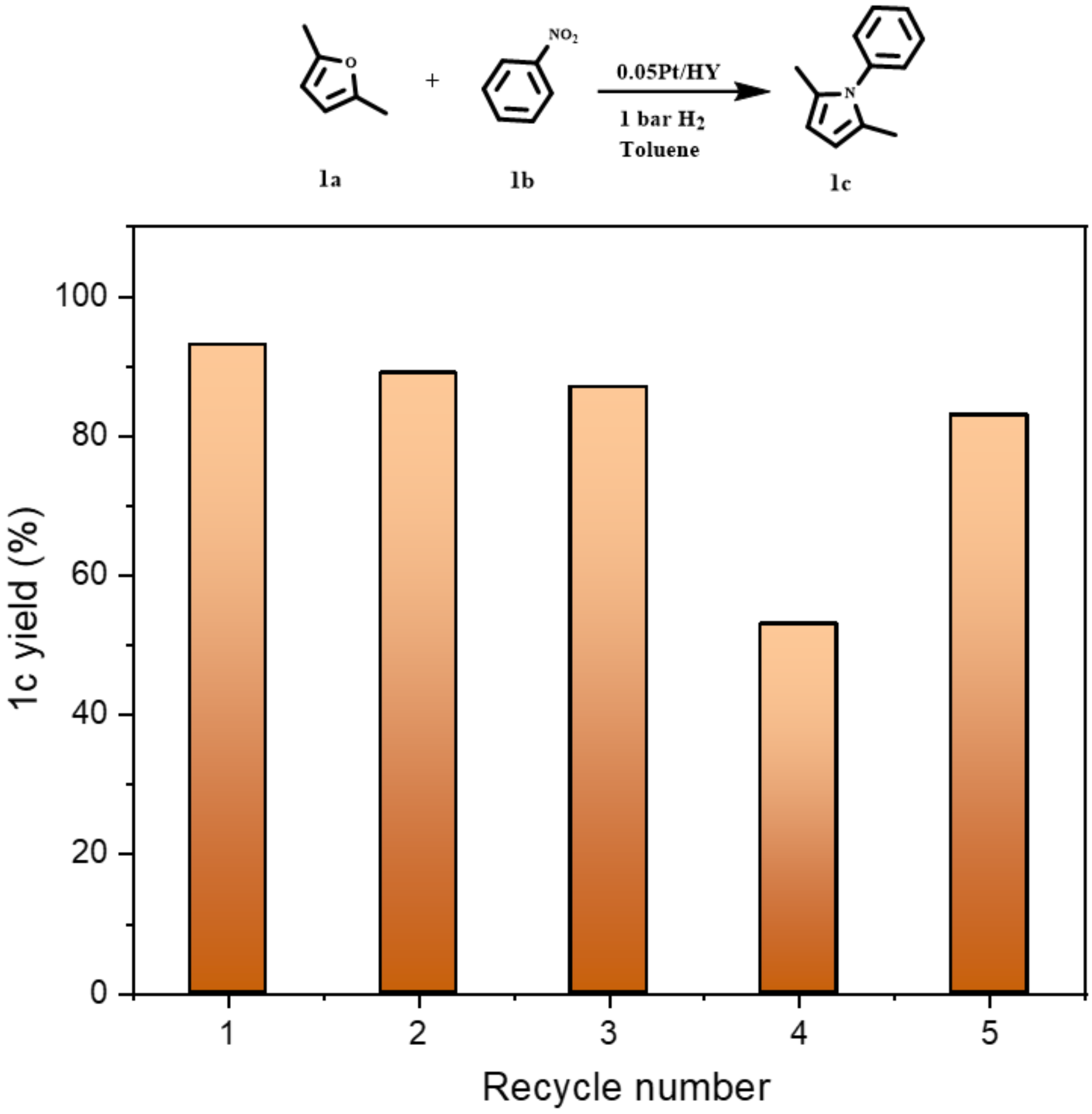

Supplementary Figure 11. Recyclability of 0.05Pt/HY catalyst. Reaction condition: 0.25 mmol 1a, 0.25 mmol 1b, 100 mg catalyst, 1 mL toluene, 1 bar H_2, 20 bar N_2, 130 °C, 12 h; After each reaction, the catalyst was recovered by filtration and washed with water and ethanol, dried in 60 °C for 6 h, then submitted to the next standard batch reaction. Before the 5th reaction, the catalyst recycled from the 4th reaction was immersed in a 0.01 mmol·L^{-1} HNO_3 solution, then washed and dried.

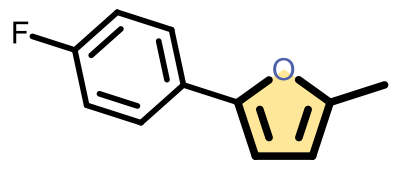

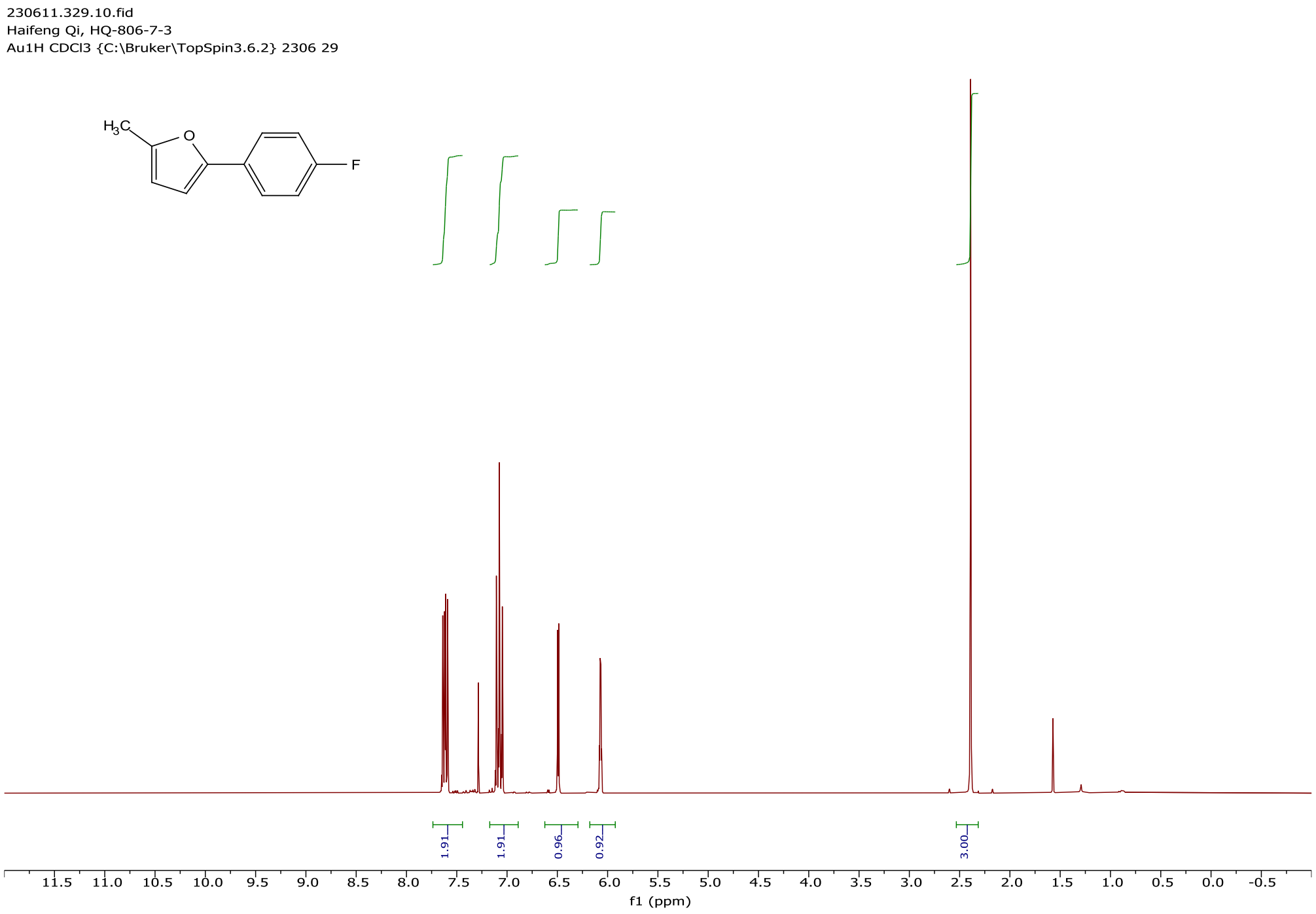

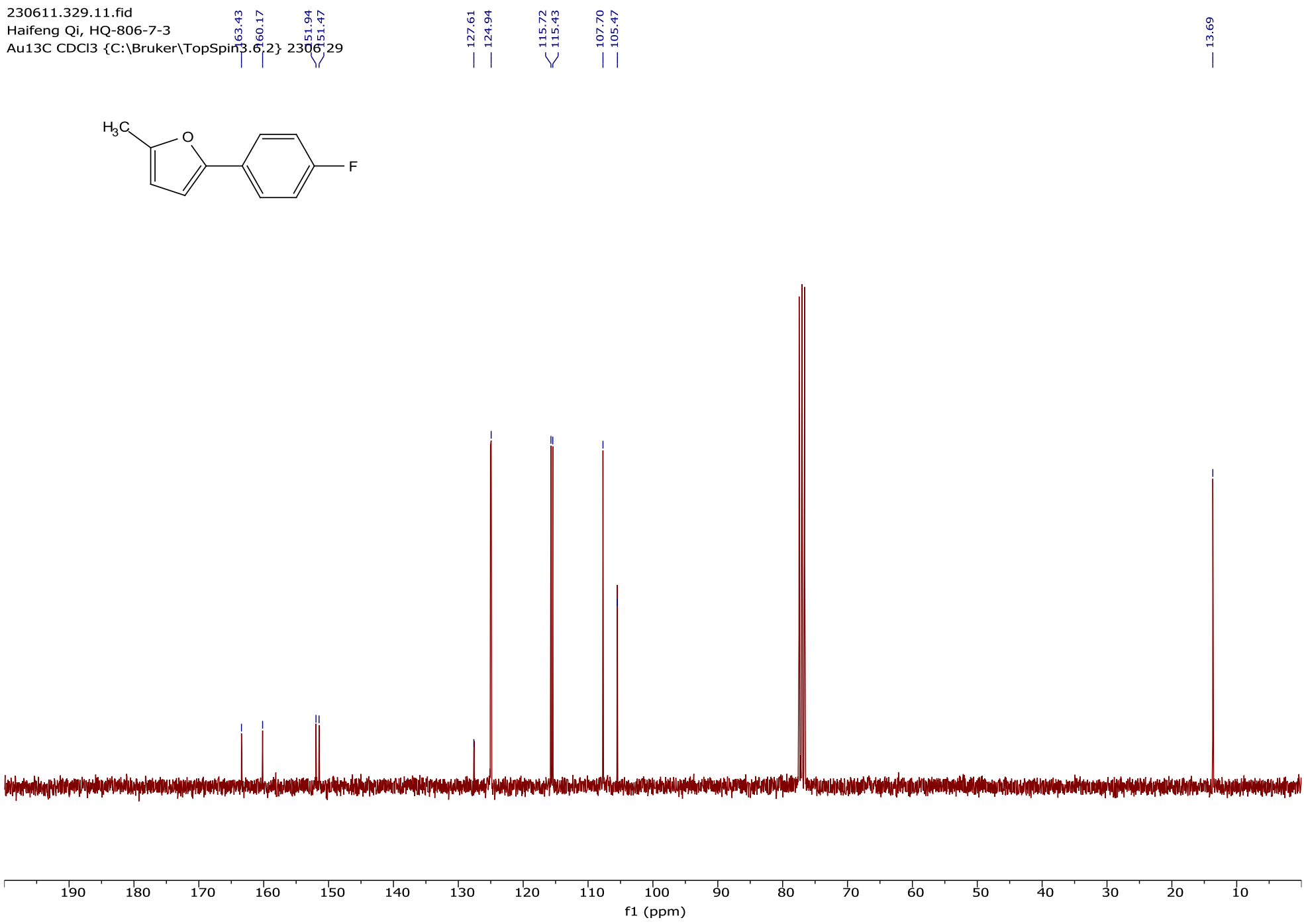

Supplementary Figure 12. ^{1}H and ^{13}C NMR spectra of product **34d**.

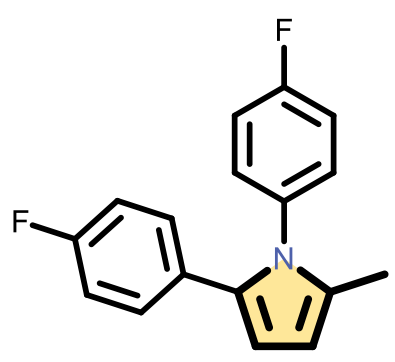

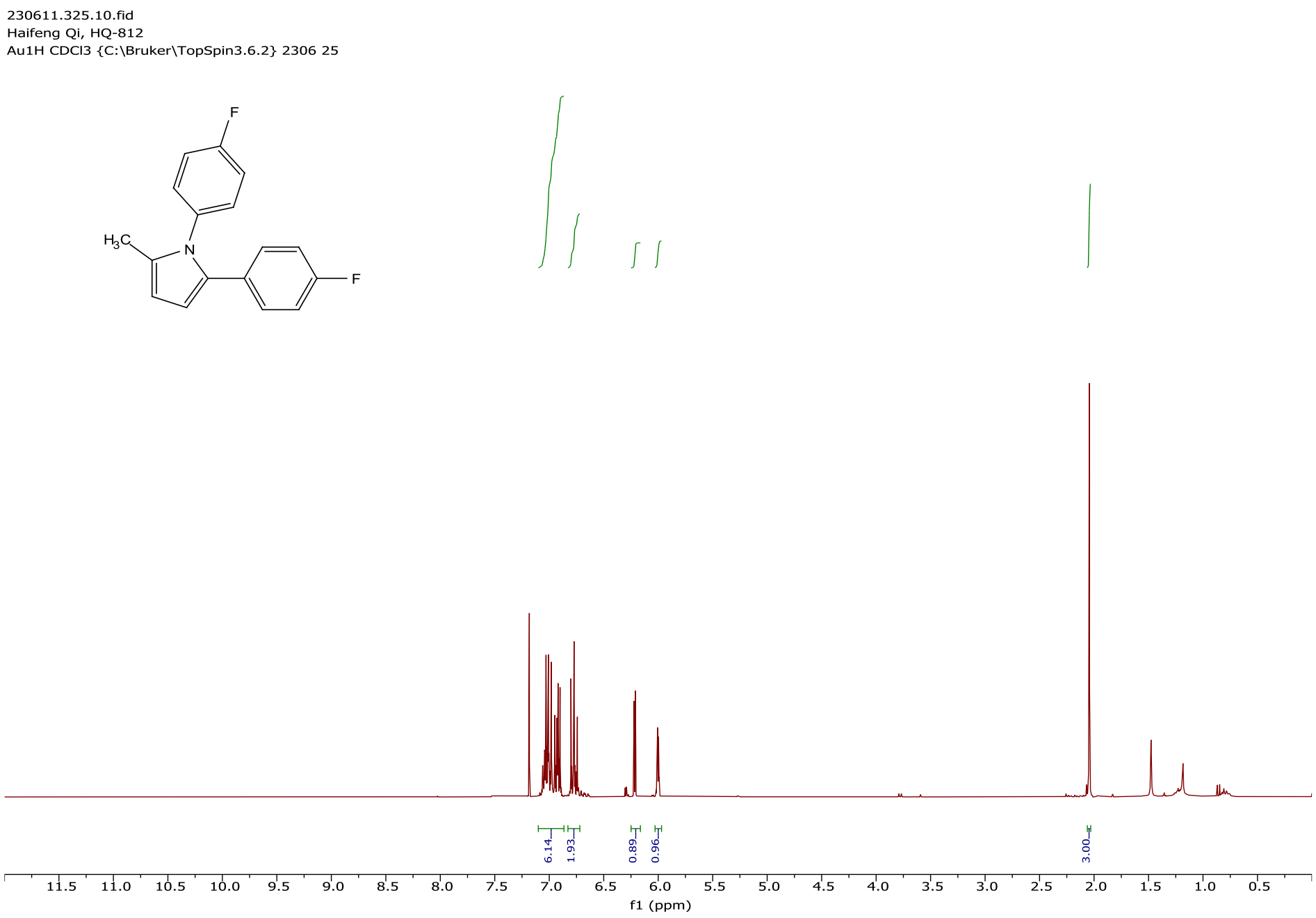

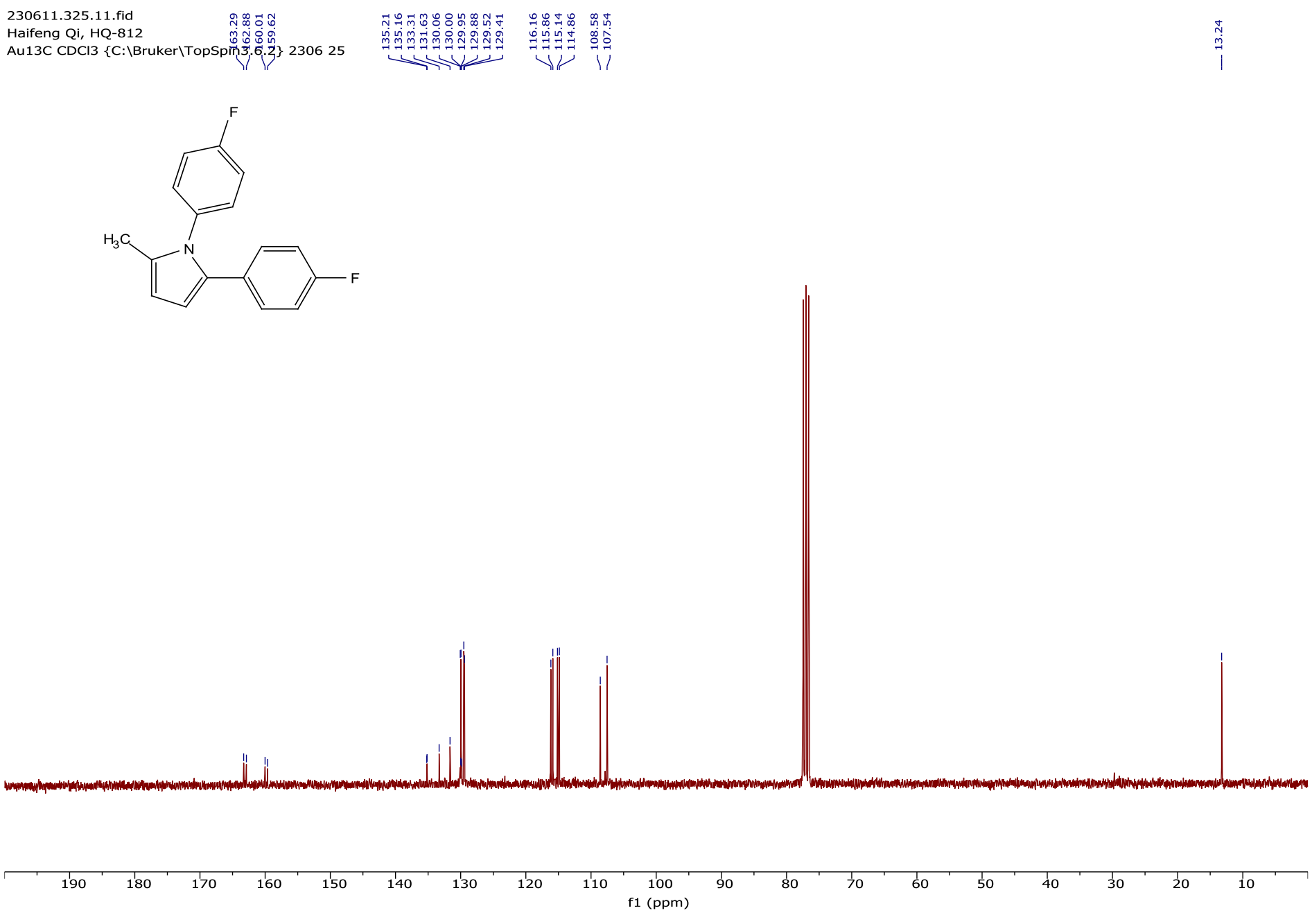

Supplementary Figure 13. ^{1}H and ^{13}C NMR spectra of product **34e**.

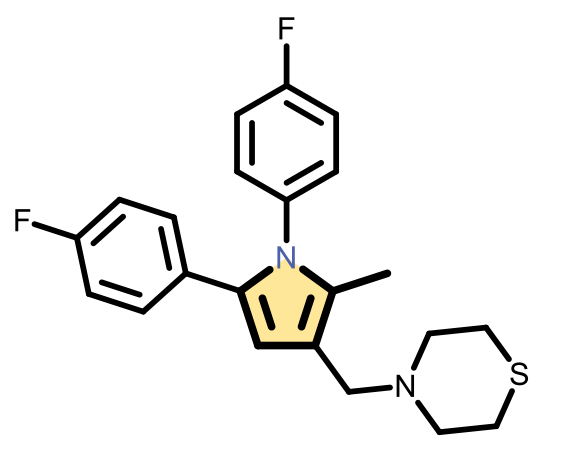

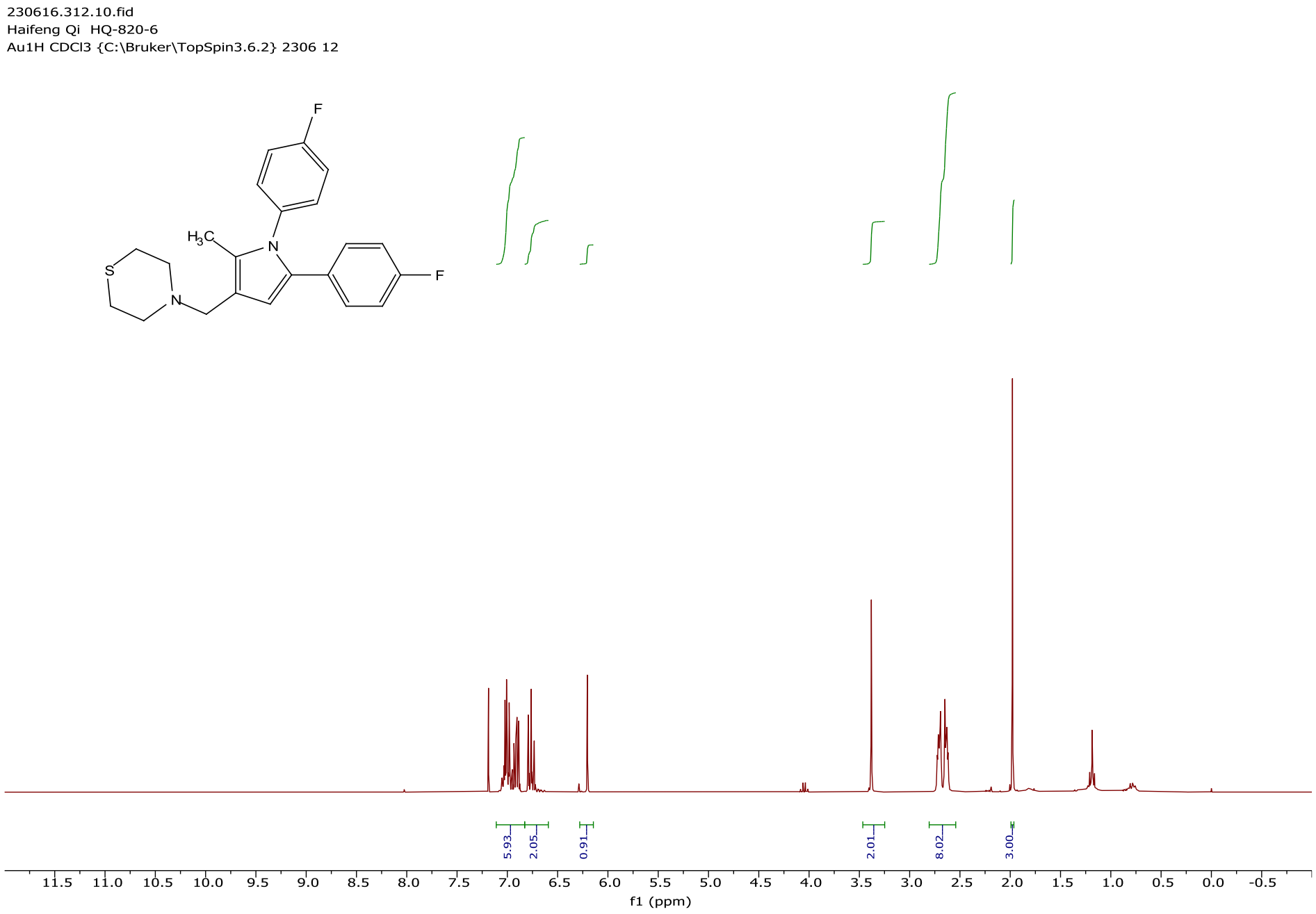

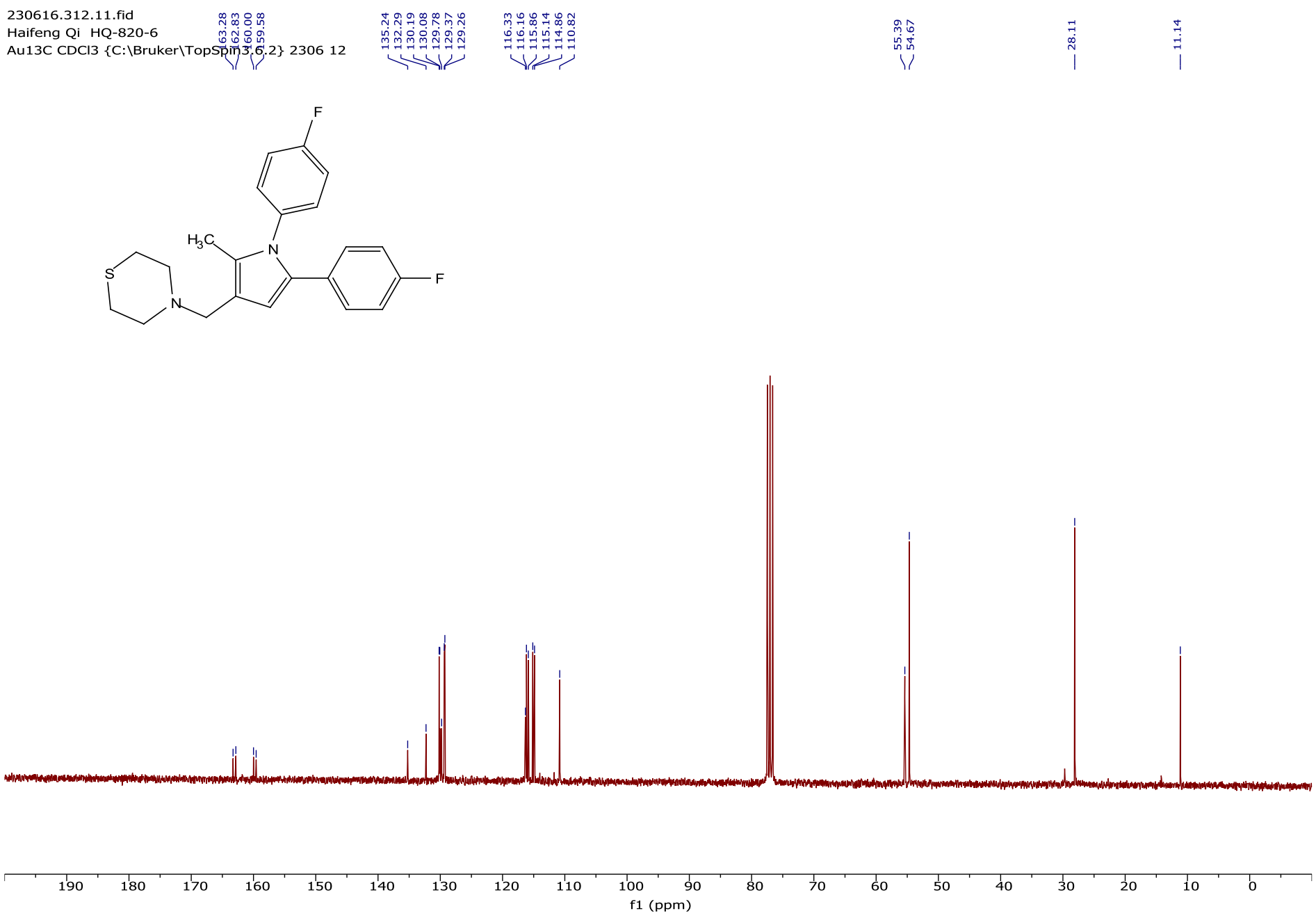

Supplementary Figure 14. ^{1}H and ^{13}C NMR spectra of product **34c**.

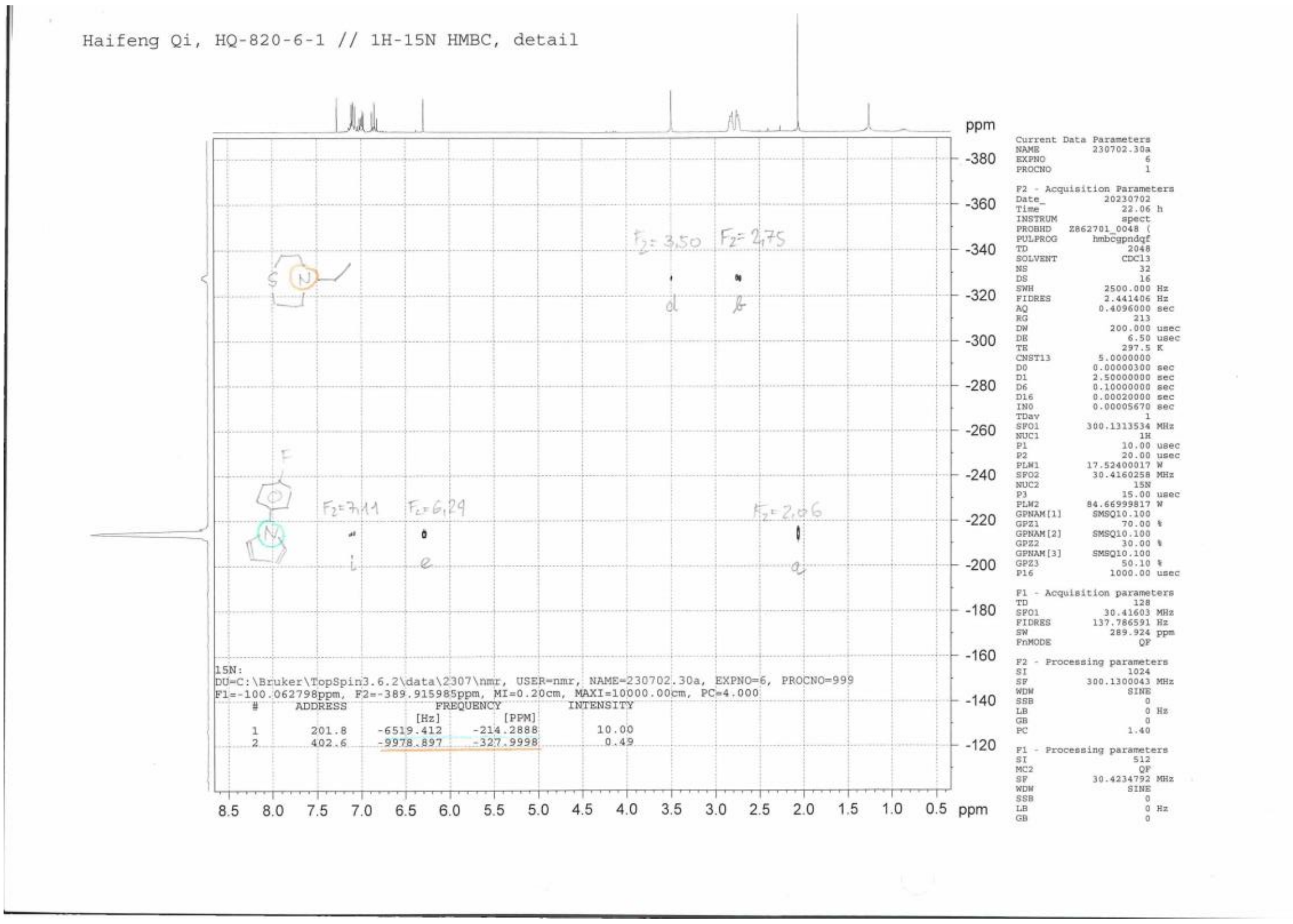
Haifeng Qi, HQ-820-6-1 // 1H-15N HMBC, detail

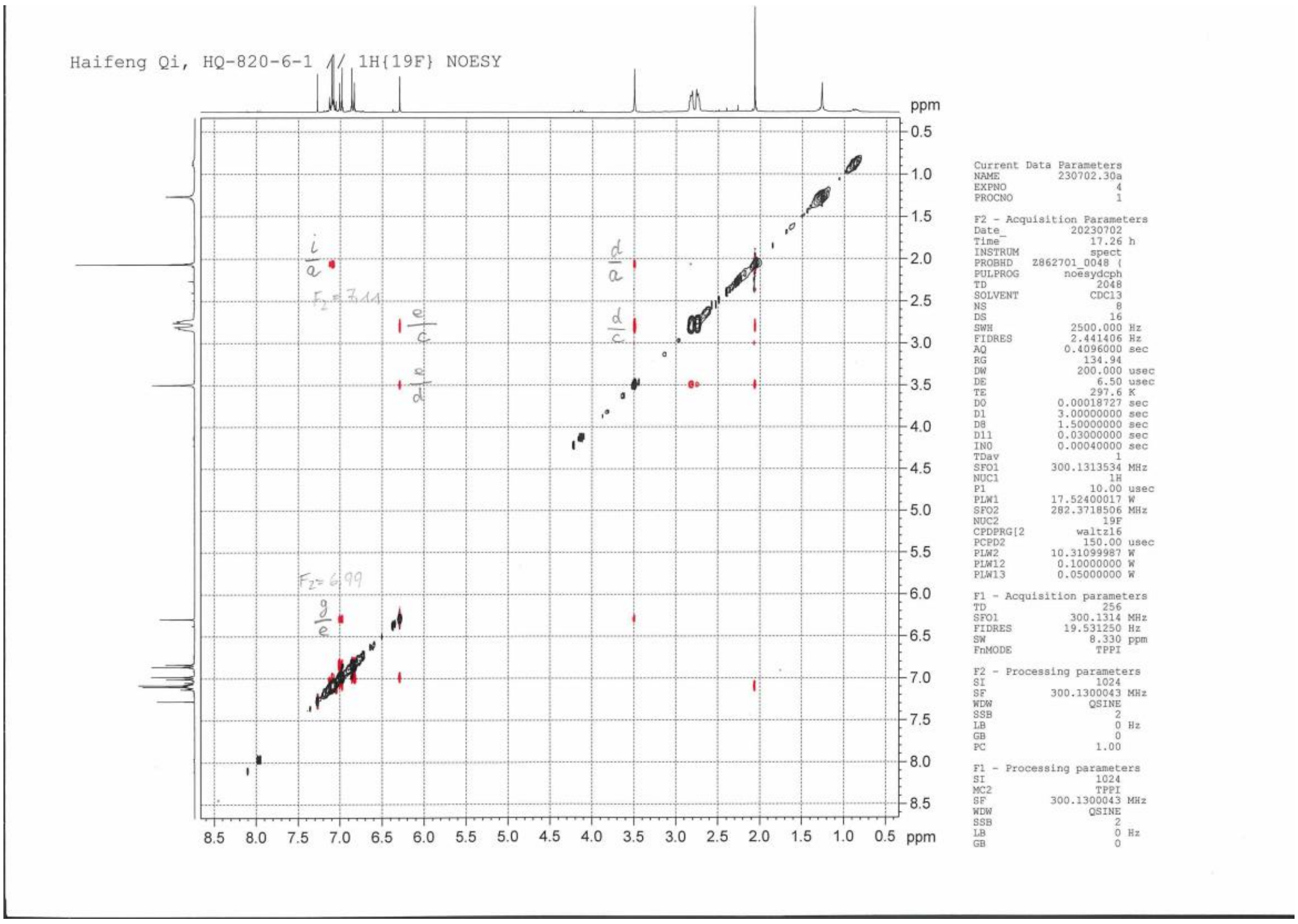
Haifeng Qi, HQ-820-6-1 // 1H{19F} NOESY

06.2023 11:21 MassLib

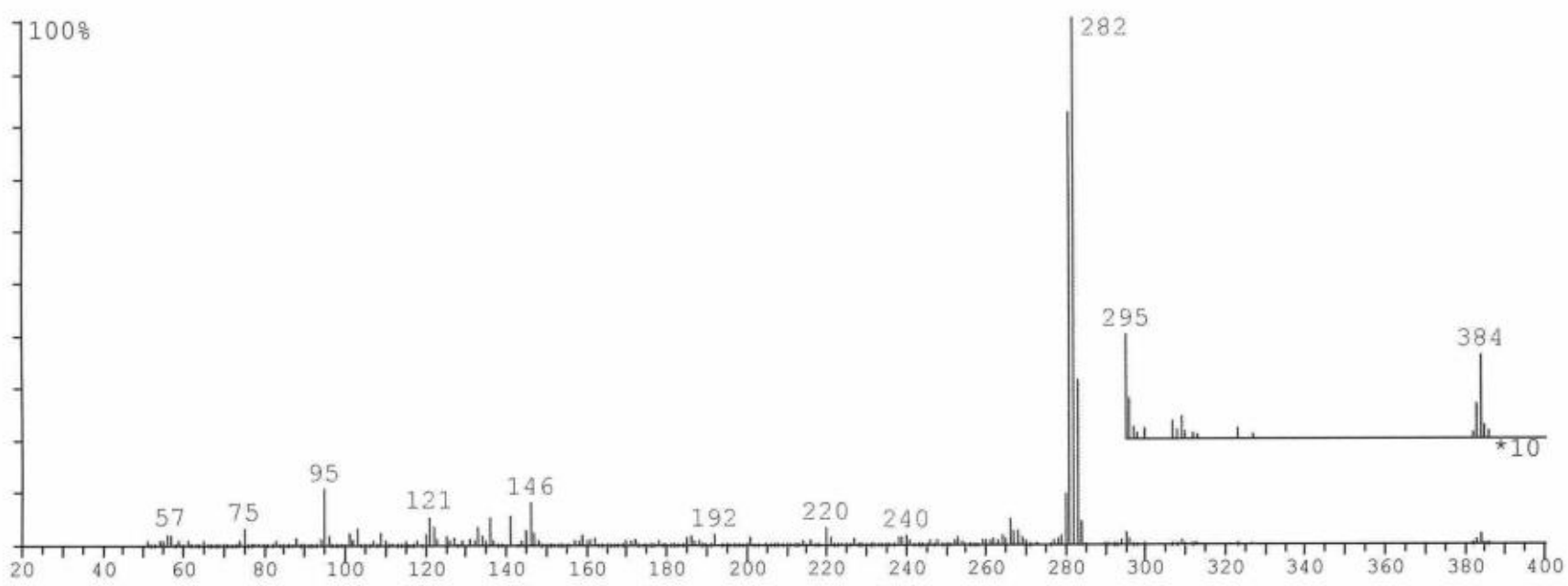

B4352(32:19) 2934 26% 23062705 * MS9-Haifeng Qi HQ-820-6
23062705

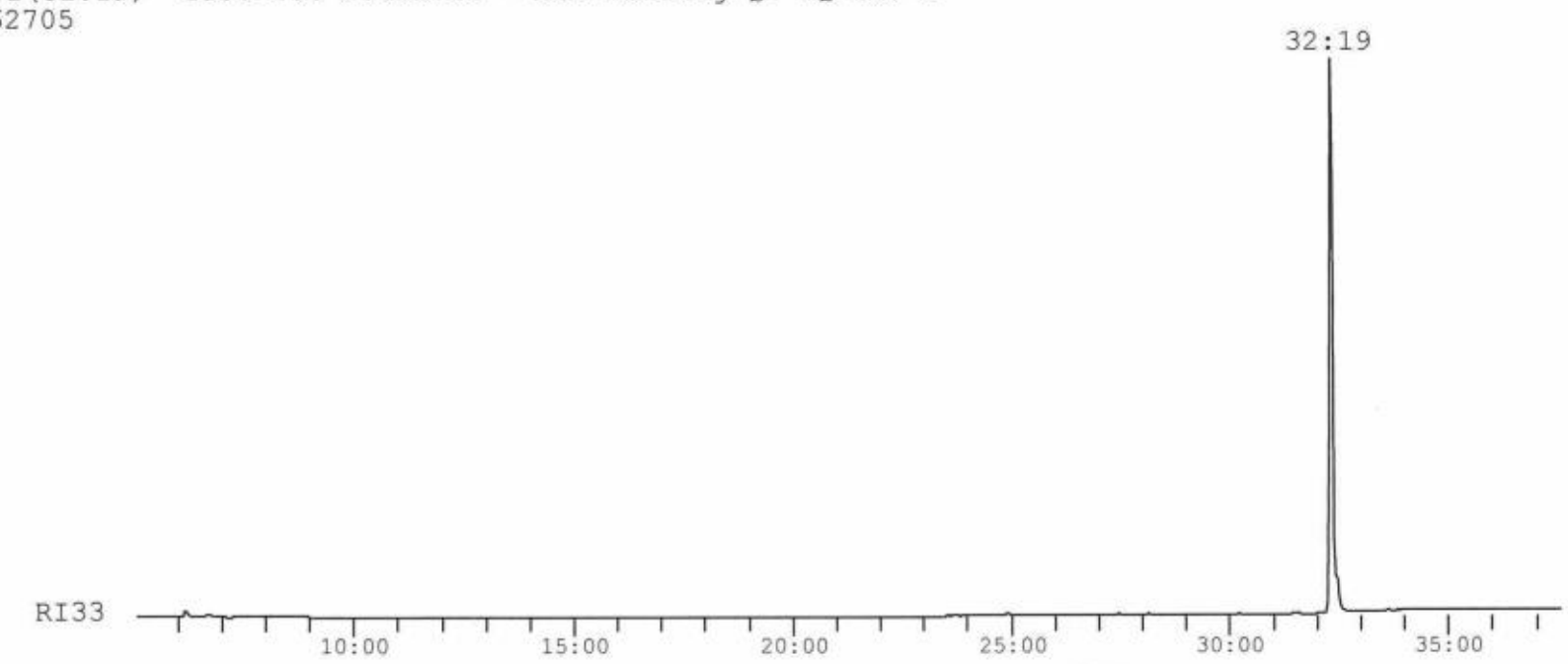

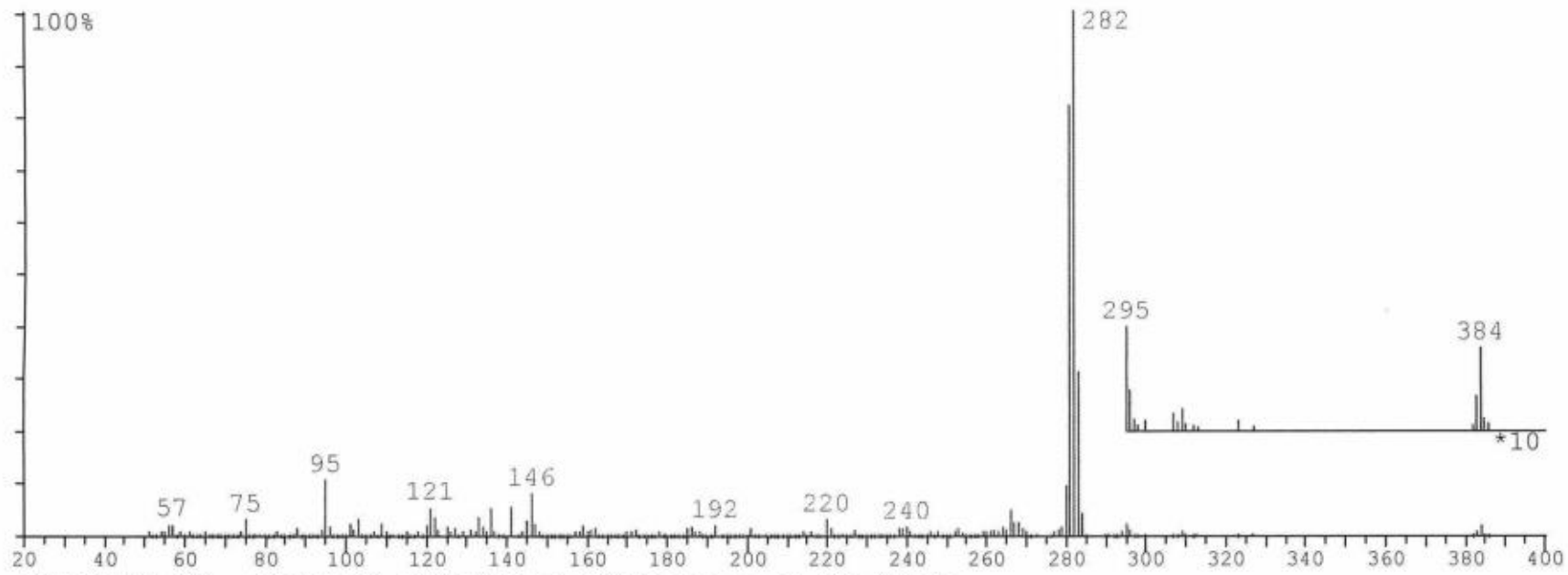

B4352(32:19) 2934 26% 23062705 * MS9-Haifeng Qi HQ-820-6

51	0.43	94	0.76	125	1.45	158	0.42	200	0.25	242	0.29	270	0.38
54	0.50	95	10.33	126	0.52	159	1.55	201	0.96	244	0.26	277	0.63
55	0.66	96	1.50	127	0.98	160	0.60	202	0.29	245	0.25	278	0.69
56	1.74	99	0.31	128	0.26	161	0.75	207	0.26	246	0.63	279	1.26
57	1.80	101	2.06	129	0.49	162	1.15	213	0.24	247	0.26	280	9.17
58	0.34	102	0.69	131	0.91	164	0.24	214	0.62	248	0.39	281	81.97
59	0.51	103	2.75	132	0.55	169	0.33	216	0.54	251	0.31	282	100.00
60	0.36	104	0.26	133	2.99	170	0.47	218	0.24	252	0.49	283	31.06
61	0.58	107	0.63	134	1.26	171	0.57	220	2.92	253	0.97	284	3.89
63	0.29	108	0.35	135	0.49	172	0.85	221	0.99	254	0.37	285	0.31
65	0.42	109	1.93	136	4.83	173	0.24	222	0.30	259	0.51	293	0.29
68	0.23	110	0.40	137	0.64	178	0.63	225	0.31	260	0.55	294	0.50
69	0.33	114	0.30	139	0.29	183	0.31	227	0.69	261	0.39	295	1.97
70	0.26	115	0.53	141	5.14	184	0.36	228	0.25	262	0.70	296	0.76
73	0.29	116	0.26	144	0.50	185	1.21	229	0.30	263	0.41	307	0.31
74	0.58	118	0.44	145	2.53	186	1.39	233	0.31	264	1.27	309	0.41
75	2.80	119	0.27	146	7.71	187	0.57	235	0.27	265	0.82	383	0.64
76	0.34	120	1.60	147	2.06	188	0.64	238	1.16	266	4.68	384	1.57
83	0.42	121	5.00	148	0.66	192	1.58	239	0.99	267	2.35		
87	0.35	122	3.03	155	0.27	194	0.23	240	1.30	268	2.37		
88	1.03	123	0.66	157	0.46	199	0.23	241	0.41	269	1.05		

B4352(32:19)2934 26% 23062705 * MS9-Haifeng Qi HQ-820-6 lim: 0.23%

MassLib V9.4.0 (Print Identifier) IfOK.Rostock

Supplementary Figure 15. 2D NMR and HR-MS spectra of product **34c.**

(a)

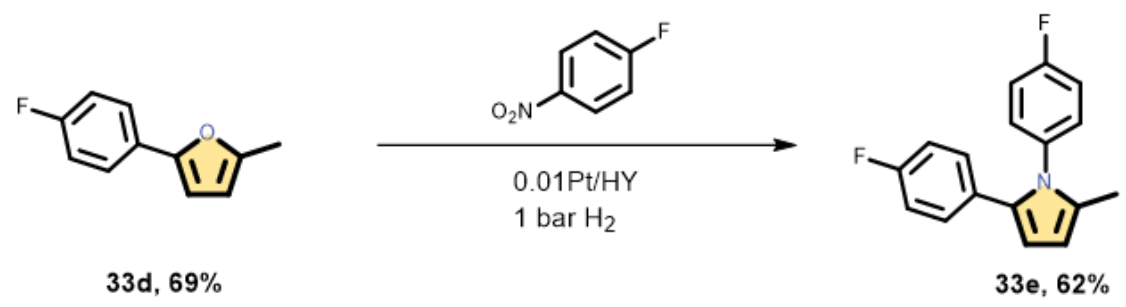

This work

(b)

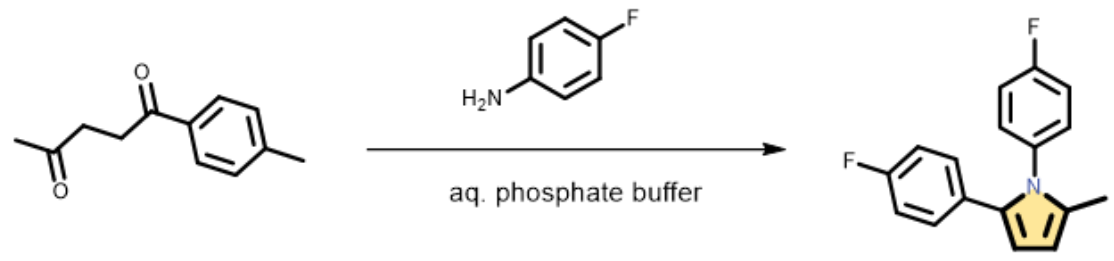

Traditional Paal-Knorr pyrrole synthesis

Supplementary Figure 16. (a) This work using furan as substrate and (b) traditional Paal-Knorr pyrrole synthetic method using diketone as substrate[1].

Table 1. Catalytic condensation of 2,5-dimethylfuran with nitrobenzene over comparable catalysts[a]

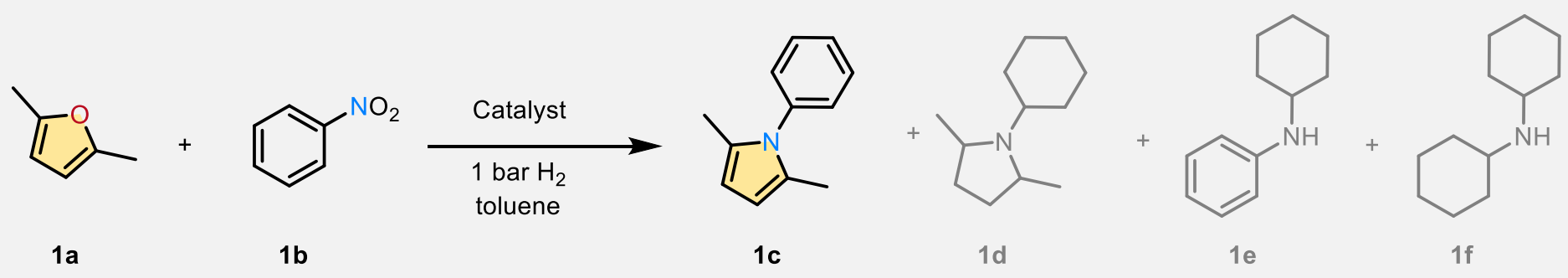

Entry	Catalysts[b]	Yield (%)			
		**1c**	**1d**	**1e**	**1f**
1	0.05Pt/HY (SiO_2/Al_2O_3 = 30)	89	n.d.	3	1
2	0.05Pt/NaY (SiO_2/Al_2O_3 = 5.1)	17	n.d.	65	n.d.

[a] Reaction condition: 0.25 mmol 1a, 0.25 mmol 1b, 100 mg catalyst, 1 mL toluene, 1 bar H_2, 20 bar N_2, 130 °C, 12 h.

Table 2. Linear combination fitting analysis of X-ray absorption near-edge structure spectra.

Sample	Valence ratio (%)		Average valence
	Pt^0	Pt^4	δ^+
0.05Pt/HY	80	20	$\delta = 0.8$
0.5Pt/HY	89	11	$\delta = 0.44$

Table 3. The best-fitted EXAFS results of Pt samples[a].

Sample	Shell	CN	R (Å)	σ^2 (10^{-2} Å^2)	ΔE_0 (eV)	r-factor (%)
Pt foil	Pt-Pt	12*	2.75±0.003	0.4±0.05	5.8±0.5	0.2
0.05Pt/HY	Pt-O	3.4±0.8	2.11±0.02	0.5±0.4	10.8±2.3	1.5
	Pt-Pt	1.1±0.3	2.67±0.03	0.5±0.4	10.8±2.3	
0.5Pt/HY	Pt-O	0.5±0.1	2.11±0.01	1.1±0.3	-2.0±1.7	0.6
	Pt-Pt	5.6±0.5	2.72±0.01	0.7±0.1	-2.0±1.7	

[a]CN is the coordination number for the absorber-backscatterer pair, R is the average absorber-backscatterer distance, σ^2 is the Debye-Waller factor, and ΔE_0 is the inner potential correction. *S_0^2 was fixed to 0.75 as determined from Pt foil fitting. the data range used for data fitting in k-space (Δk) and R-space (ΔR) are 3.0-10.6 Å^{-1} and 1.2-3.2 Å.

Supplementary References

1. B. Wagh, S.; Maslivetc, V.; J. La Clair, J.; Kornienko, A. A fluorescent target-guided Paal–Knorr reaction. *RSC Adv.*, **2020**, *10*, 37035.

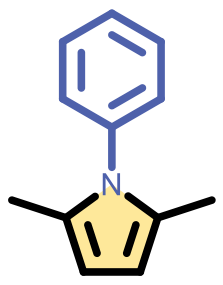

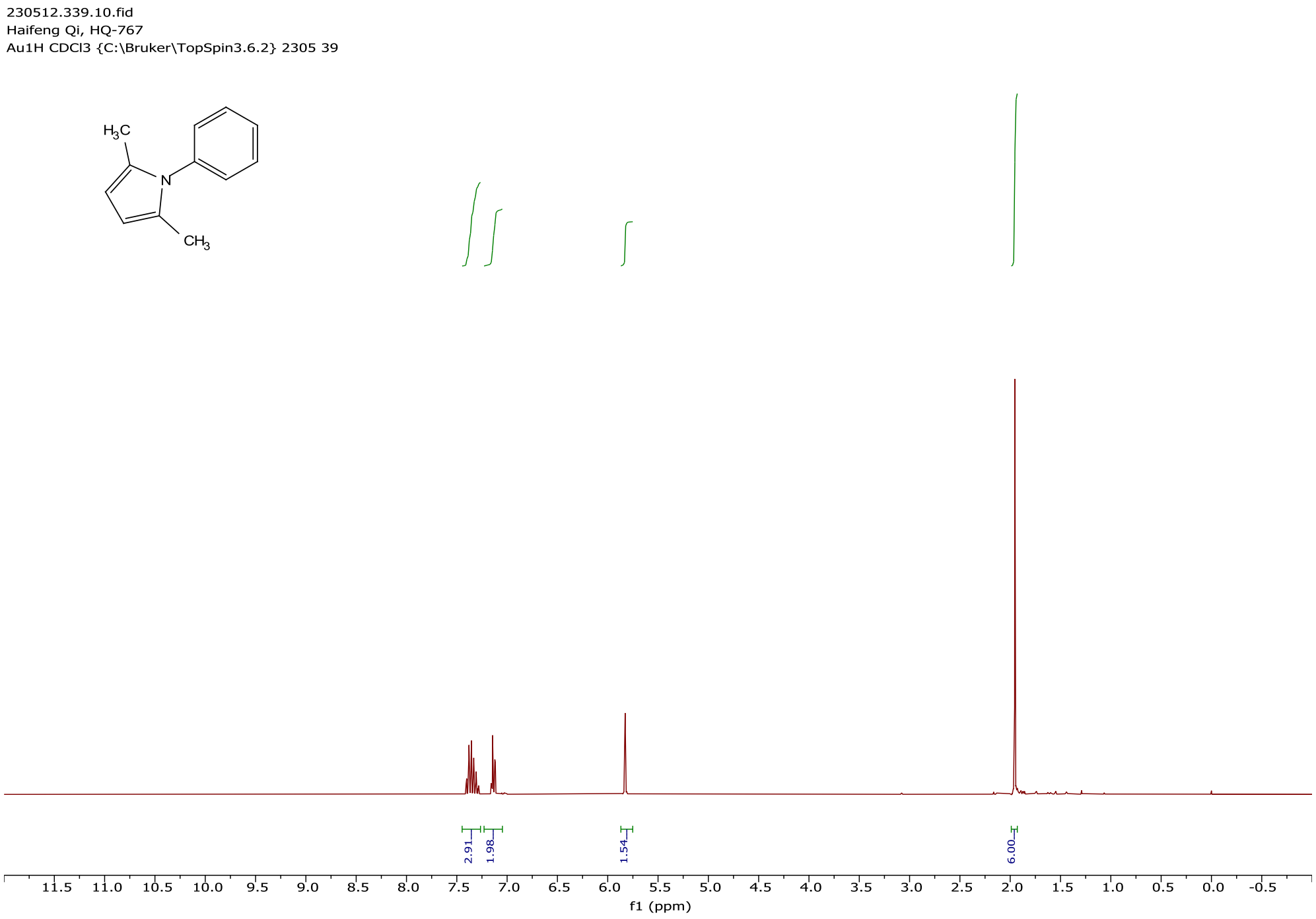

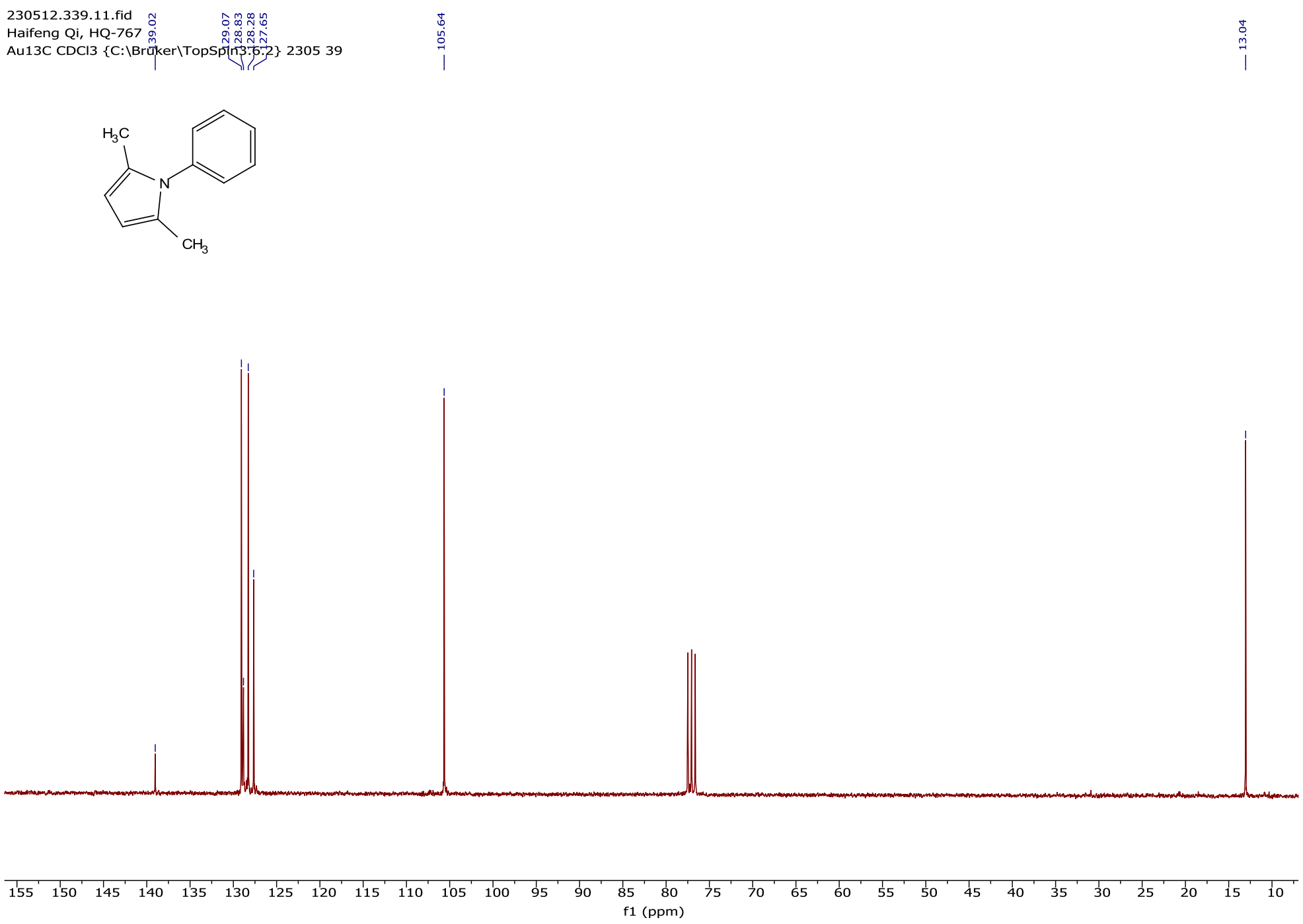

Supplementary Figure 17. ^{1}H and ^{13}C NMR spectra of product **1c**.

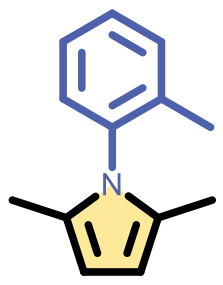

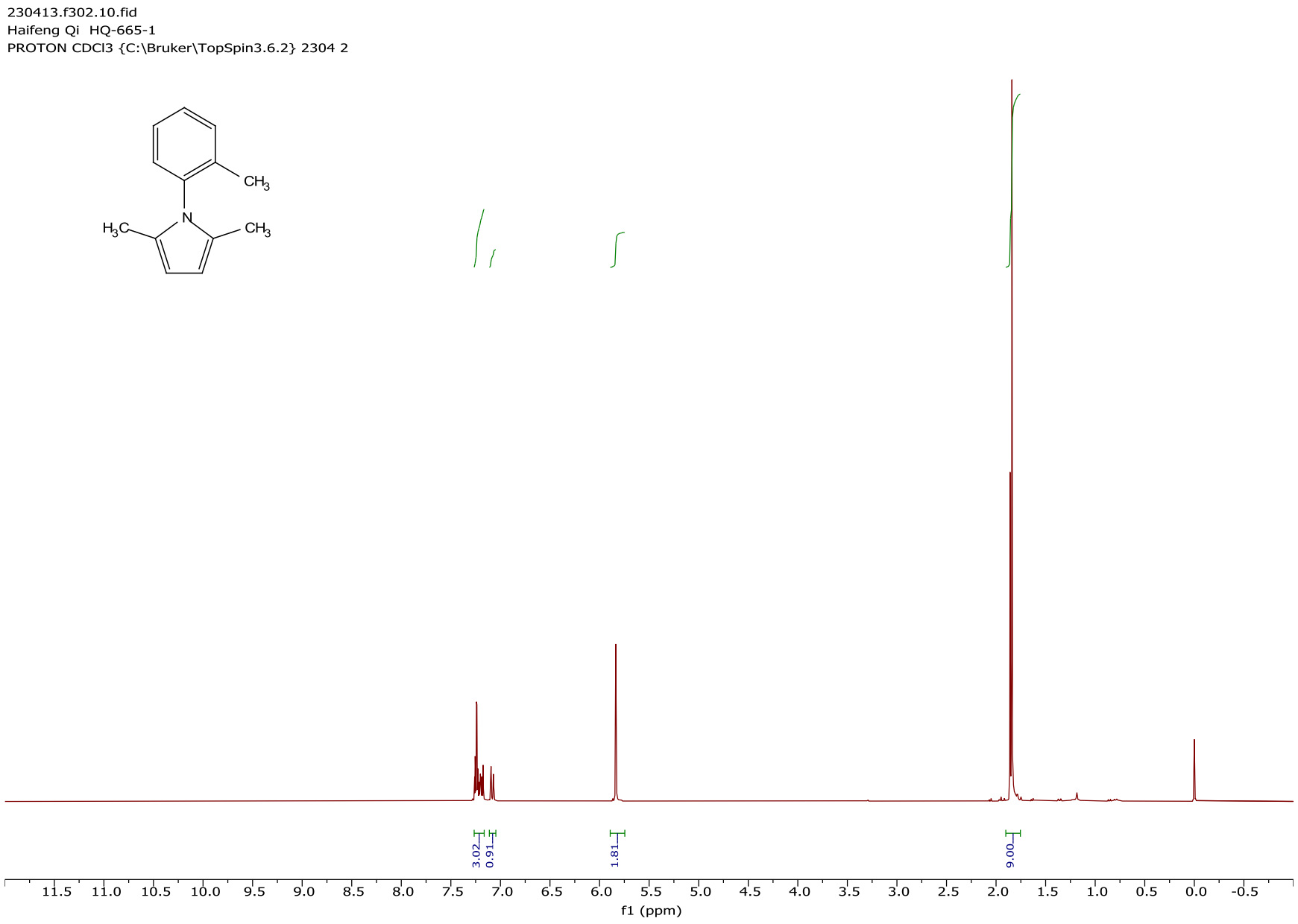

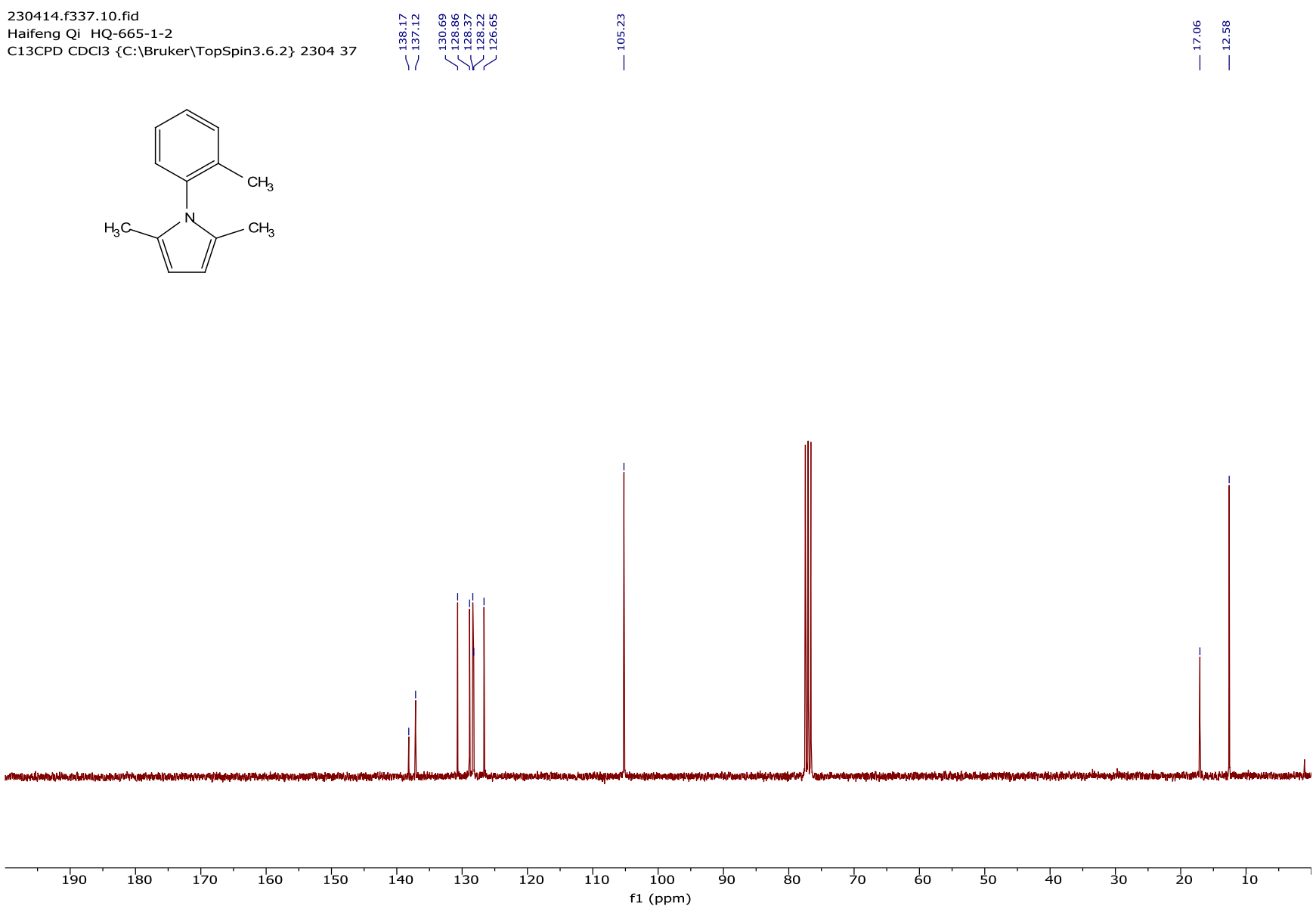

Supplementary Figure 18. ^{1}H and ^{13}C NMR spectra of product **2c**.

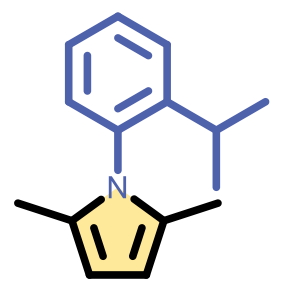

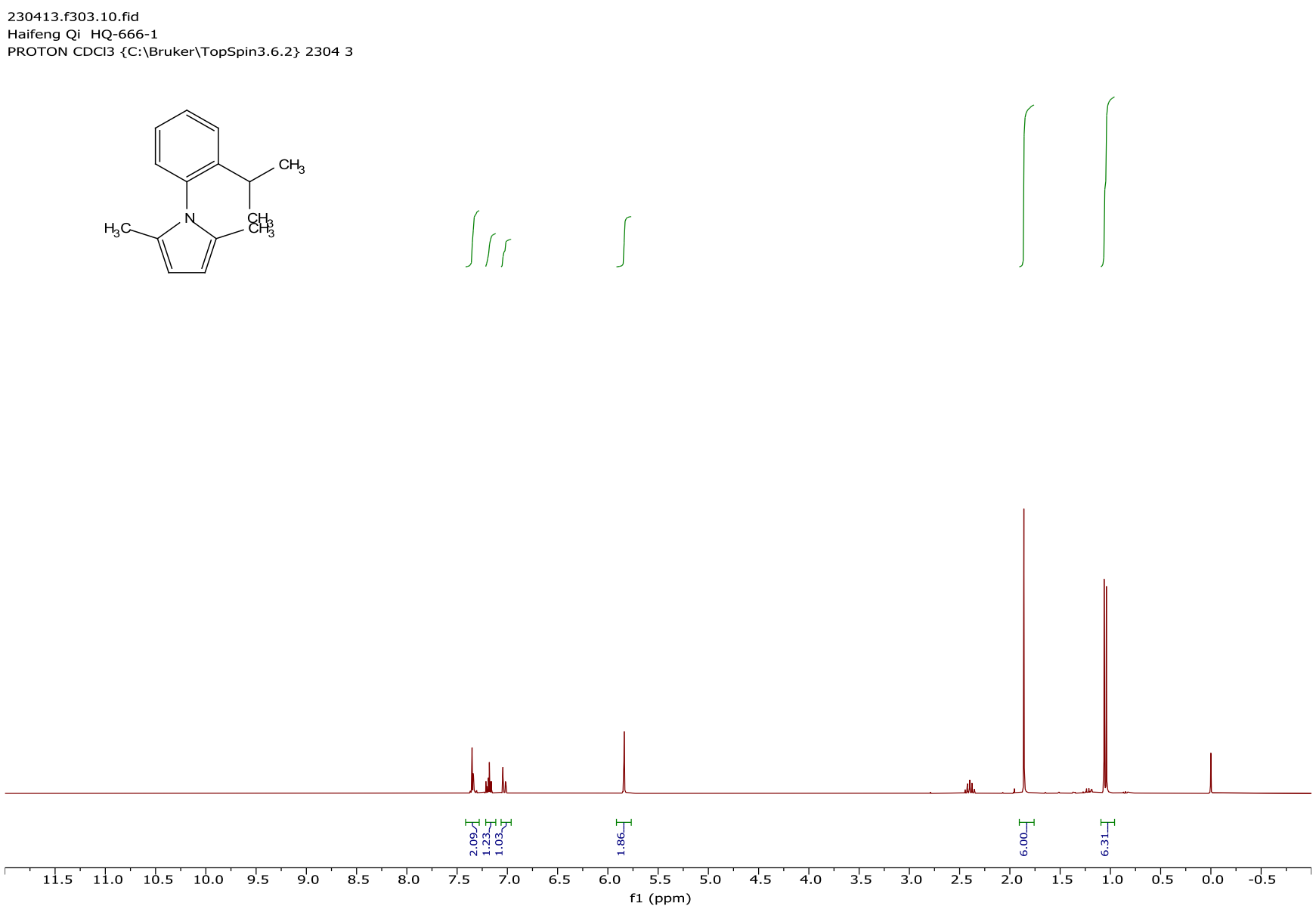

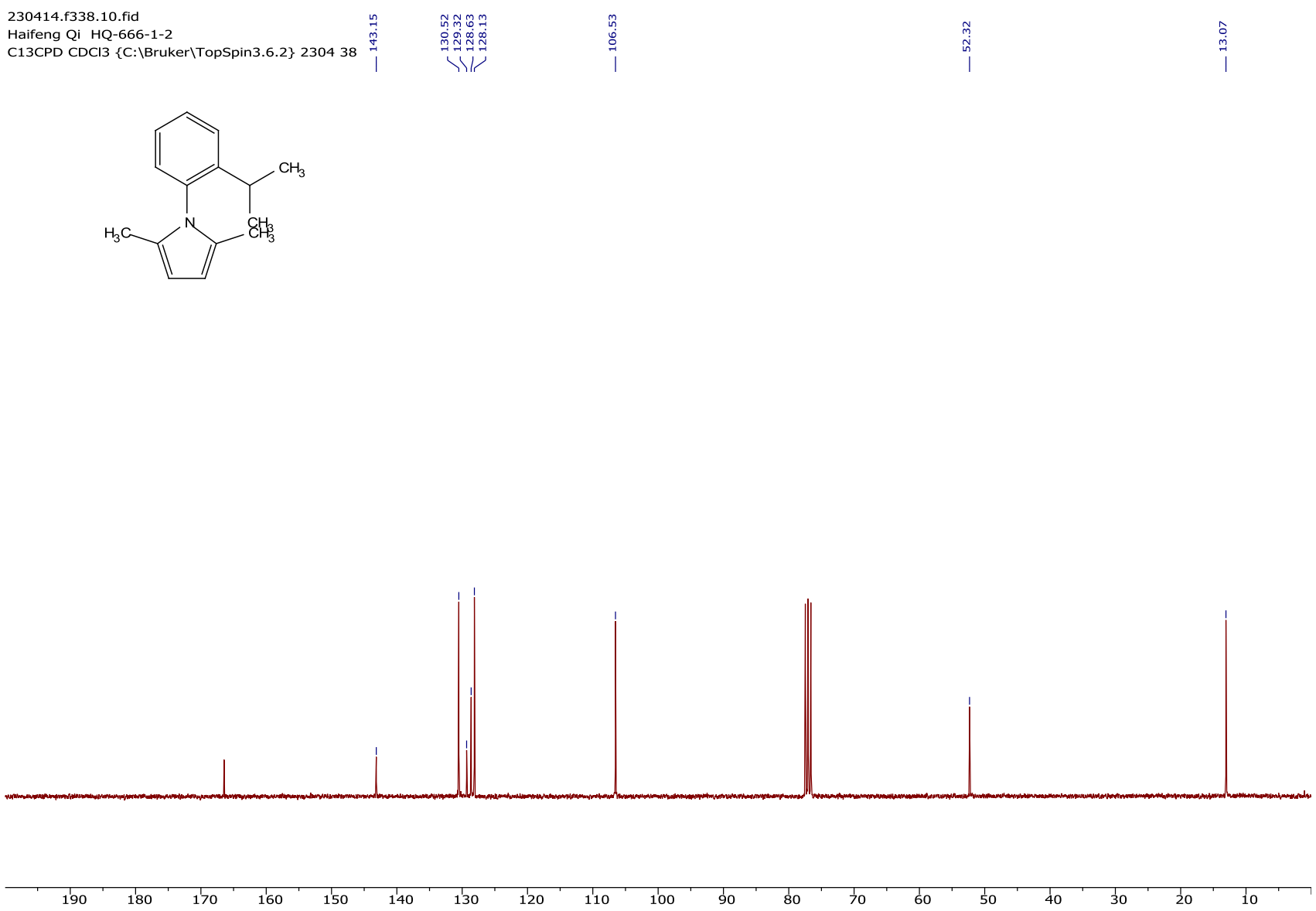

Supplementary Figure 19. ^{1}H and ^{13}C NMR spectra of product **3c**.

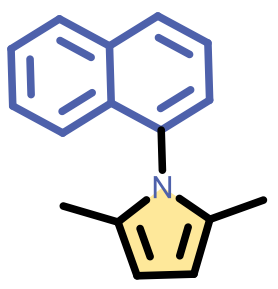

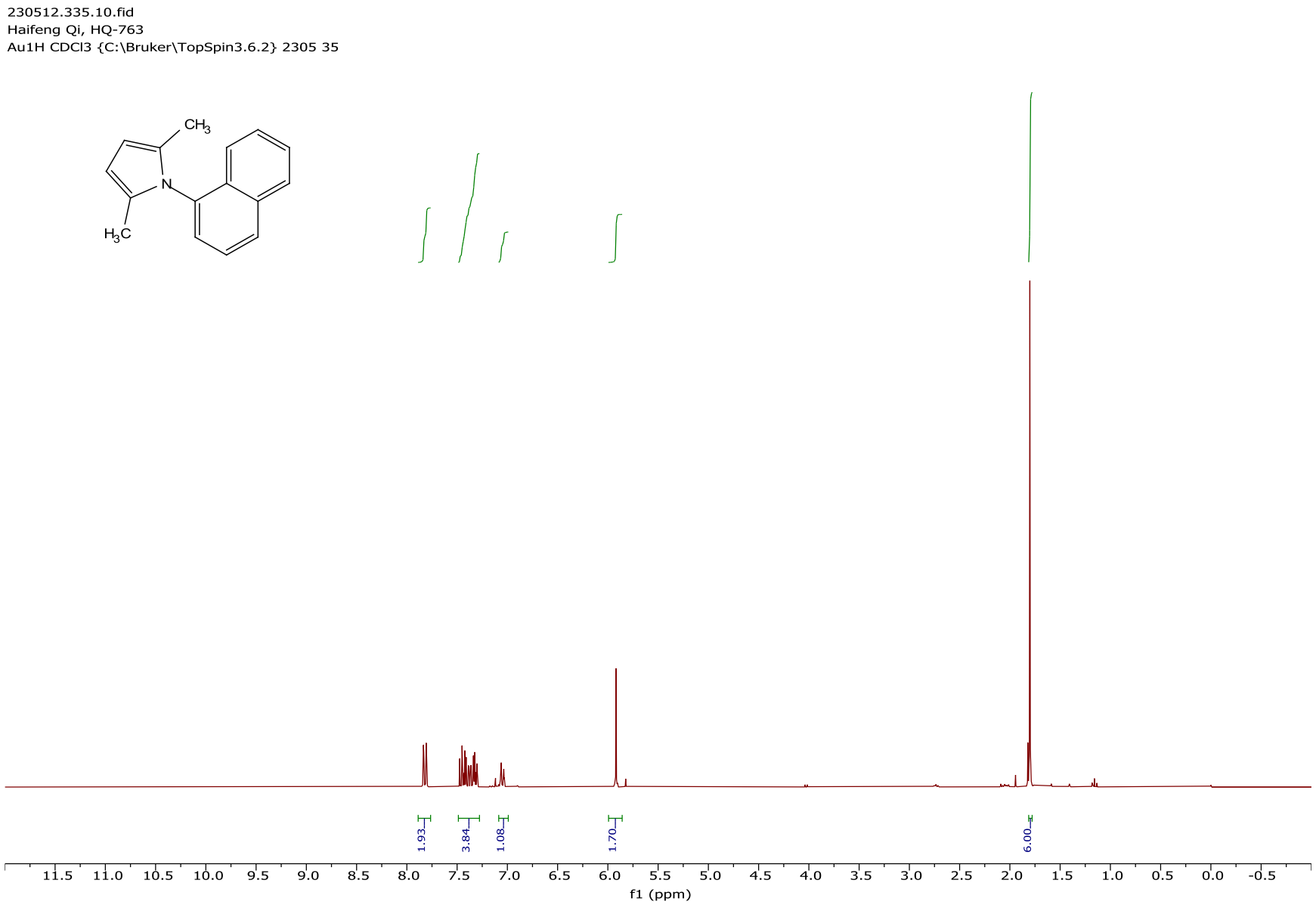

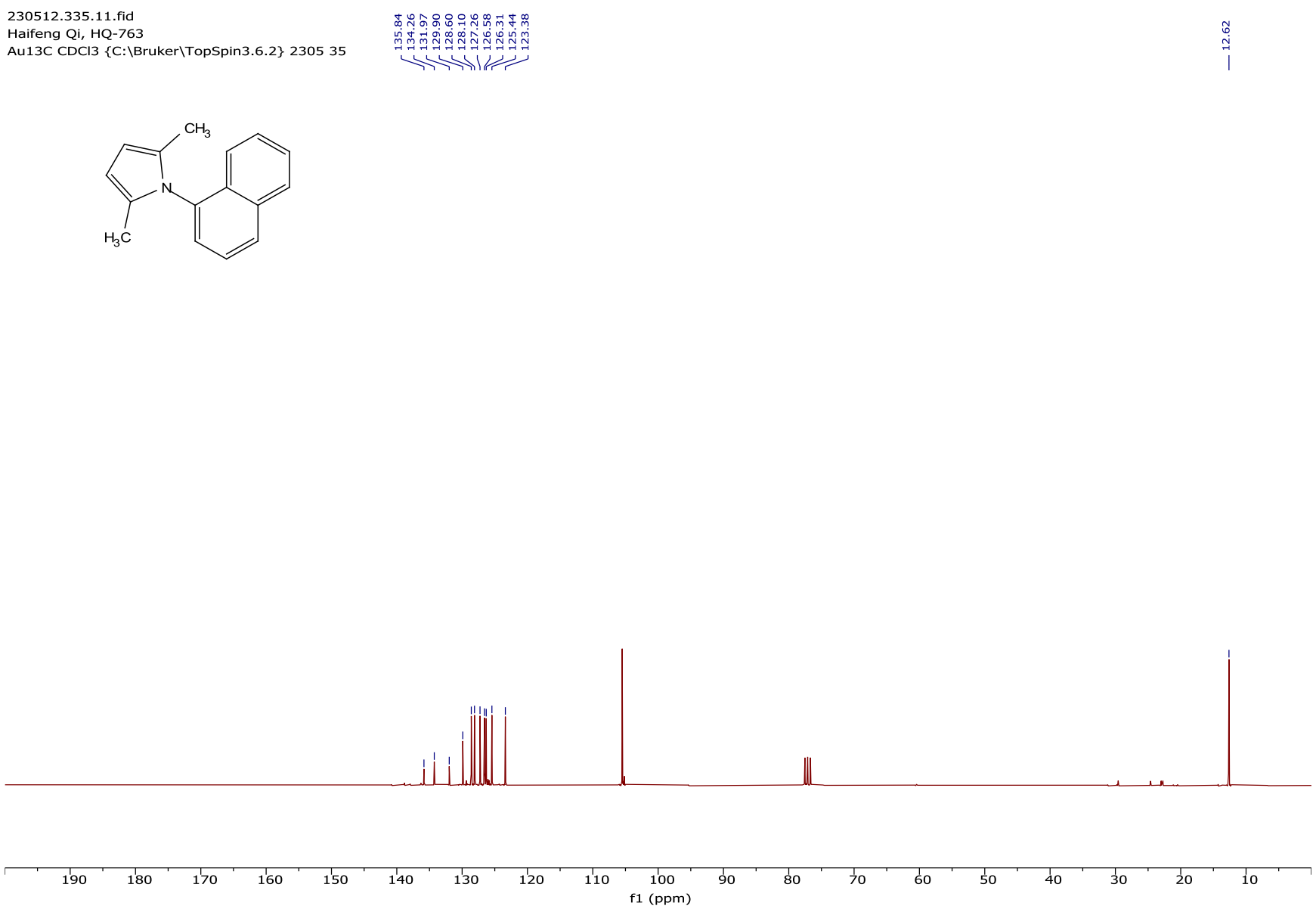

Supplementary Figure 20. ^{1}H and ^{13}C NMR spectra of product **4c**.

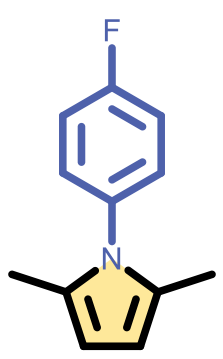
F
N

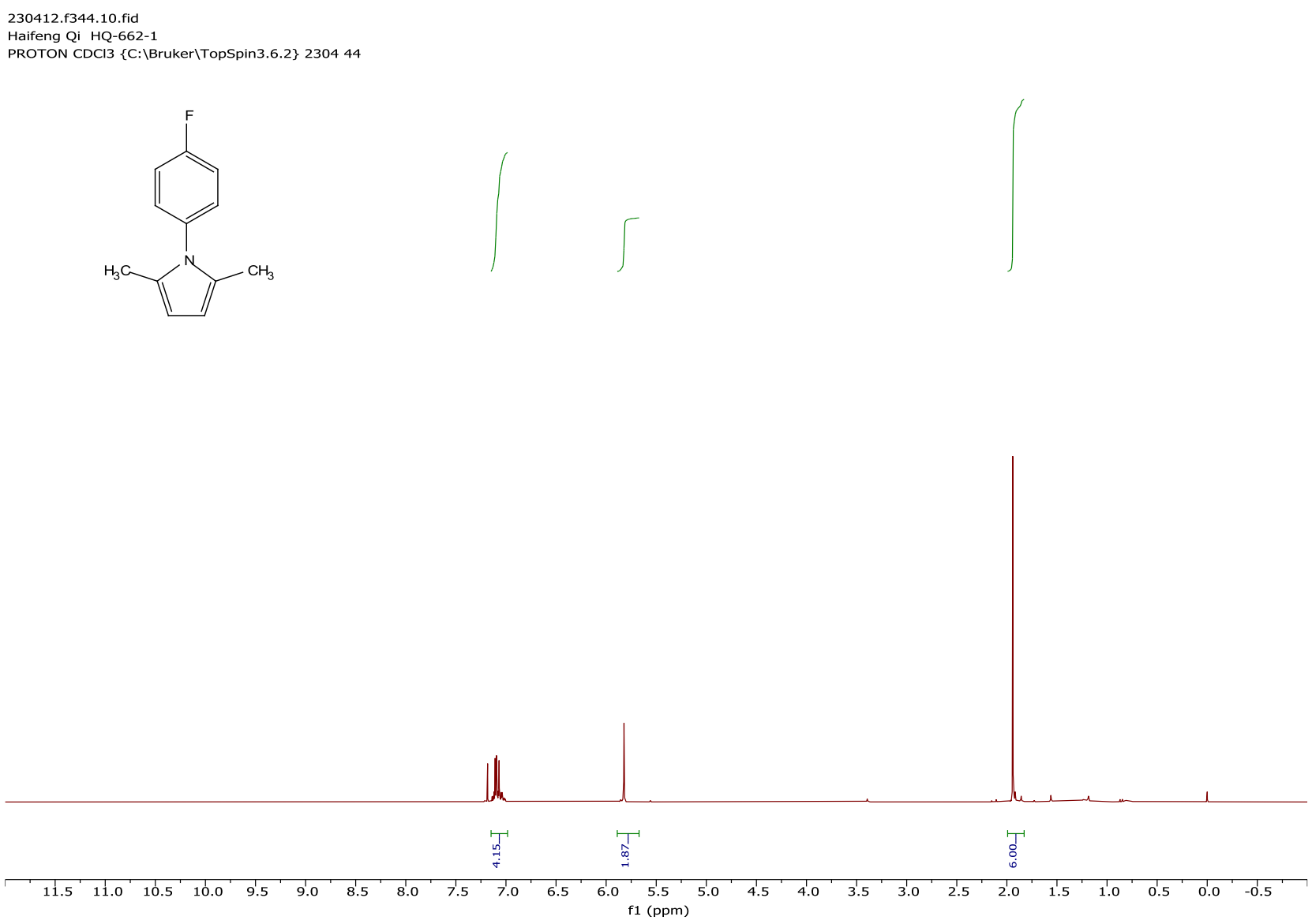
230412.f344.10.fid
Haifeng Qi HQ-662-1
PROTON CDCl3 {C:\Bruker\TopSpin3.6.2} 2304 44
F
N
H3C
CH3
4.15
1.87
6.00
11.5 11.0 10.5 10.0 9.5 9.0 8.5 8.0 7.5 7.0 6.5 6.0 5.5 5.0 4.5 4.0 3.5 3.0 2.5 2.0 1.5 1.0 0.5 0.0 -0.5
f1 (ppm)

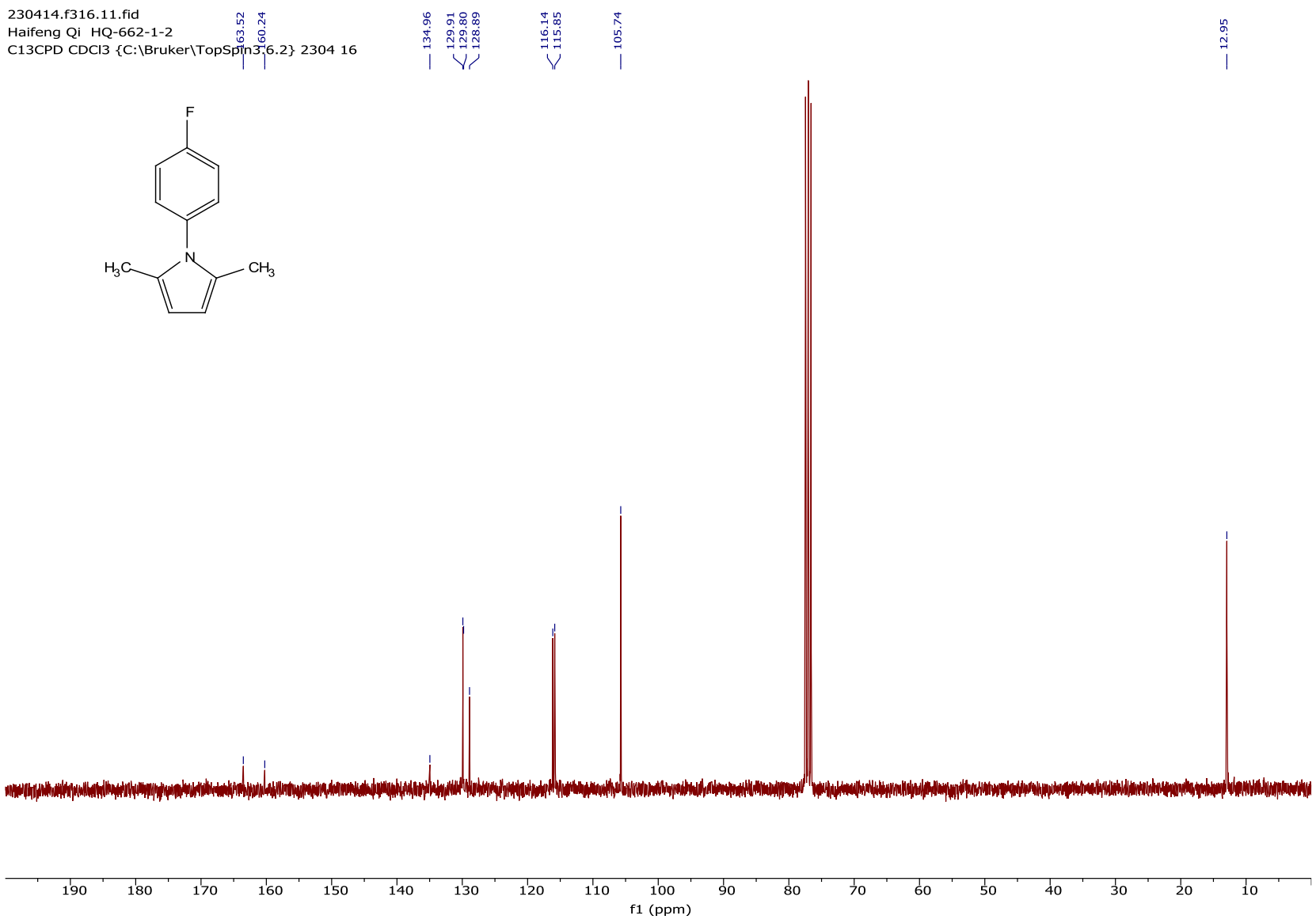
230414.f316.11.fid
Haifeng Qi HQ-662-1-2
C13CPD CDCl3 {C:\Bruker\TopSpin3.6.2} 2304 16
163.52
160.24
134.96
129.91
129.80
128.89
116.14
115.85
105.74
12.95
F
N
H3C
CH3
190 180 170 160 150 140 130 120 110 100 90 80 70 60 50 40 30 20 10
f1 (ppm)

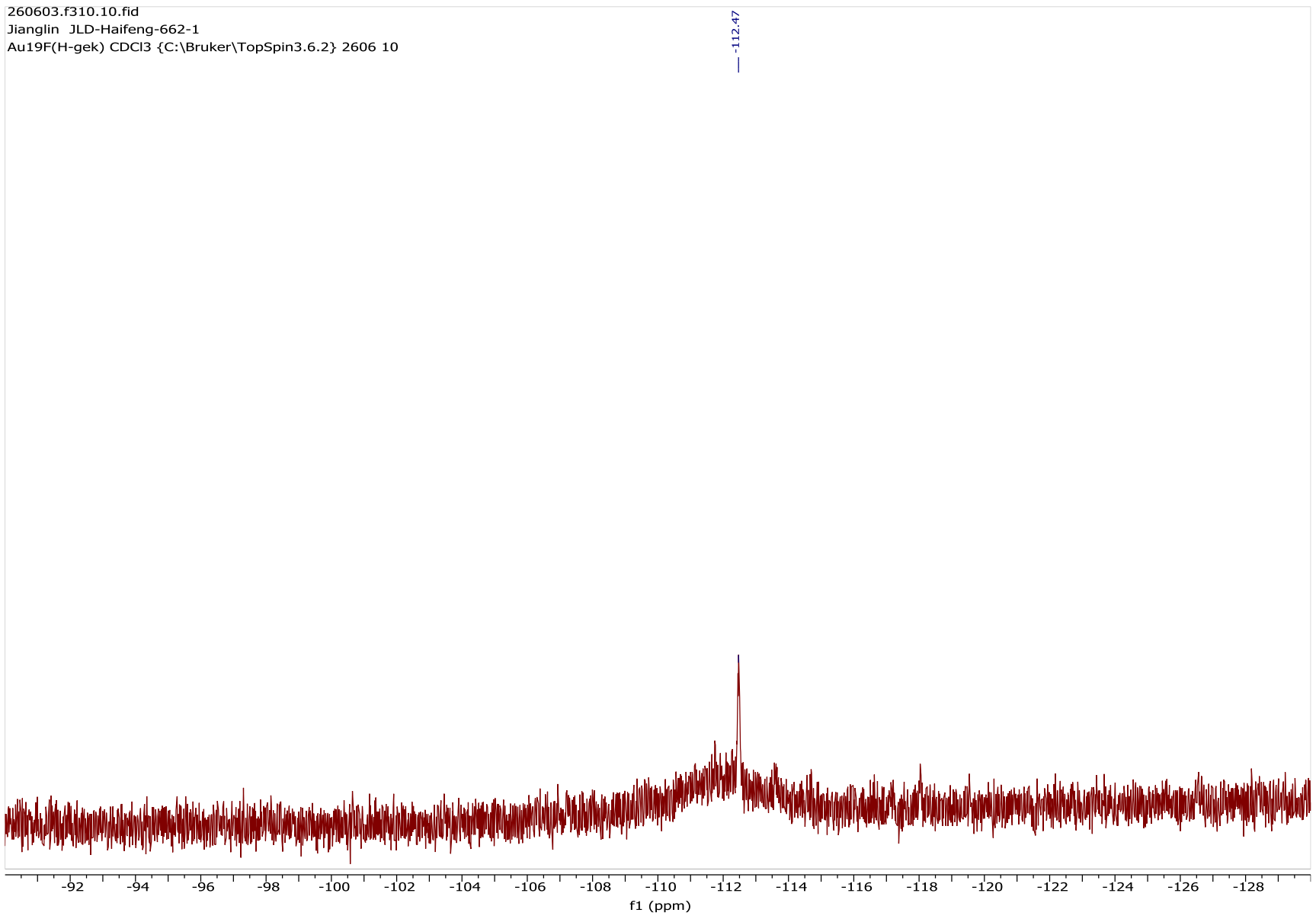

Supplementary Figure 21. ^{1}H, ^{13}C and ^{19}F NMR spectra of product **5c**.

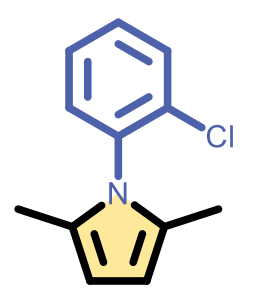

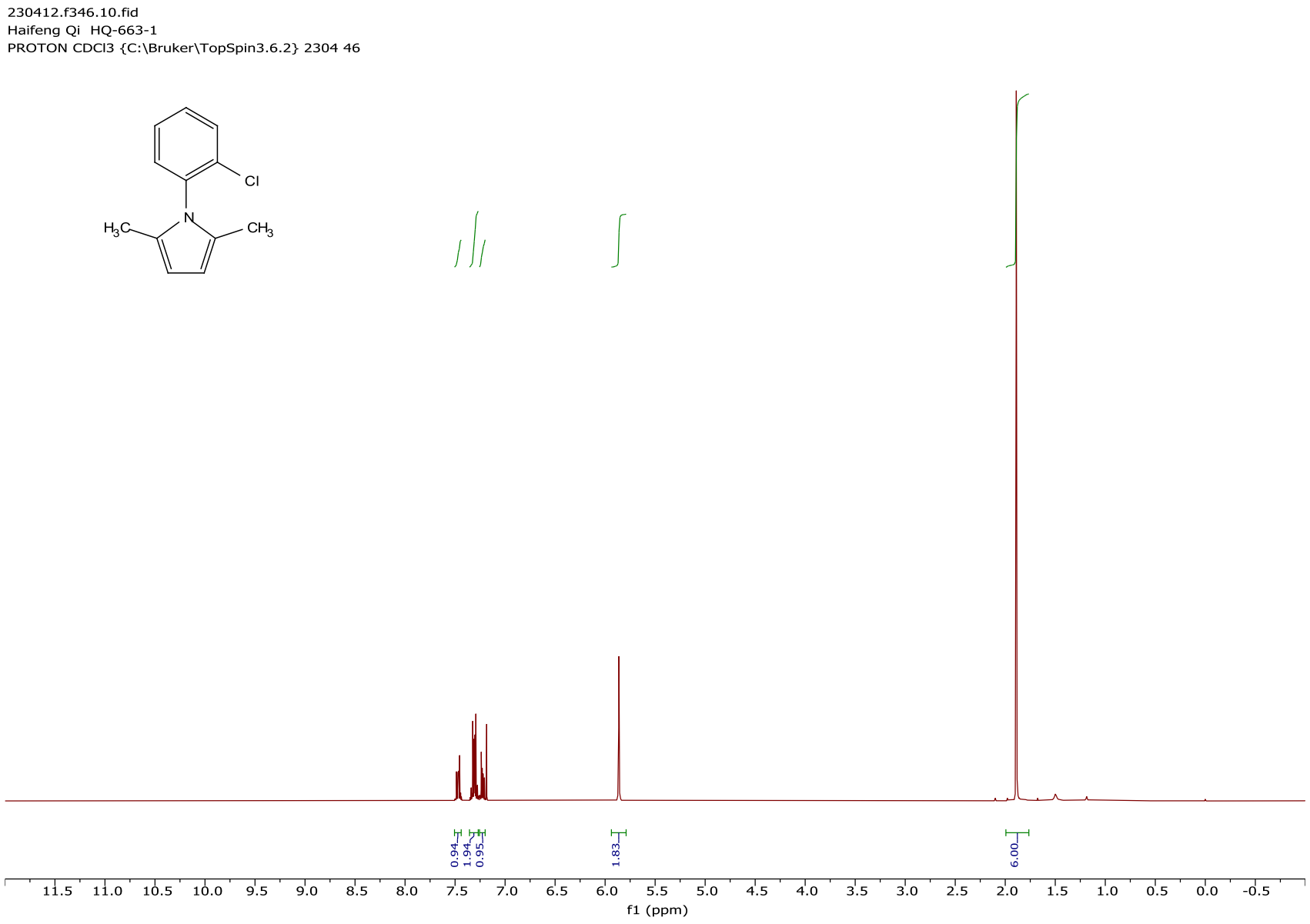

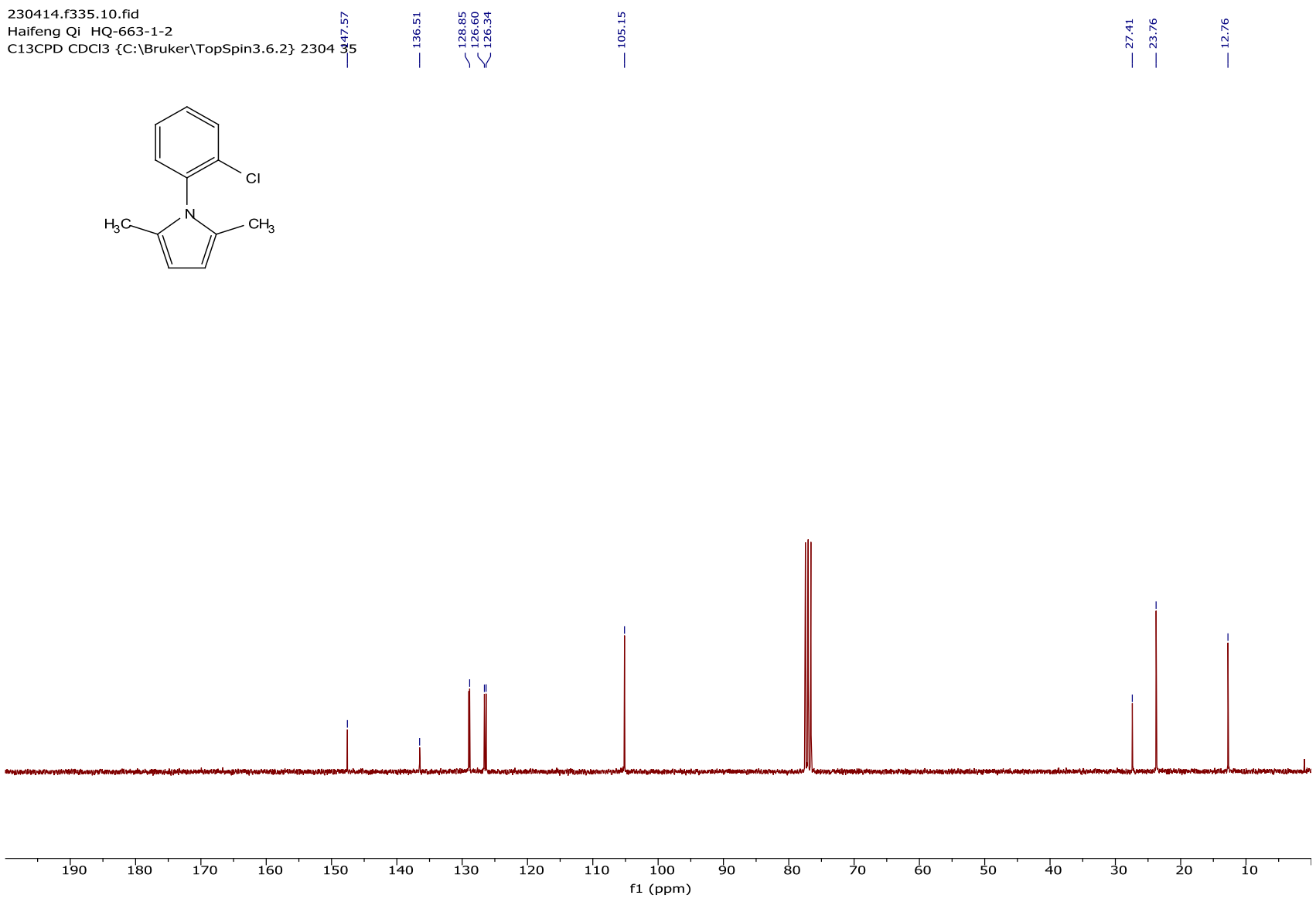

Supplementary Figure 22. ^{1}H and ^{13}C NMR spectra of product **6c**.

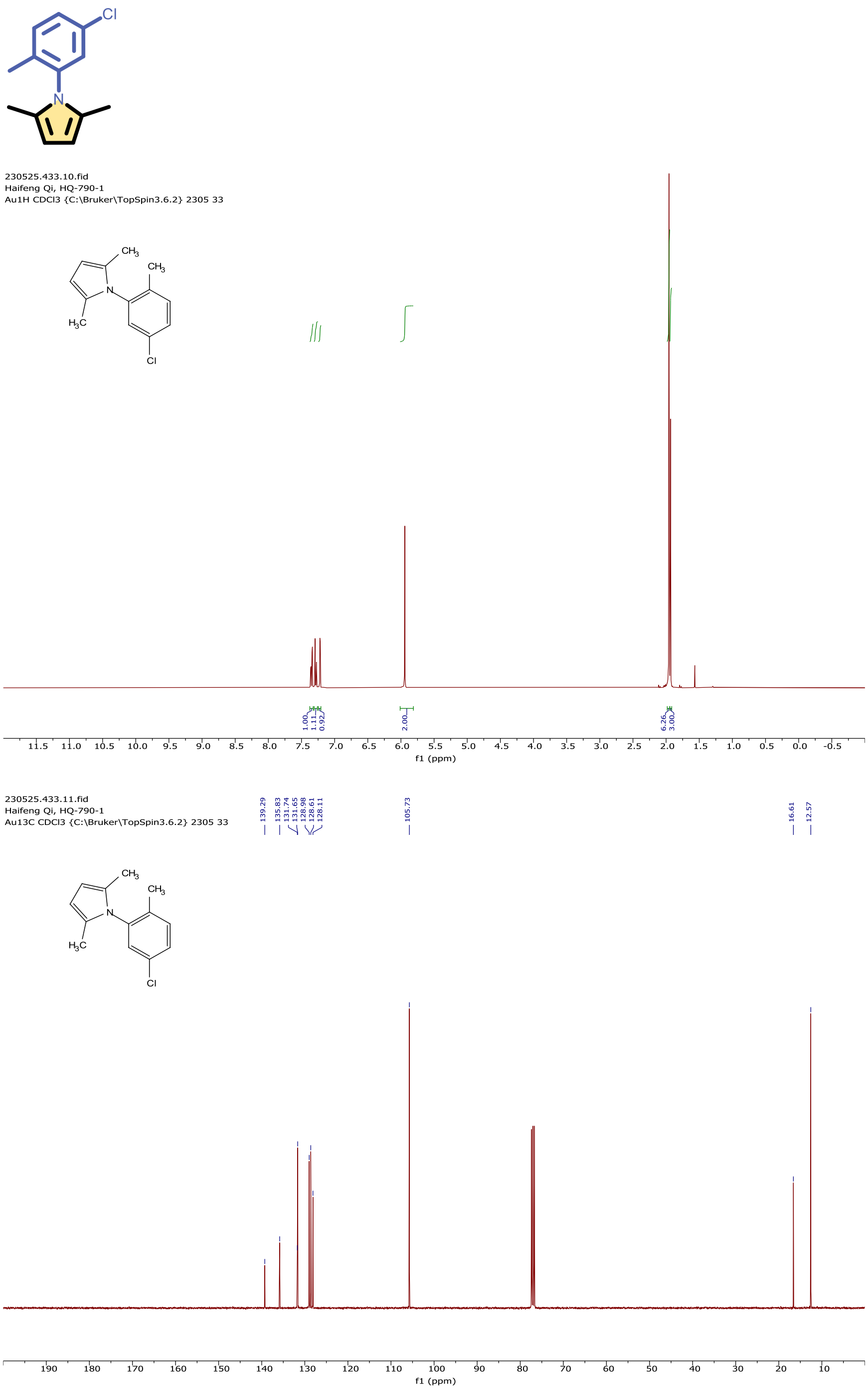

Supplementary Figure 23. ^{1}H and ^{13}C NMR spectra of product **7c**.

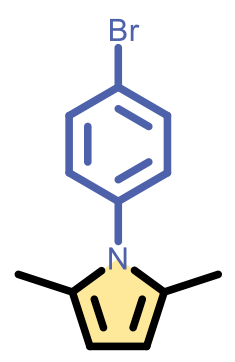

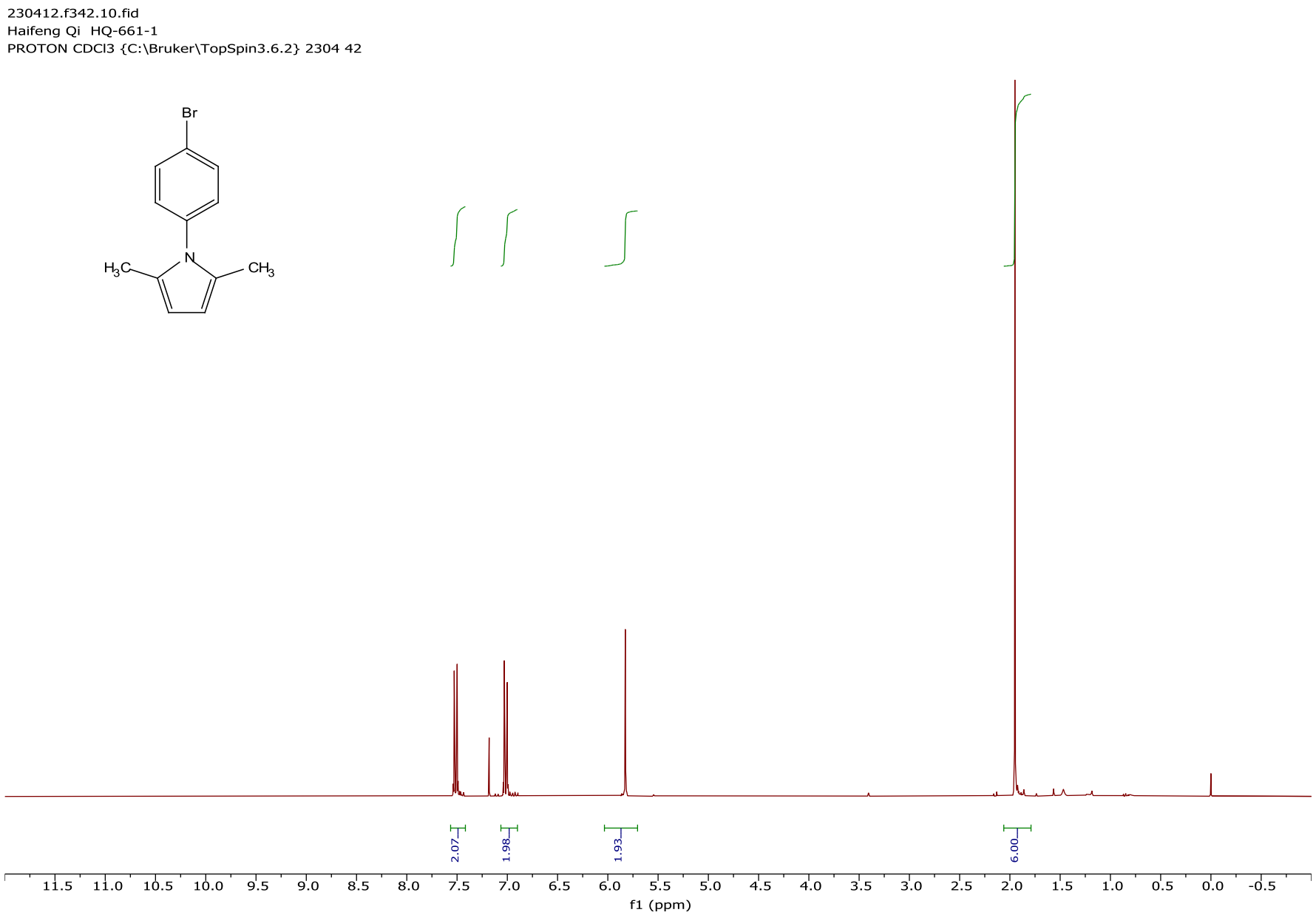

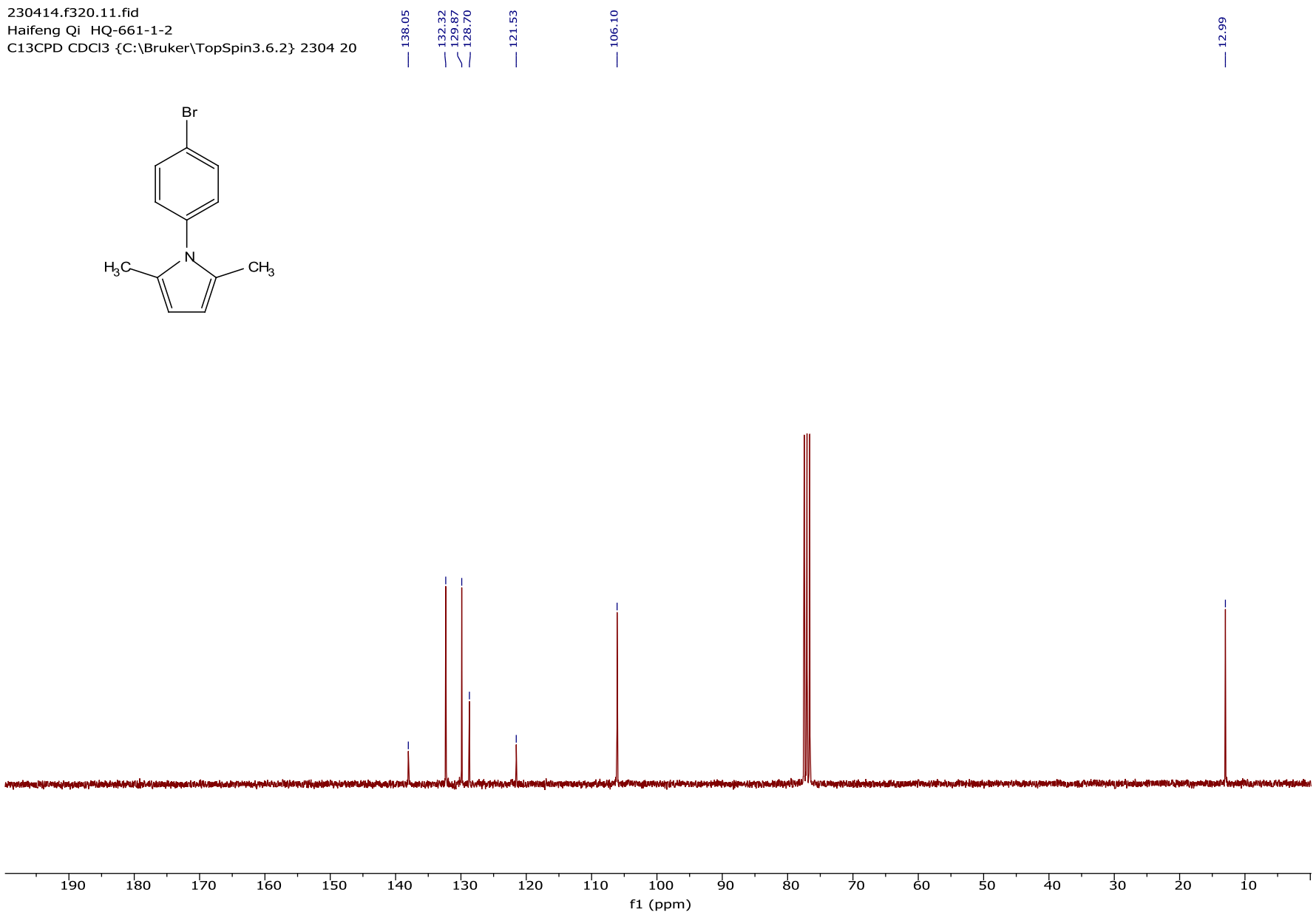

Supplementary Figure 24. ^{1}H and ^{13}C NMR spectra of product **8c**.

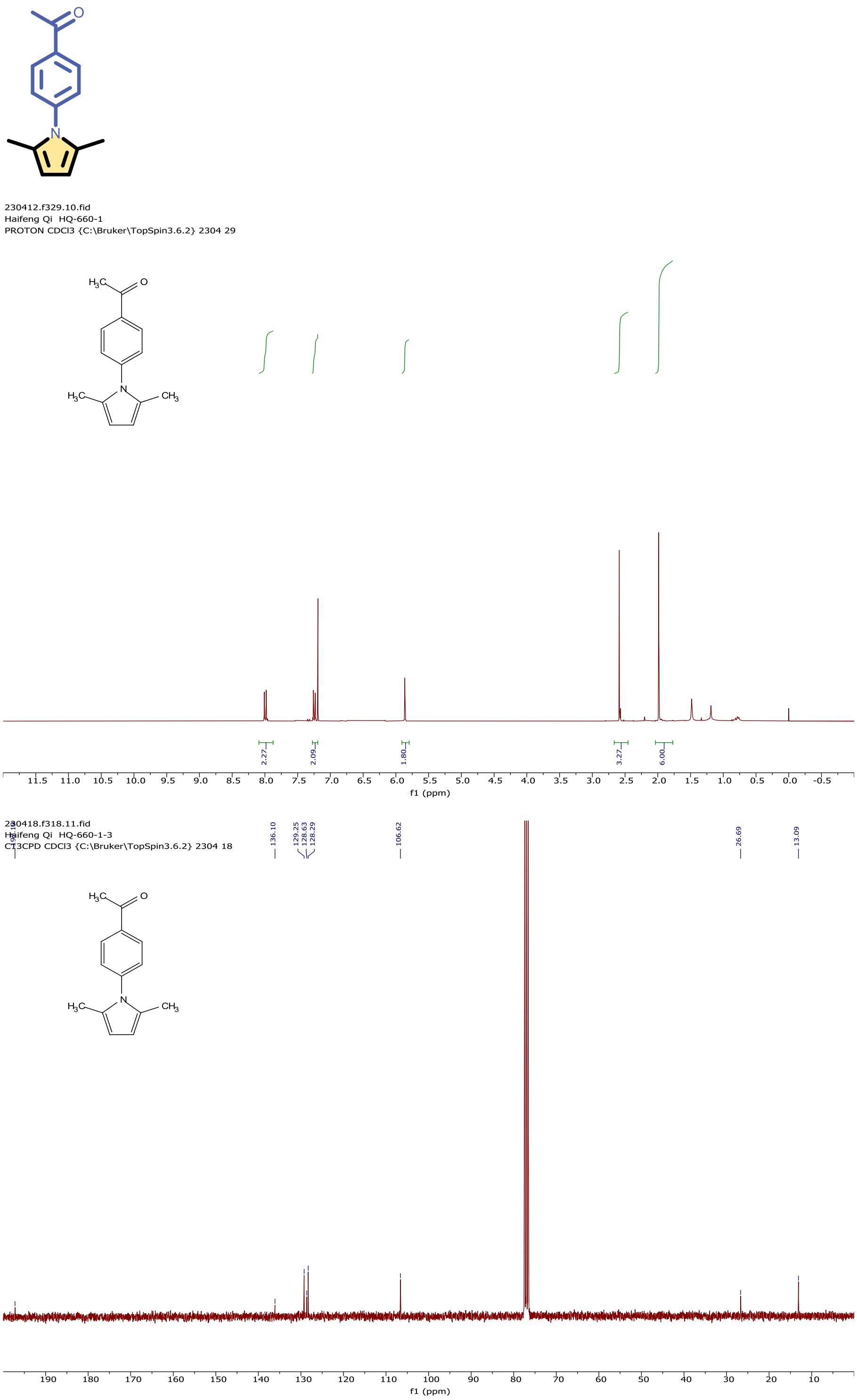

Supplementary Figure 25. ^{1}H and ^{13}C NMR spectra of product **9c**.

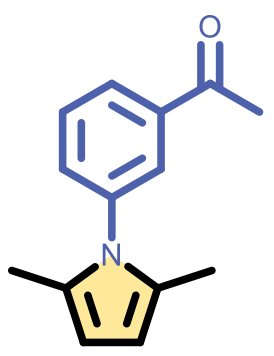

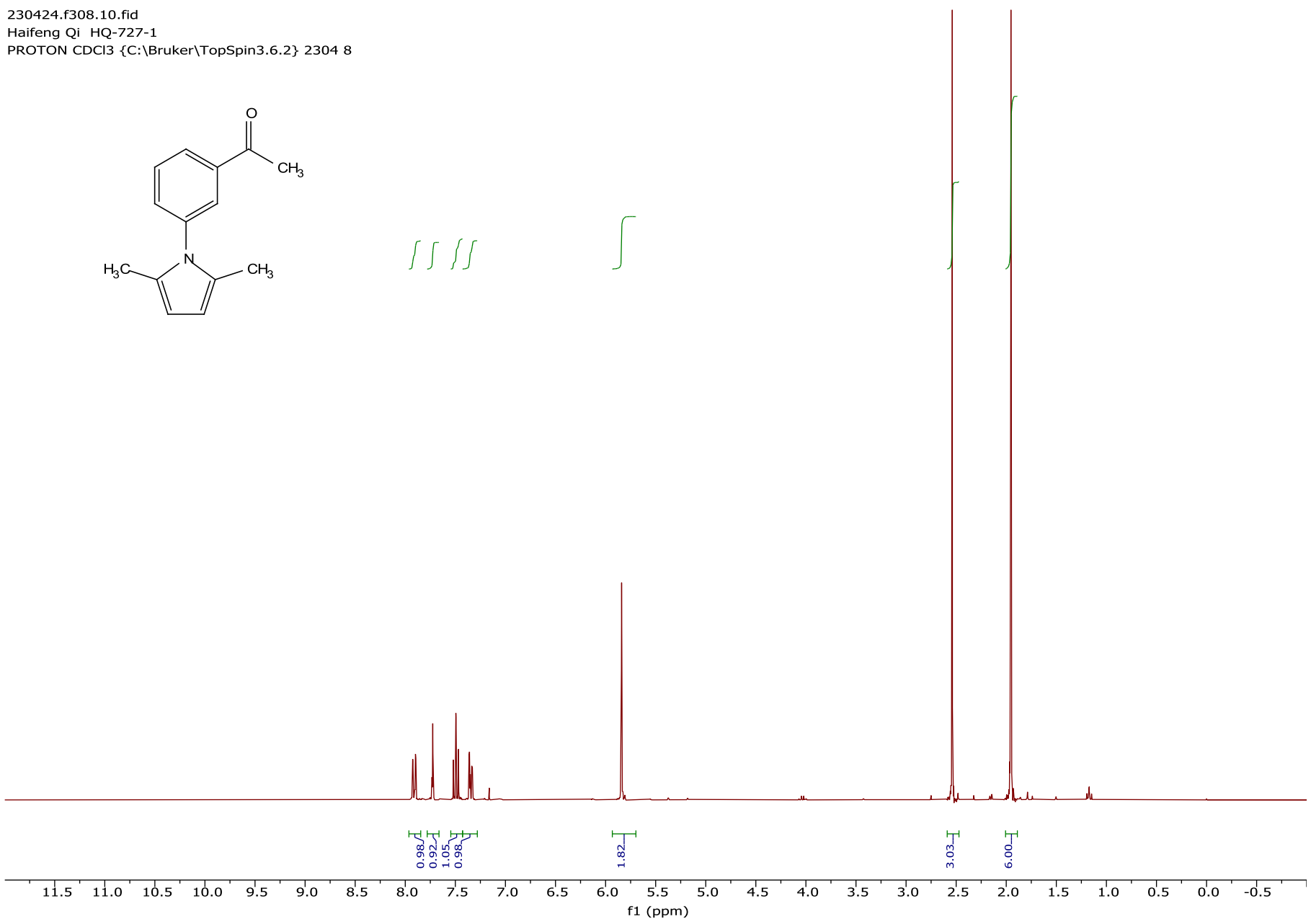

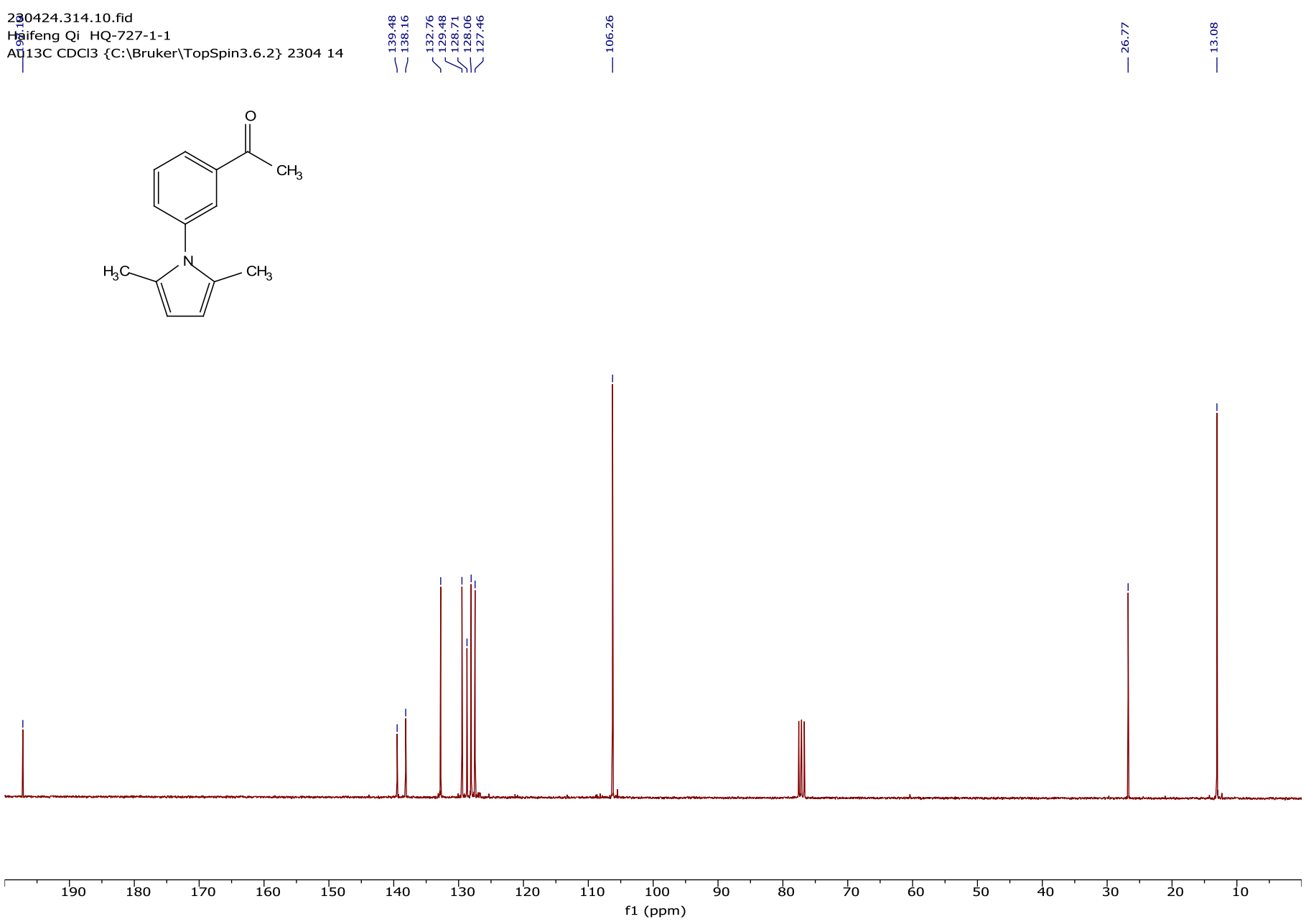

Supplementary Figure 26. ^{1}H and ^{13}C NMR spectra of product **10c**.

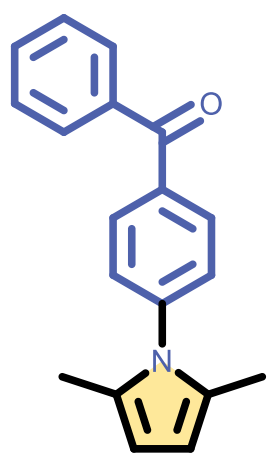

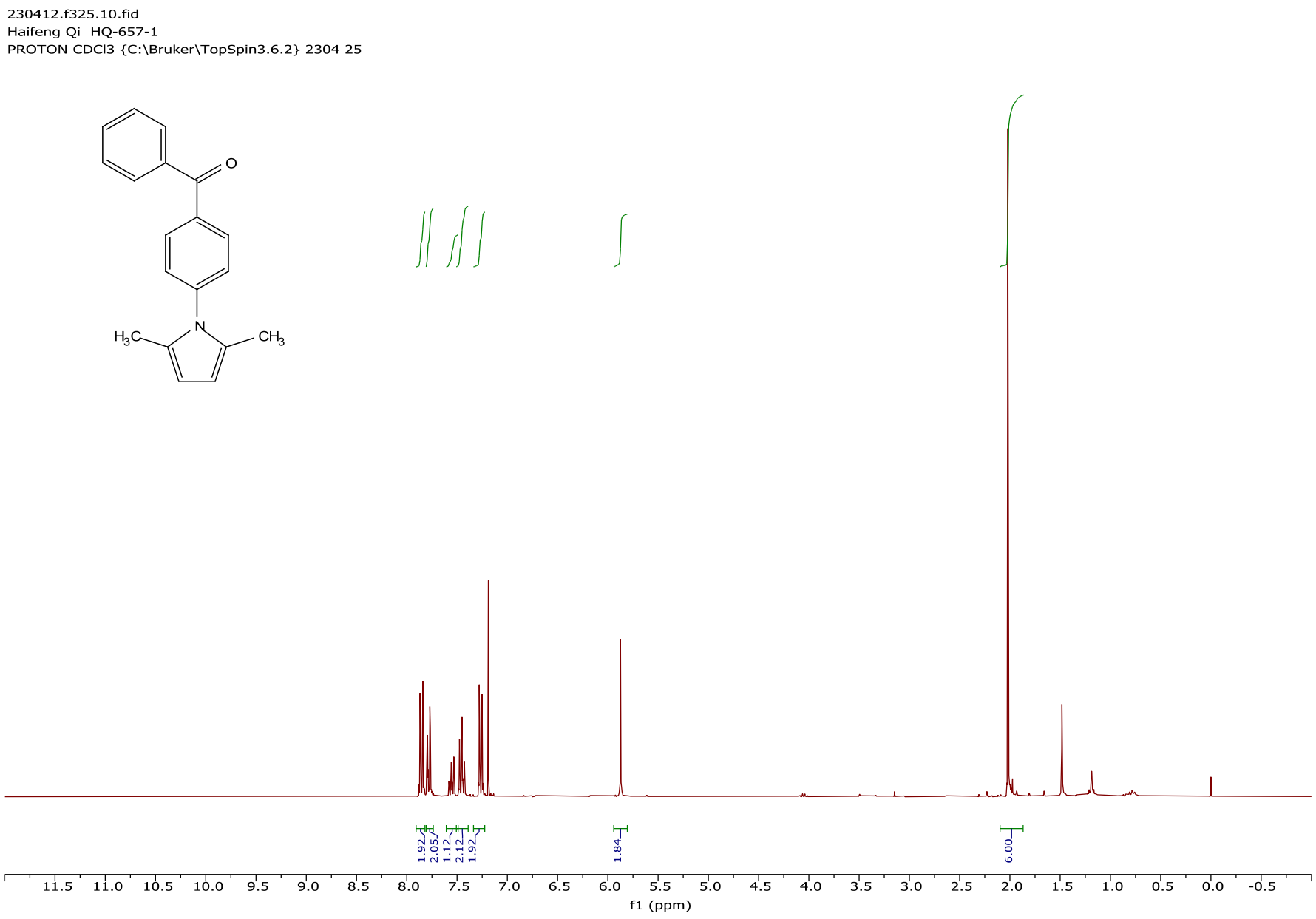

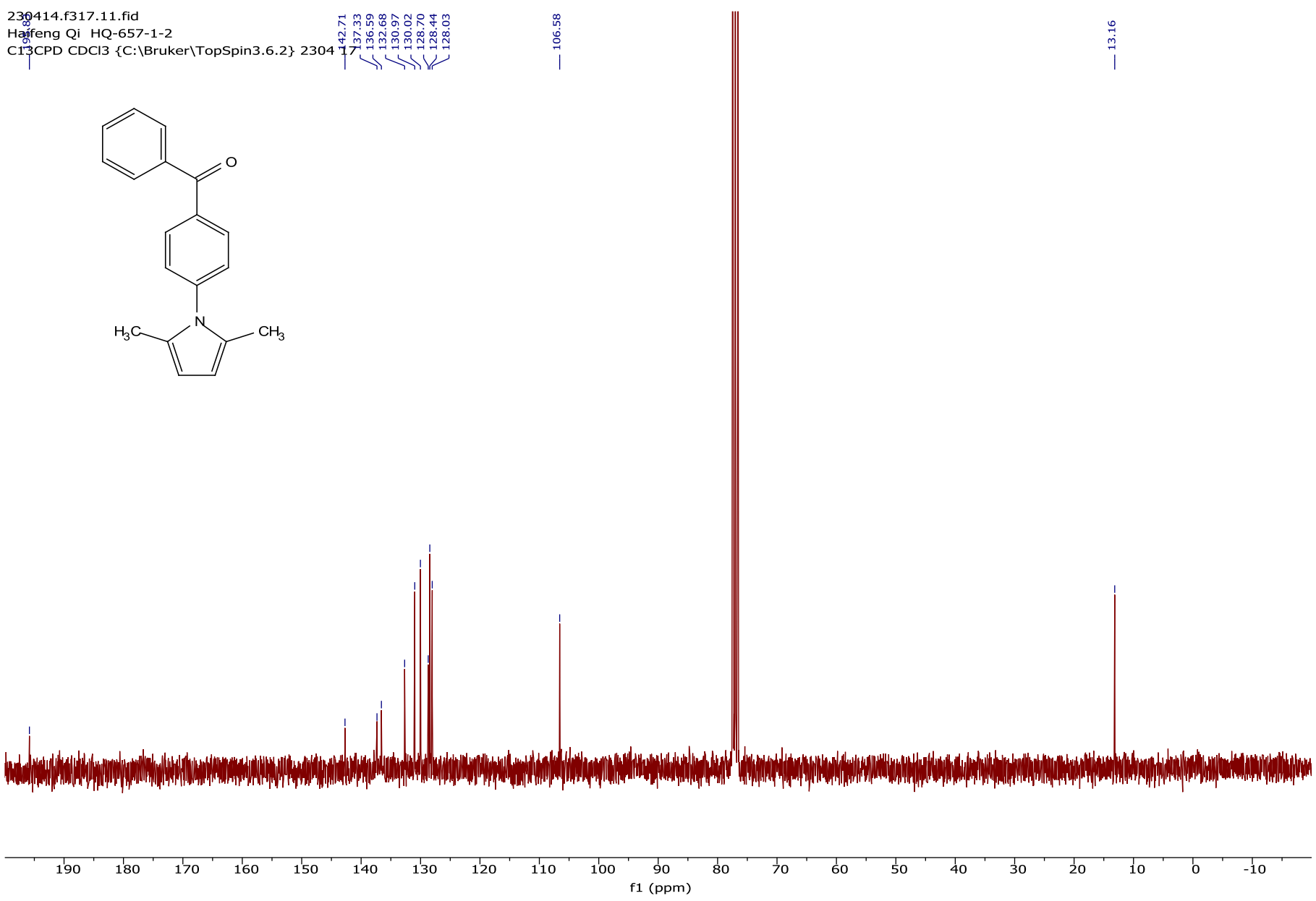

Supplementary Figure 27. ^{1}H and ^{13}C NMR spectra of product **11c**.

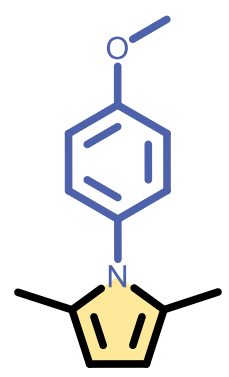

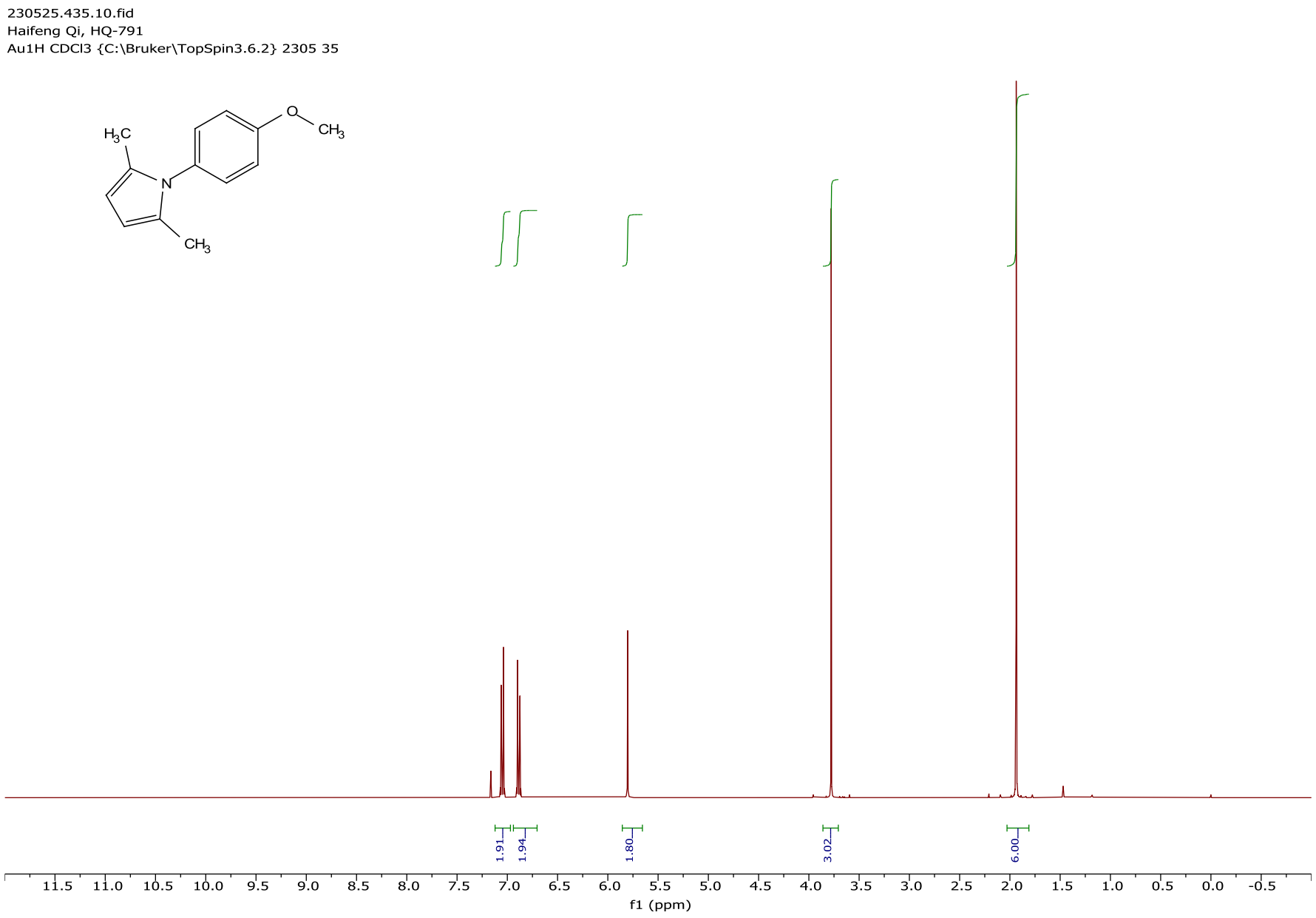

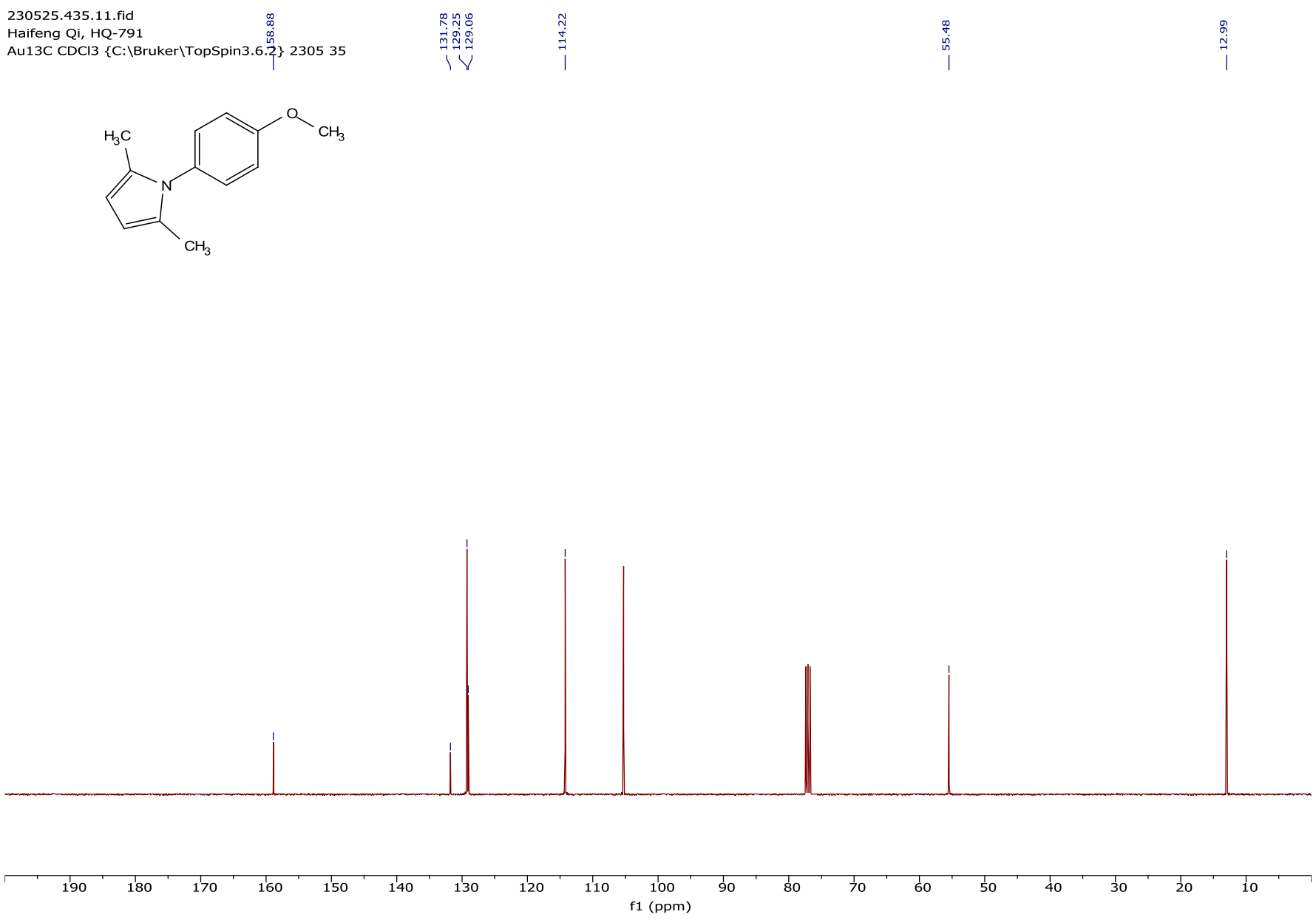

Supplementary Figure 28. ^{1}H and ^{13}C NMR spectra of product **12c**.

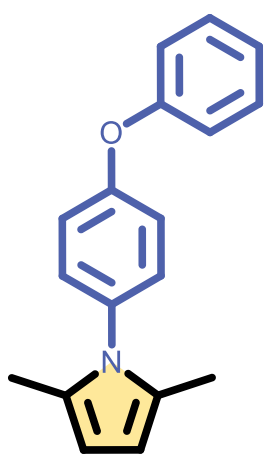

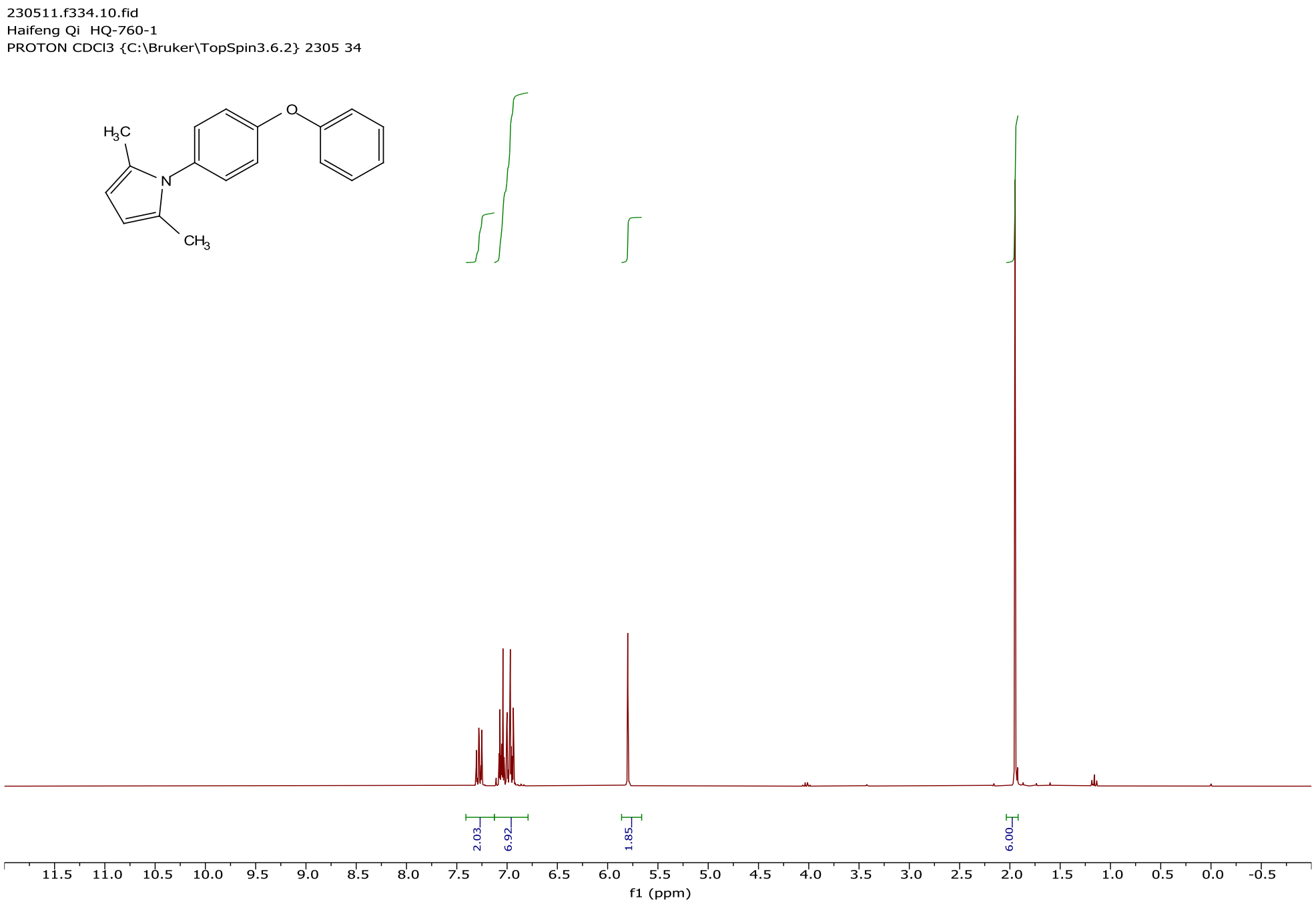

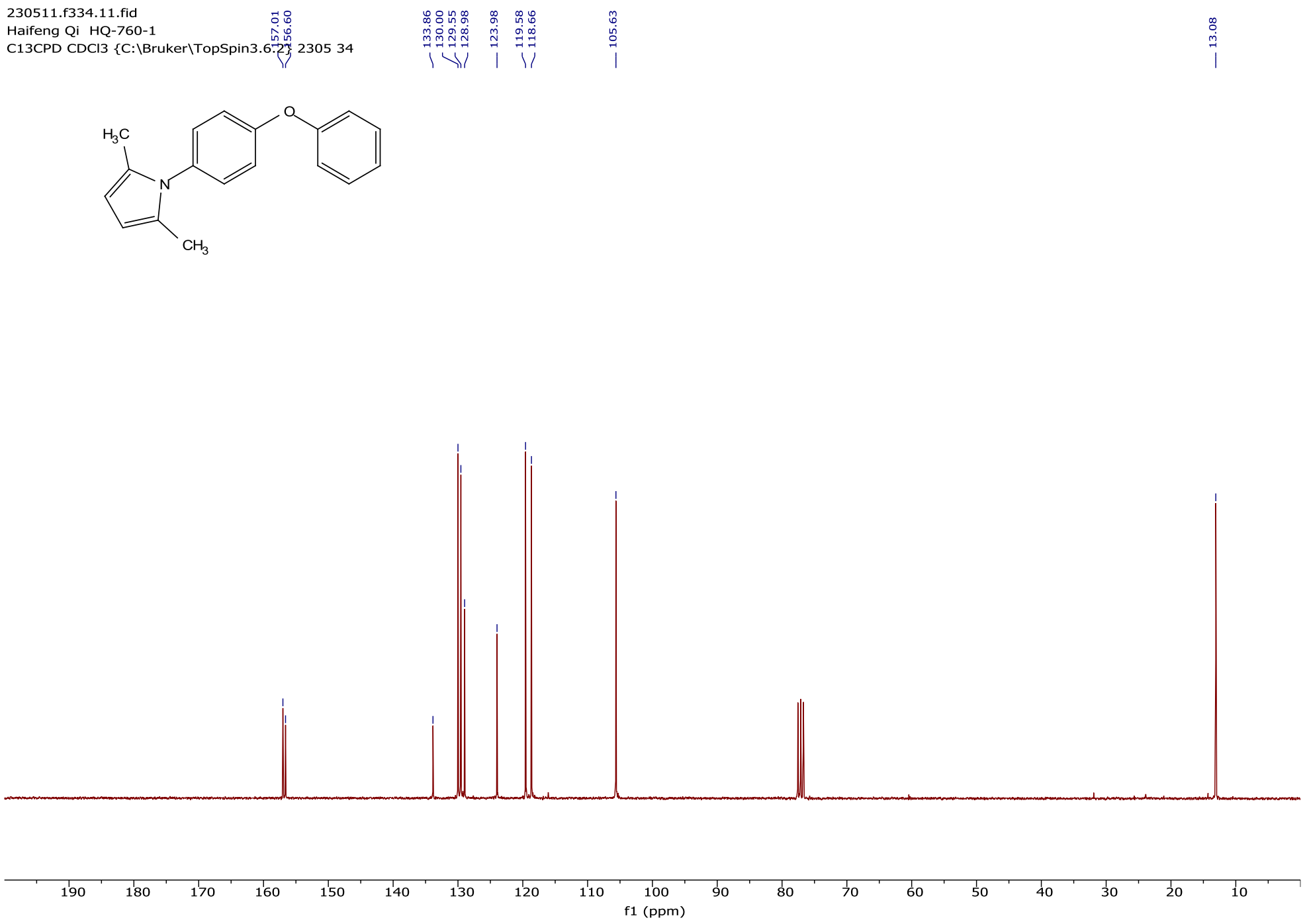

Supplementary Figure 29. ^{1}H and ^{13}C NMR spectra of product **13c**.

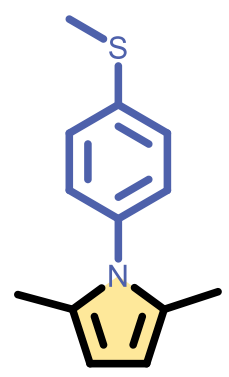

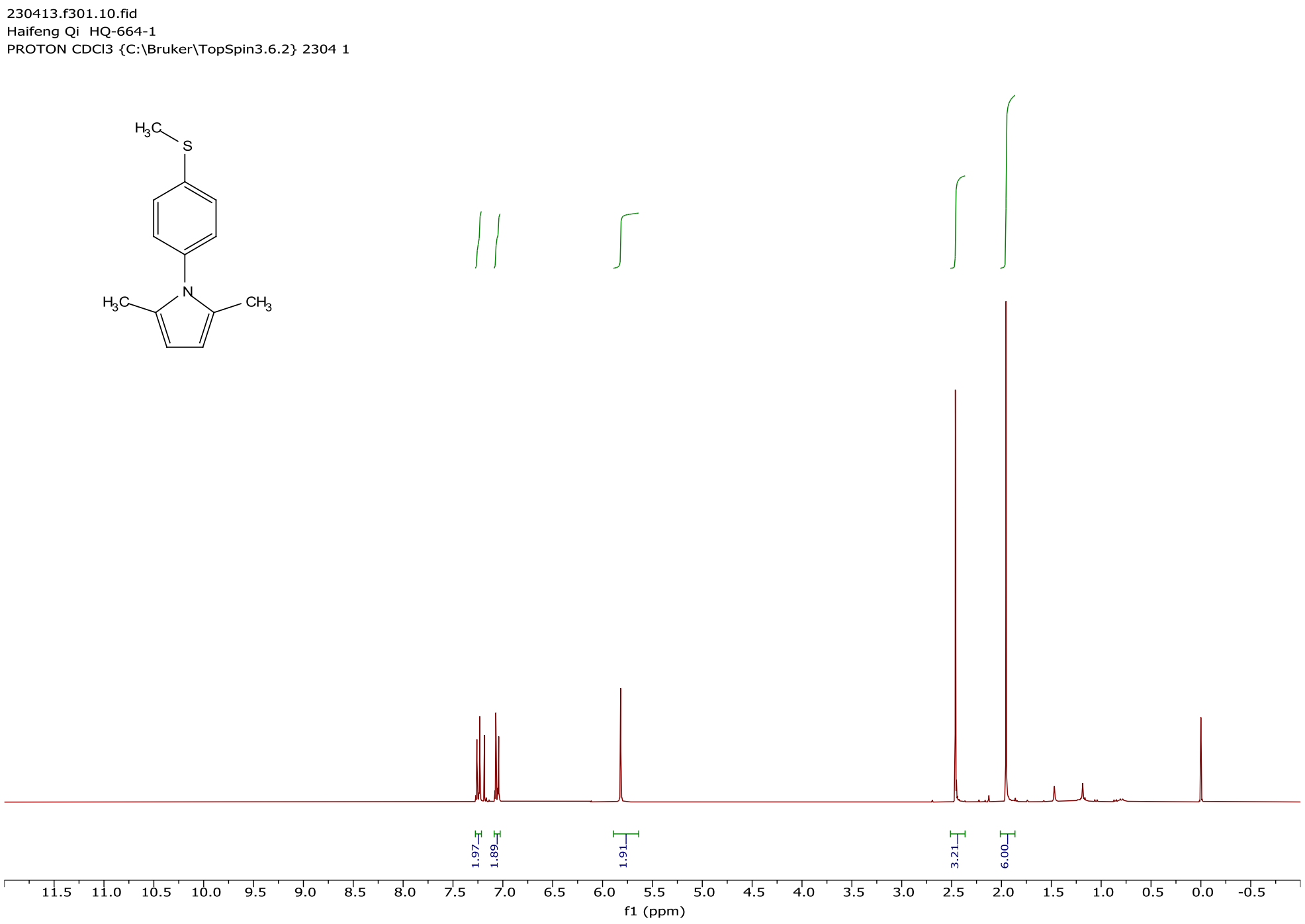

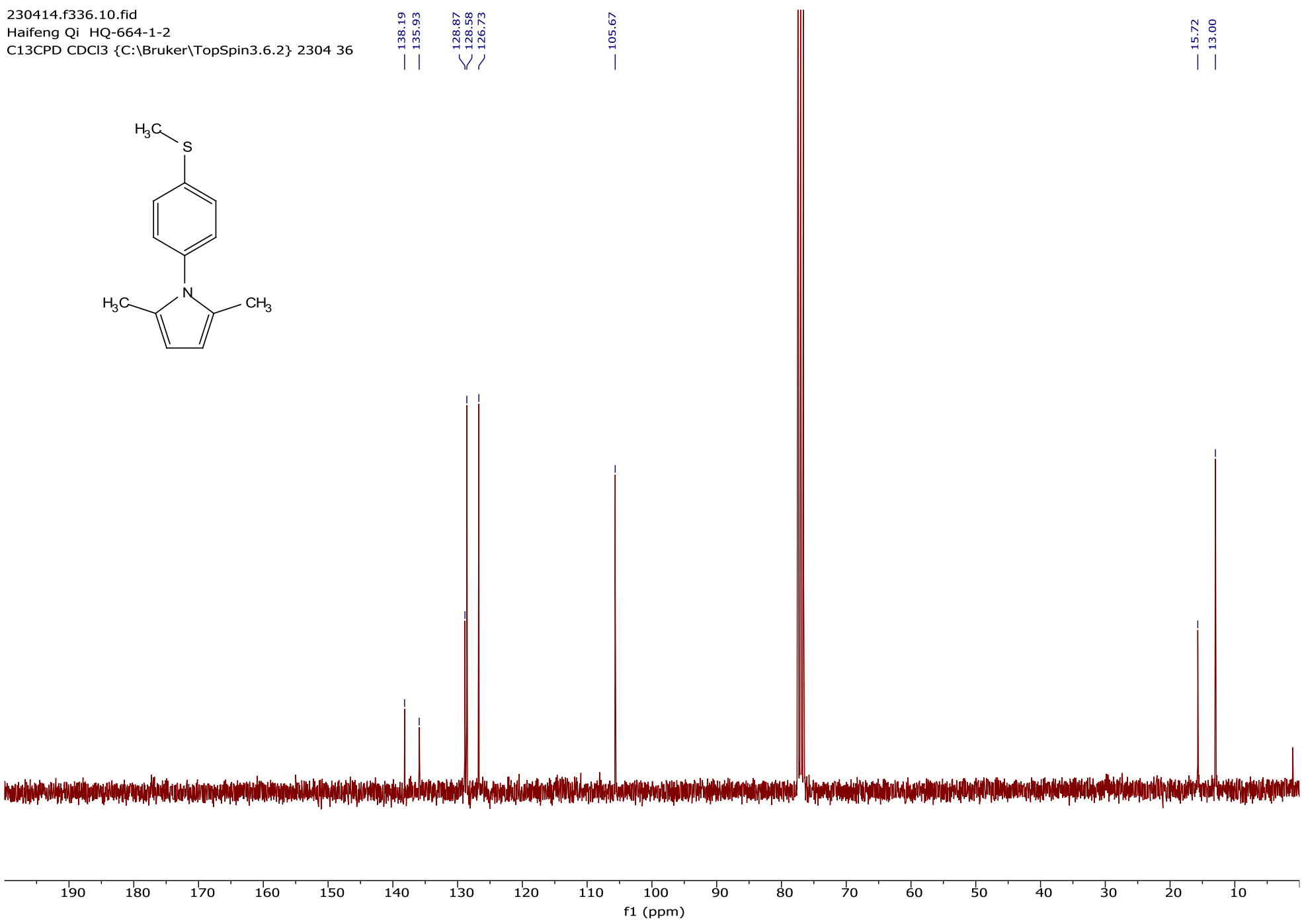

Supplementary Figure 30. ^{1}H and ^{13}C NMR spectra of product **14c**.

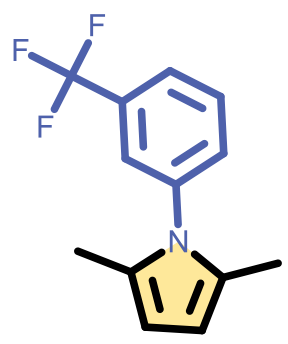

230418.f339.10.fid
Haifeng Qi HQ-715-1
PROTON CDCl3 {C:\Bruker\TopSpin3.6.2} 2304 39

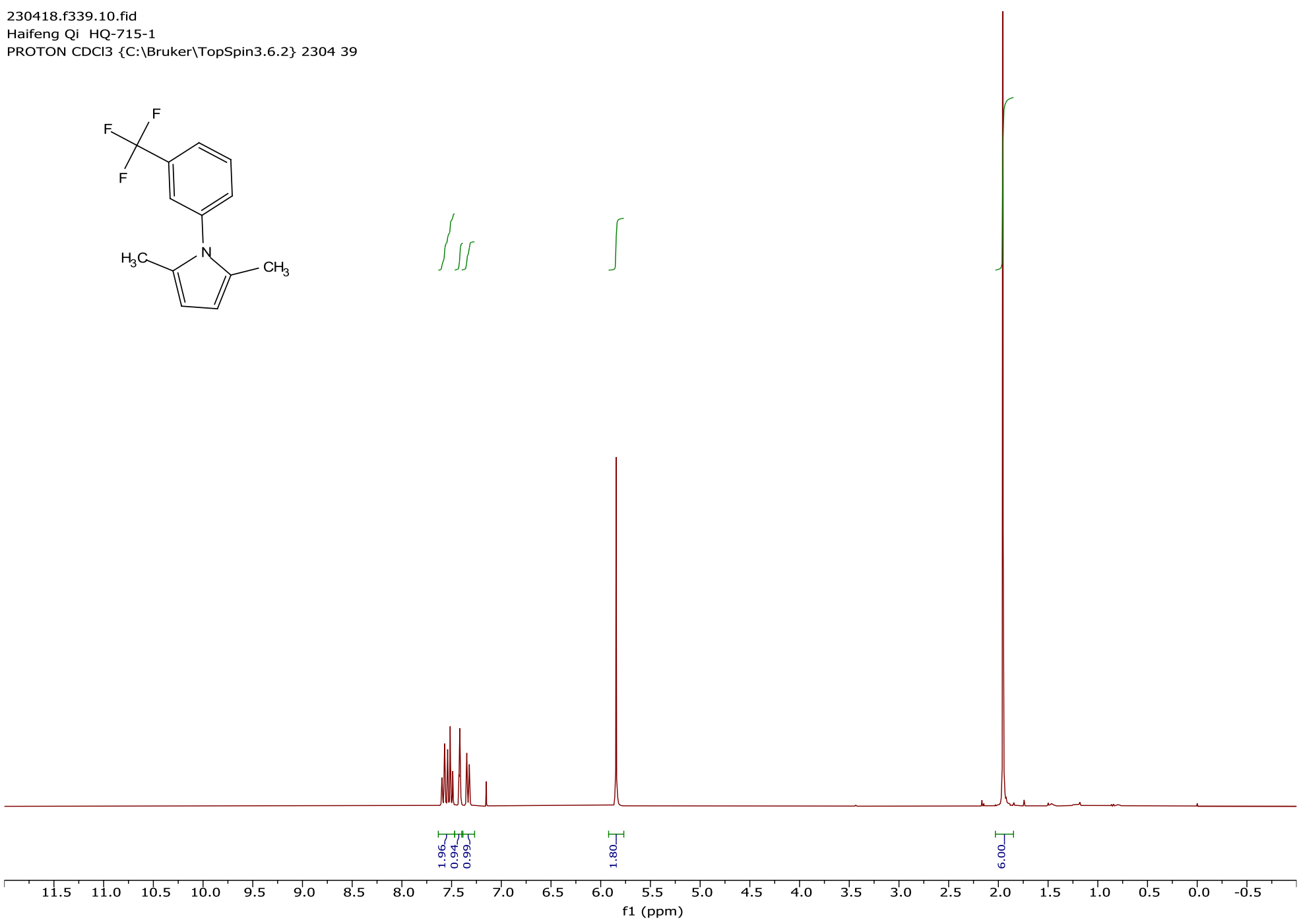

230418.f339.11.fid
Haifeng Qi HQ-715-1
C13CPD CDCl3 {C:\Bruker\TopSpin3.6.2} 2304 39

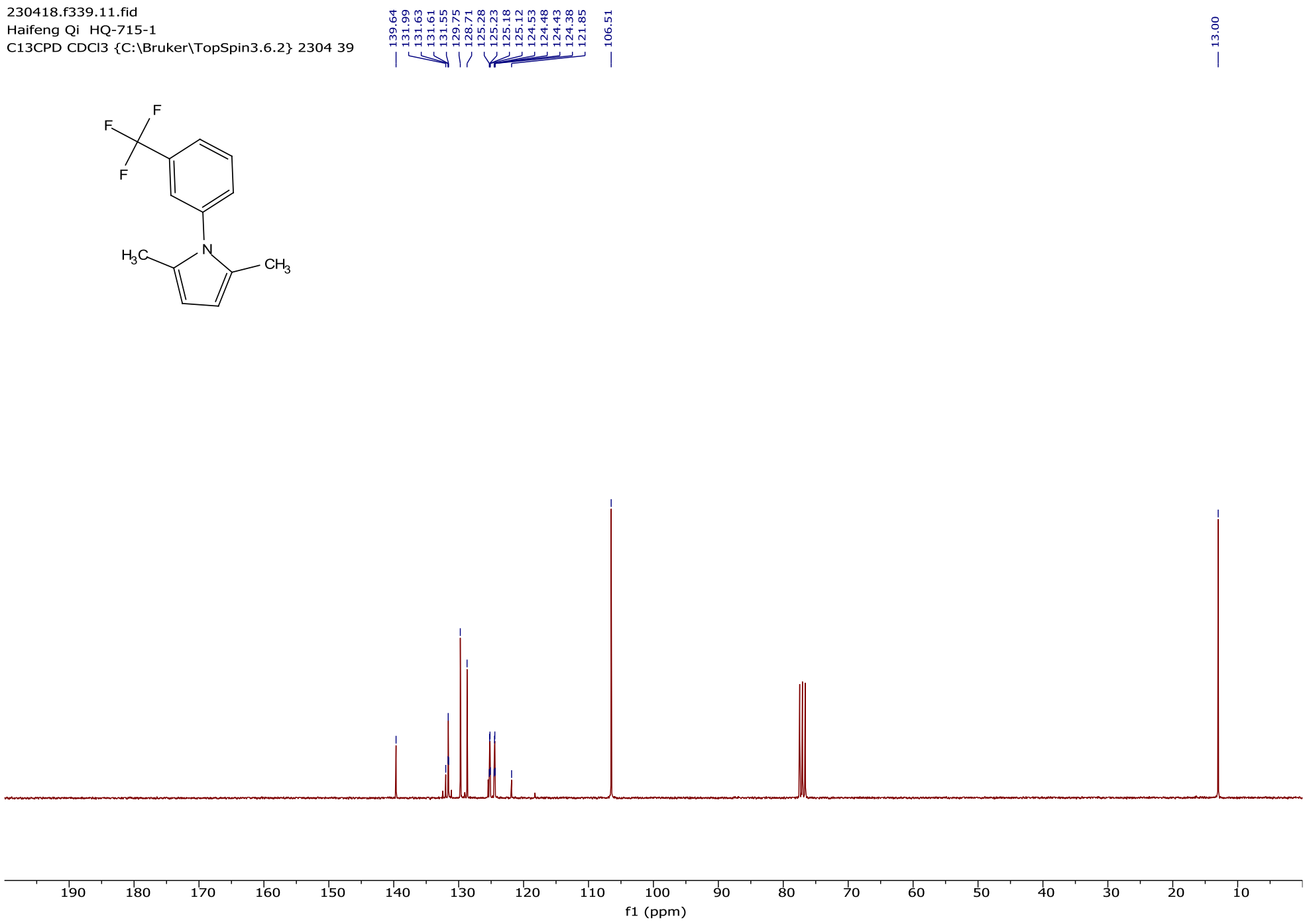

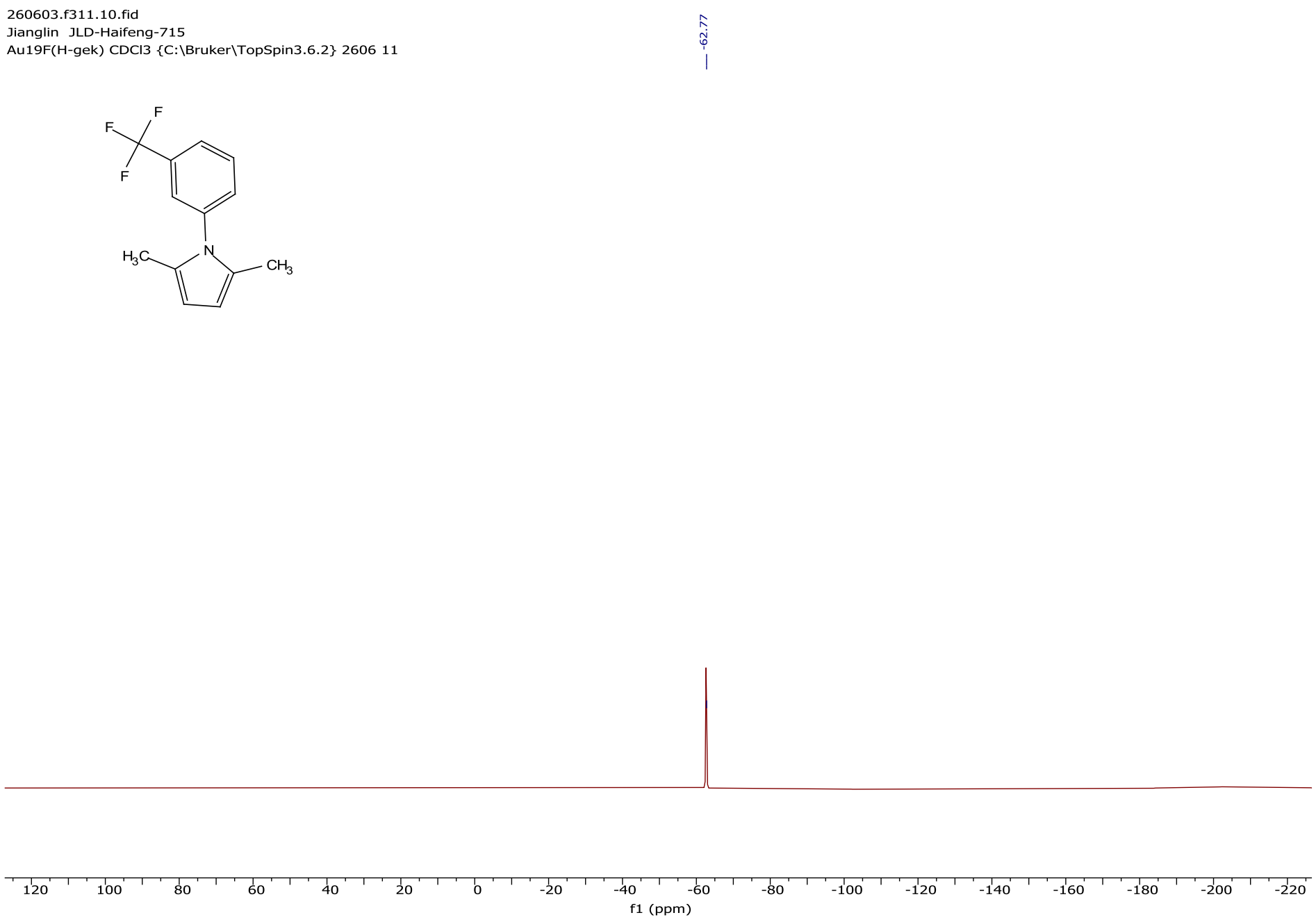

Supplementary Figure 31. ^{1}H, ^{13}C and ^{19}F NMR spectra of product **15c**.

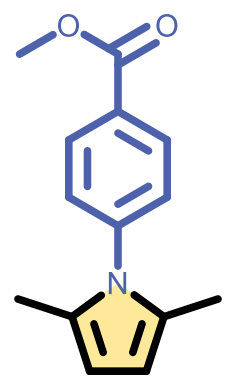

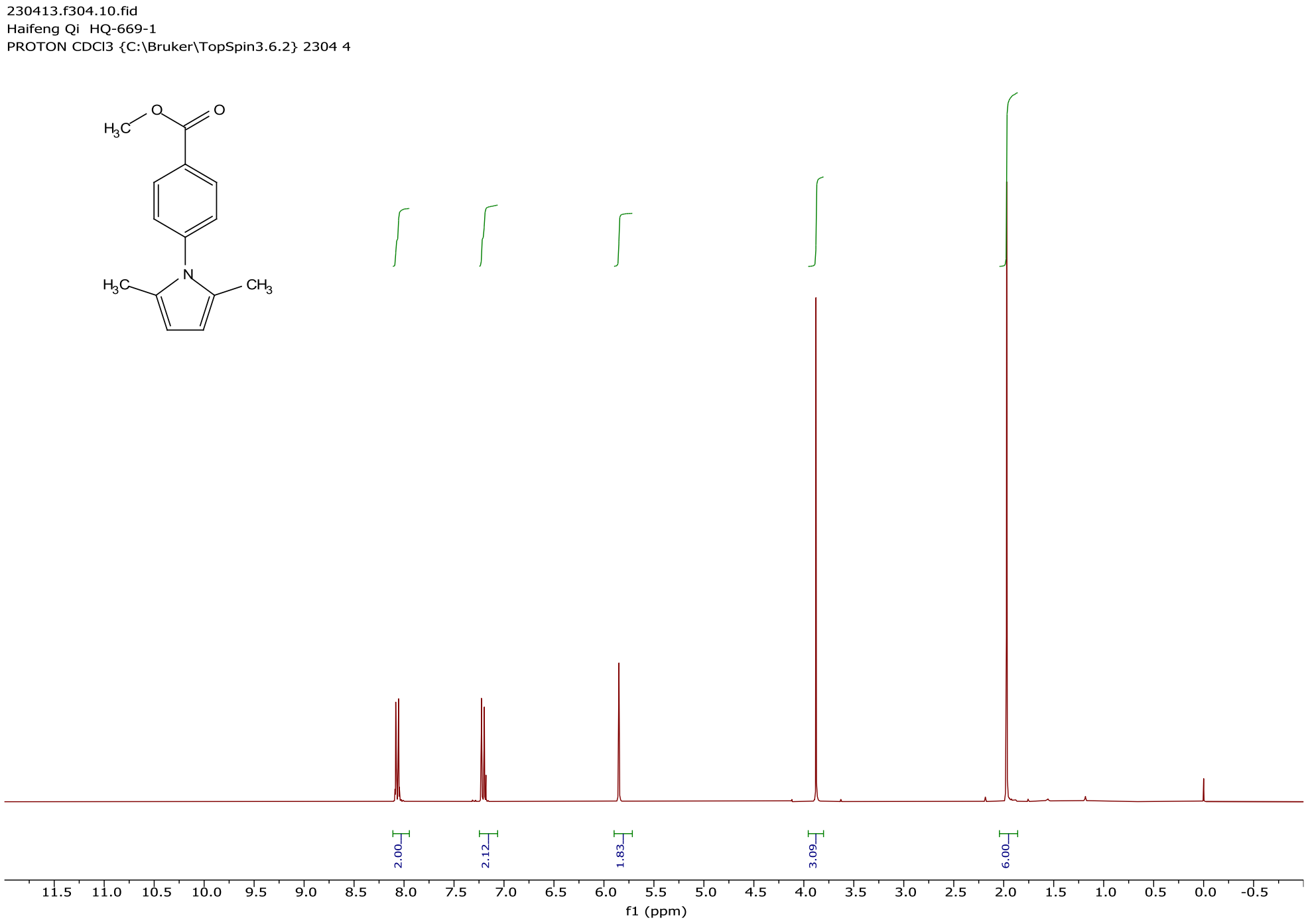

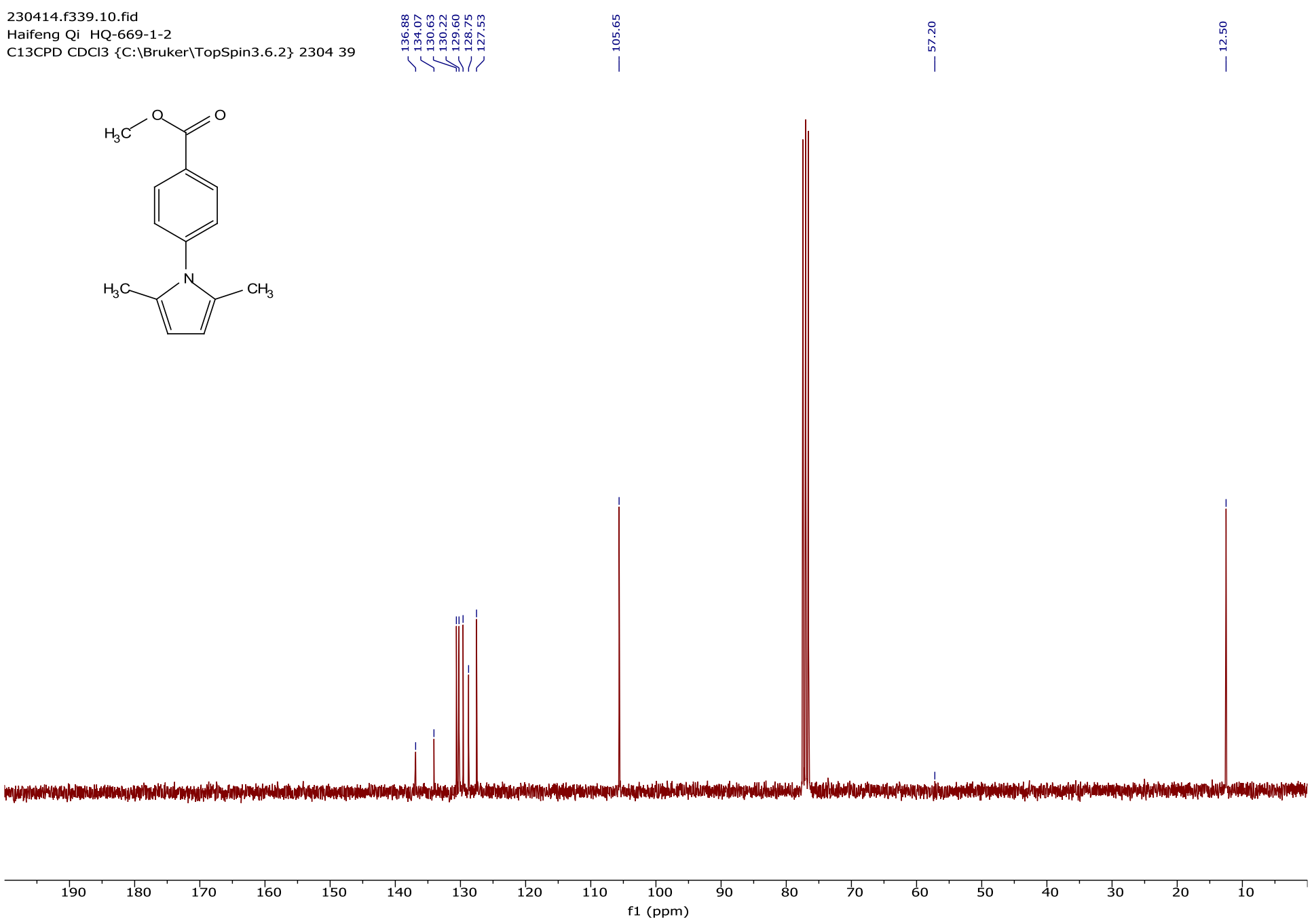

Supplementary Figure 32. ^{1}H and ^{13}C NMR spectra of product **16c**.

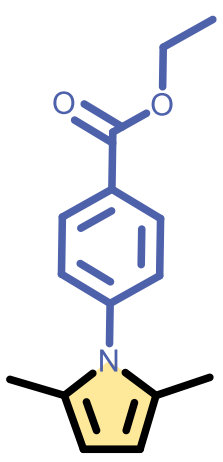

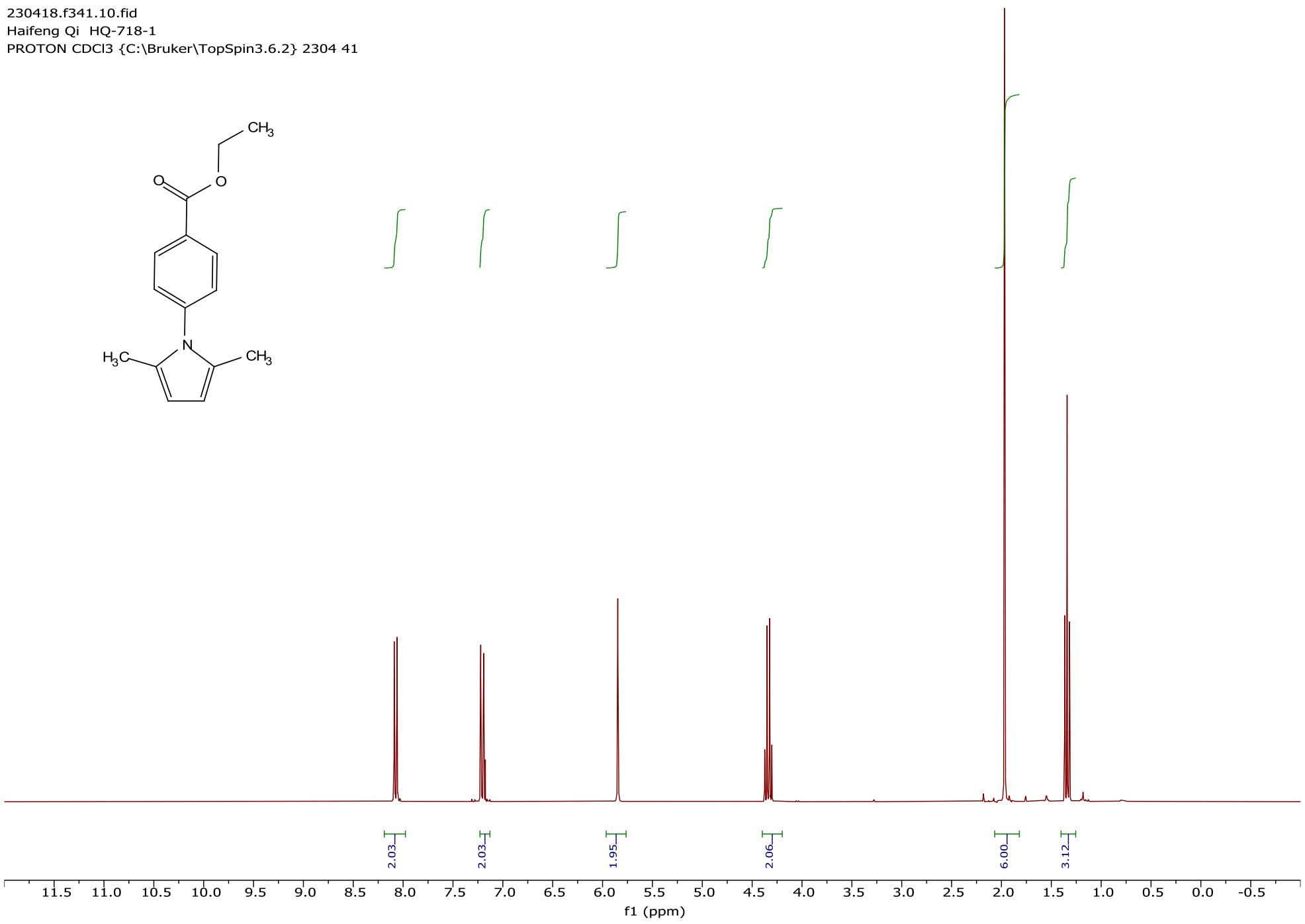

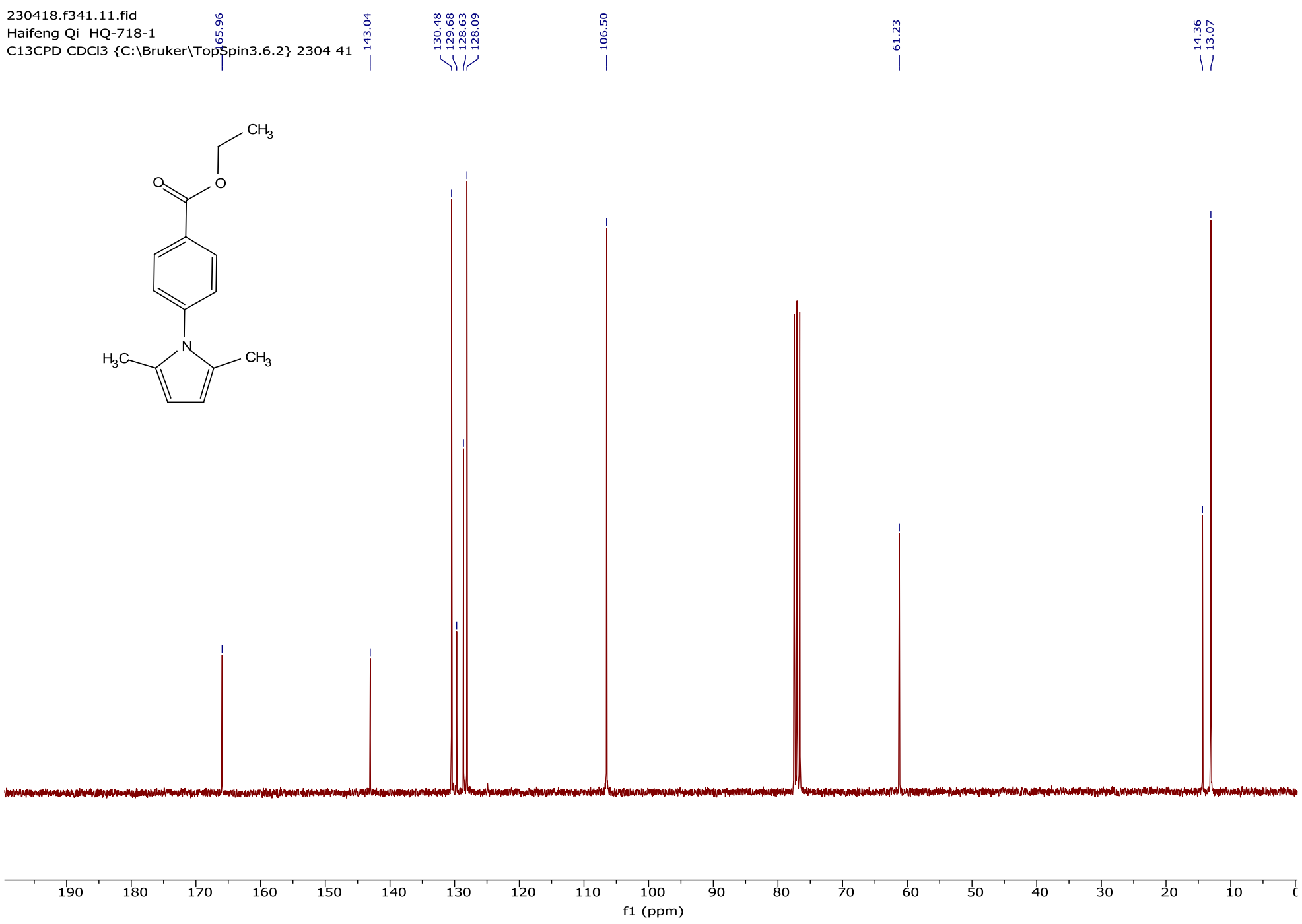

Supplementary Figure 33. ^{1}H and ^{13}C NMR spectra of product **17c**.

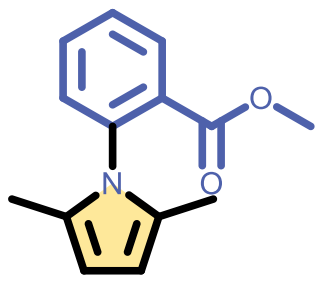

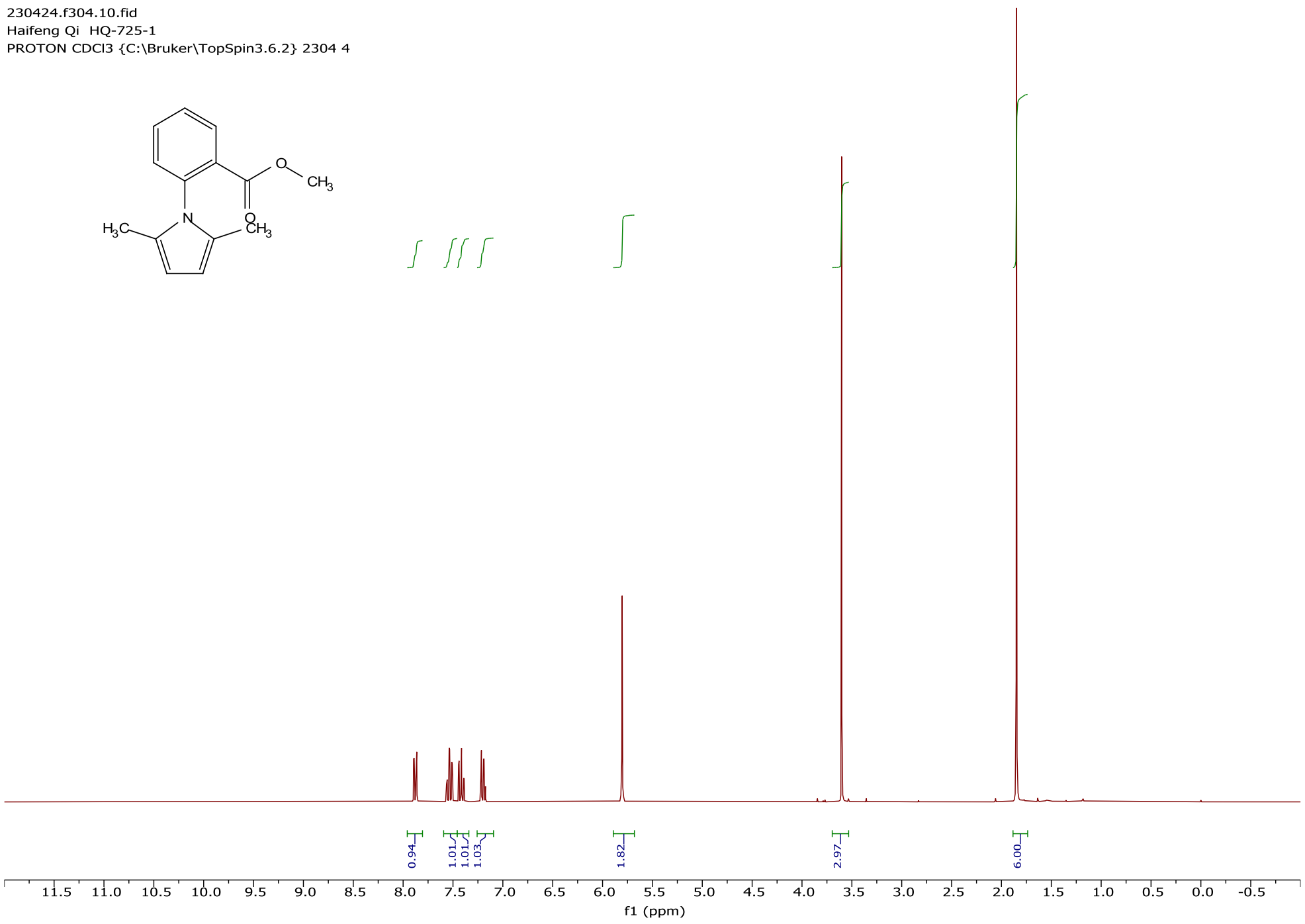

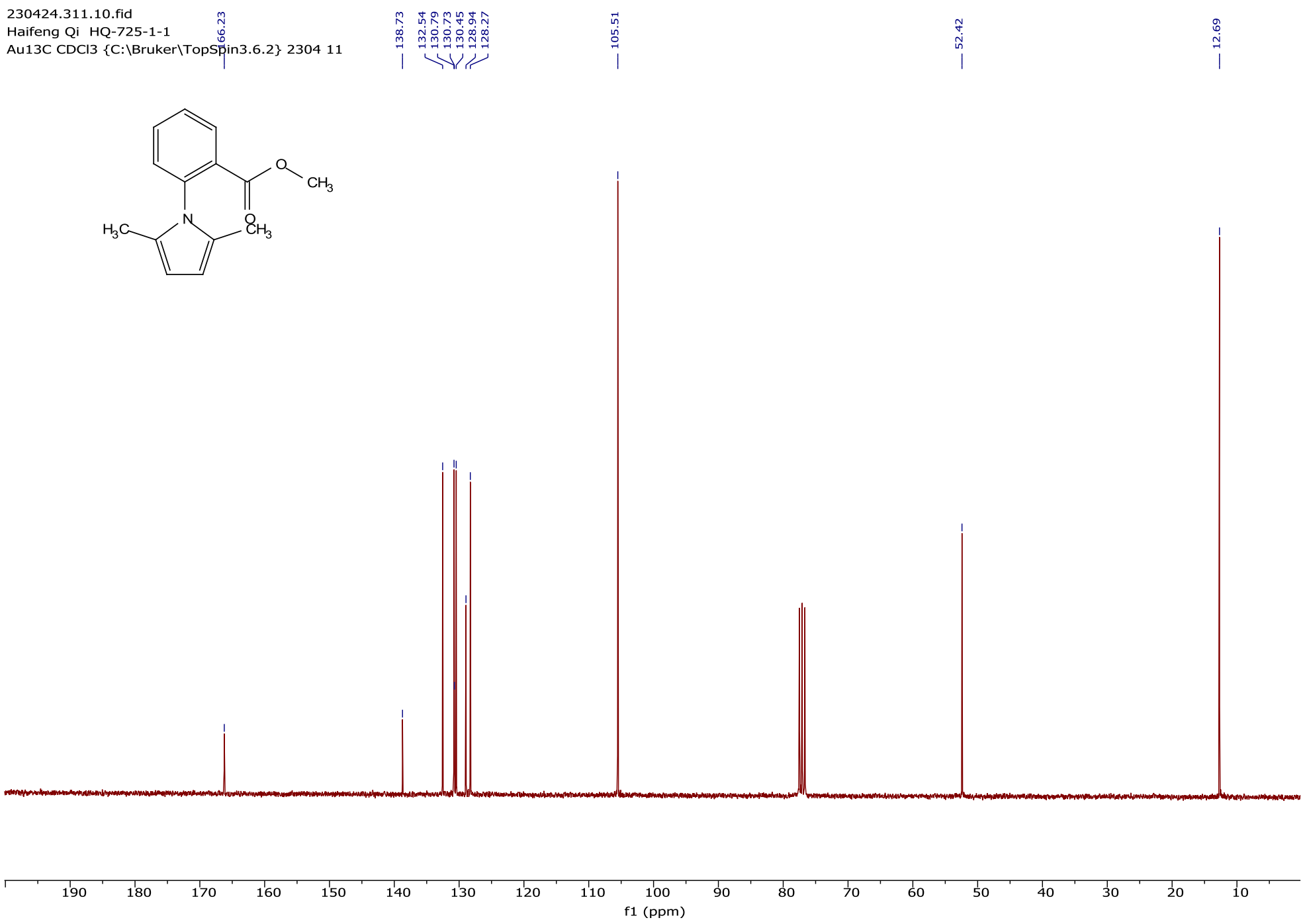

Supplementary Figure 34. ^{1}H and ^{13}C NMR spectra of product **18c**.

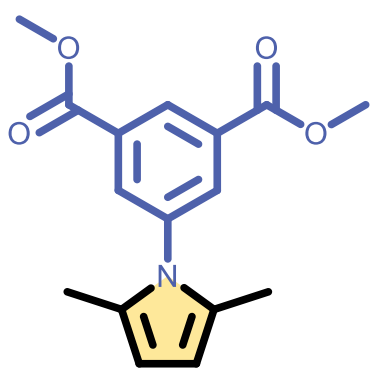

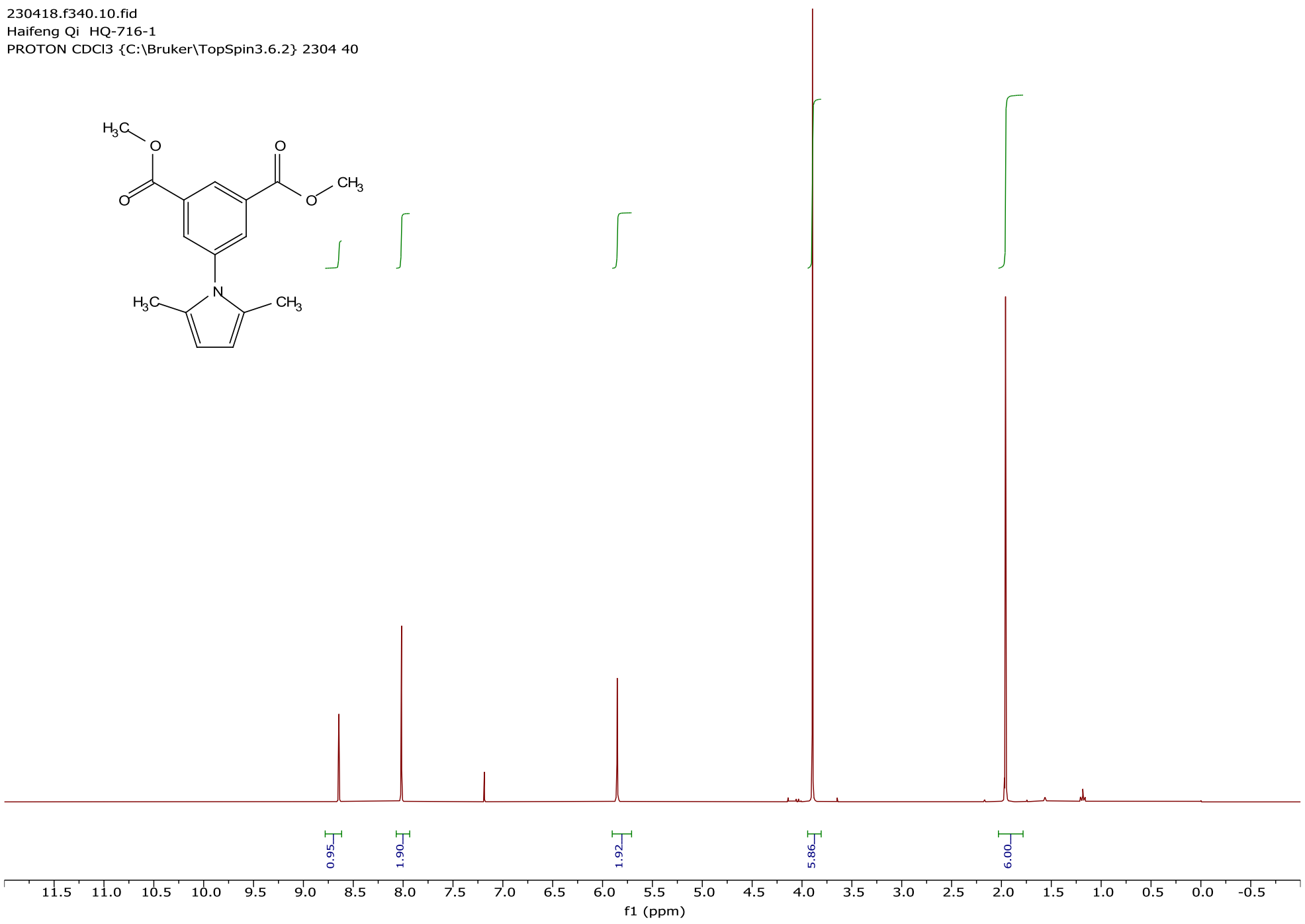

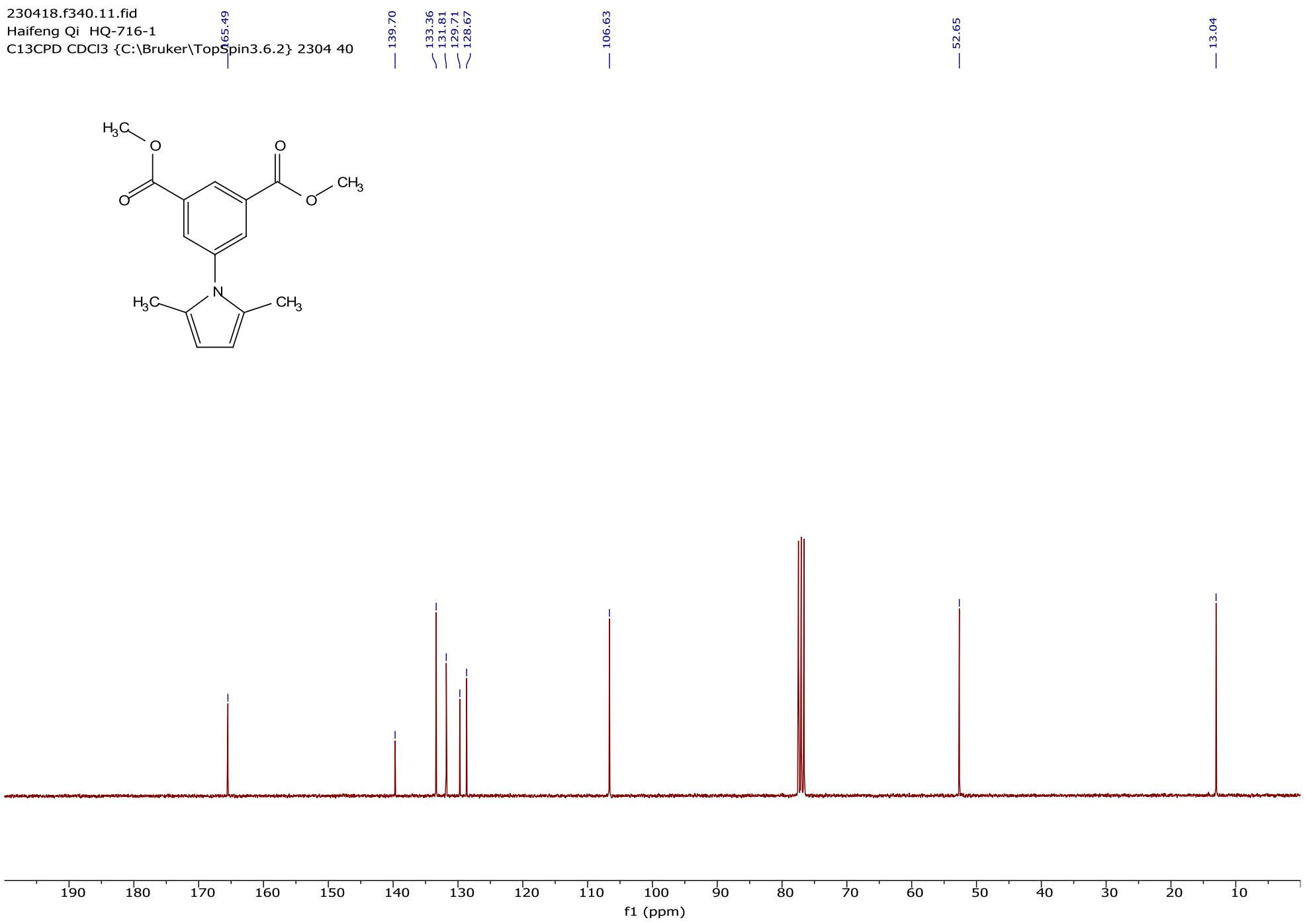

Supplementary Figure 35. ^{1}H and ^{13}C NMR spectra of product **19c**.

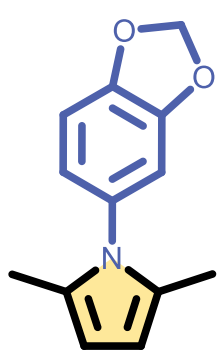

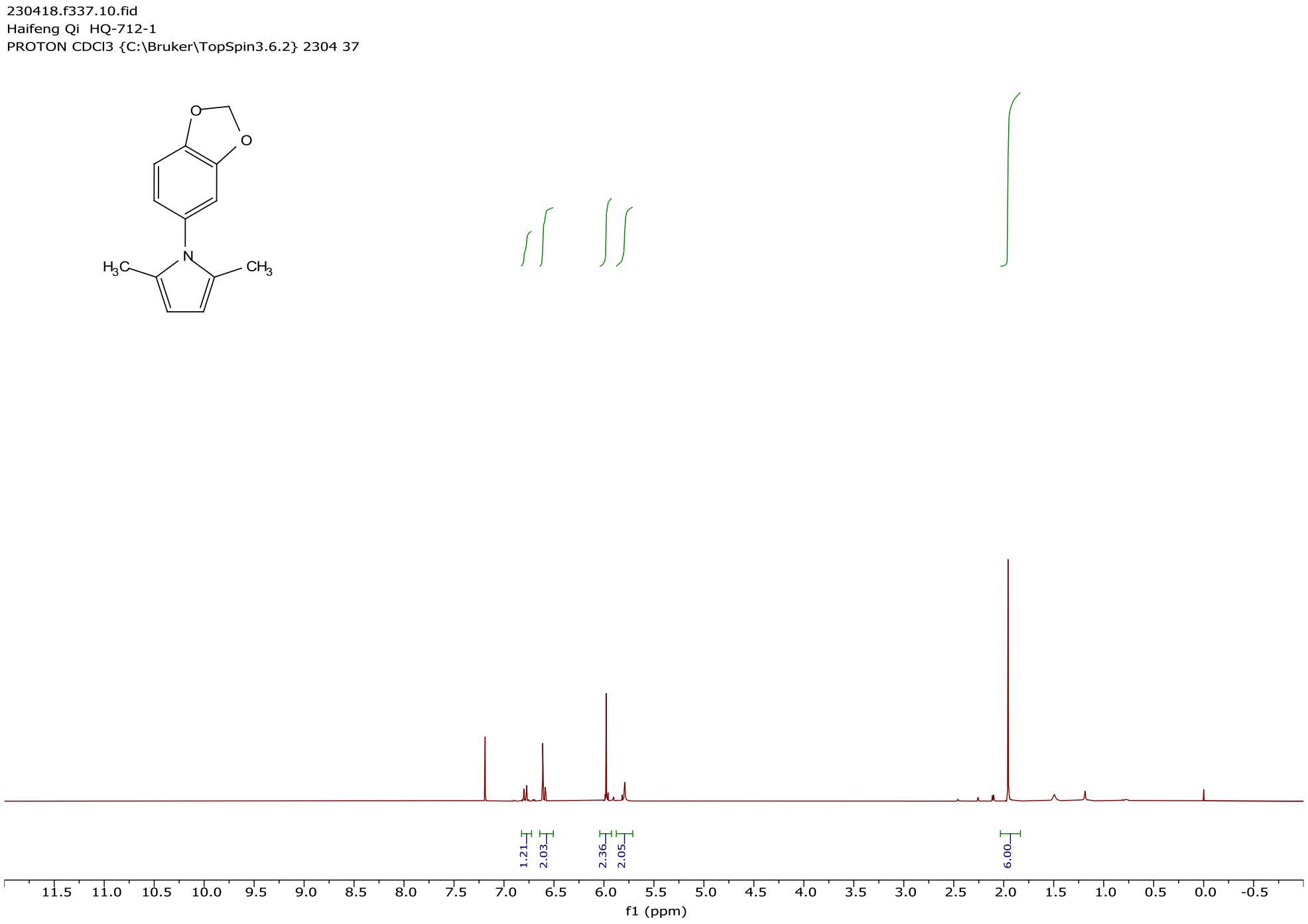

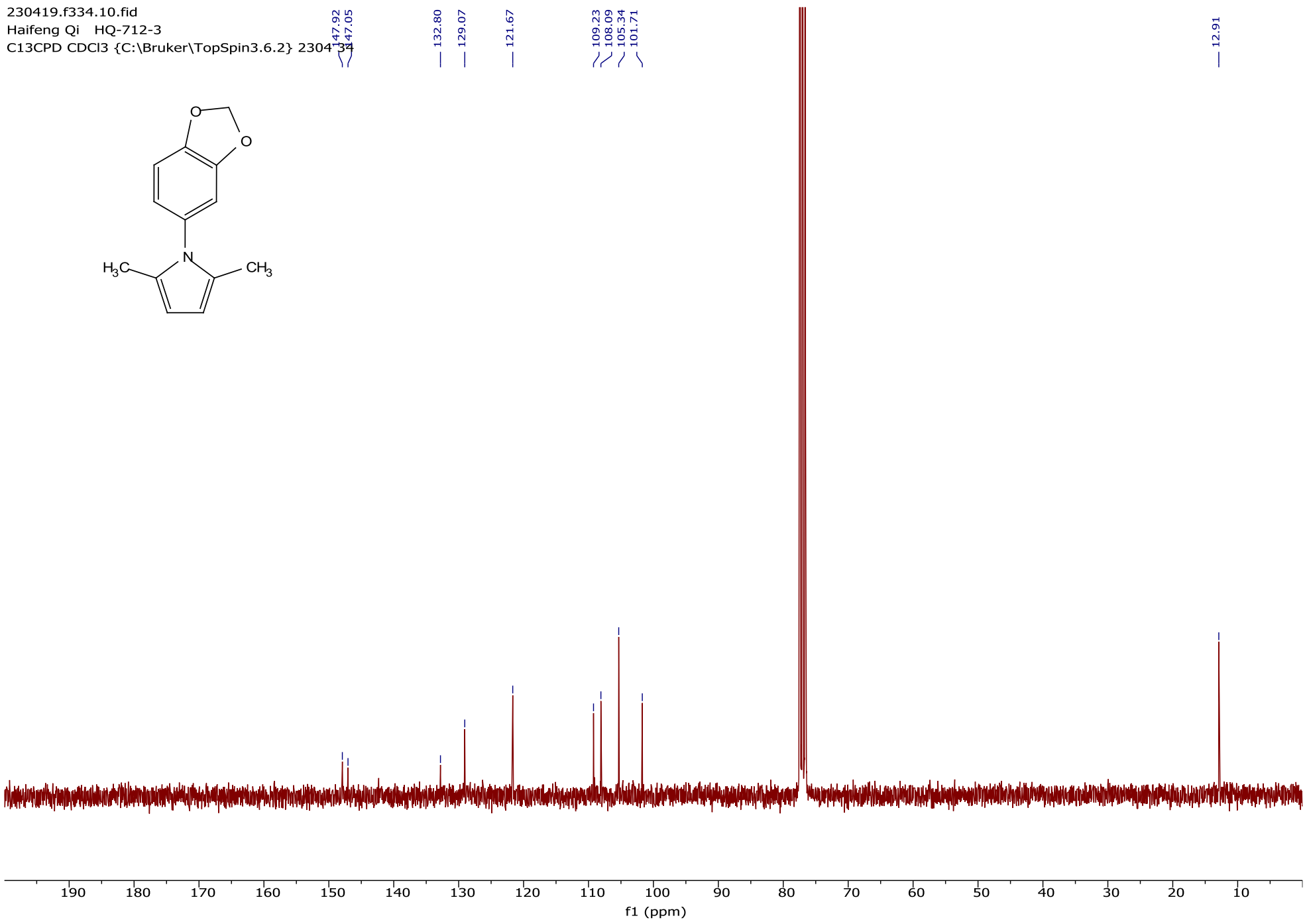

Supplementary Figure 36. ^{1}H and ^{13}C NMR spectra of product **20c**.

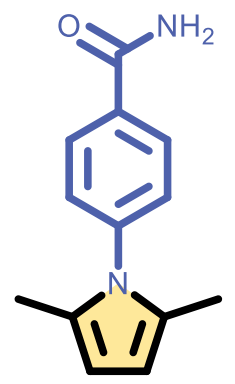

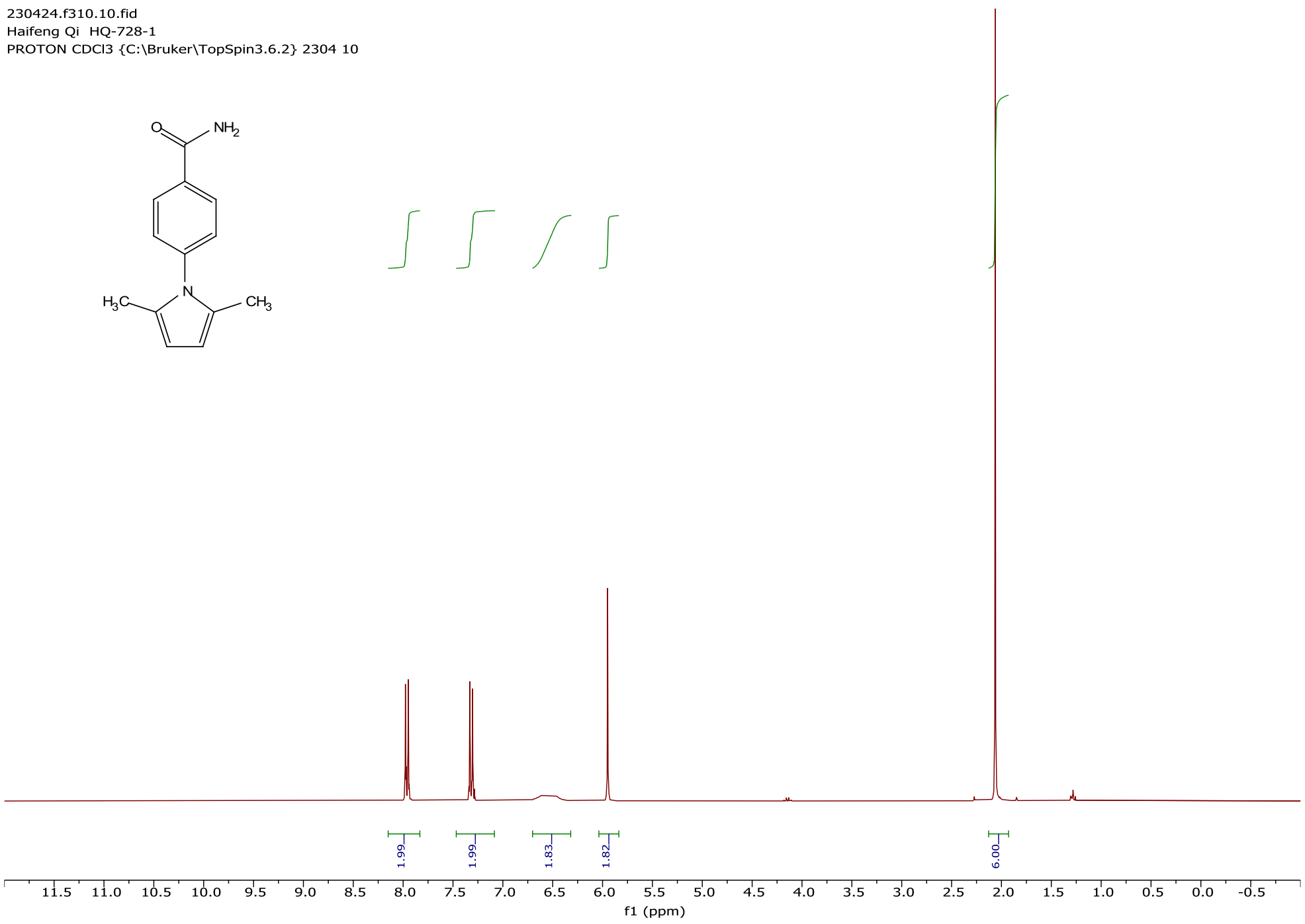

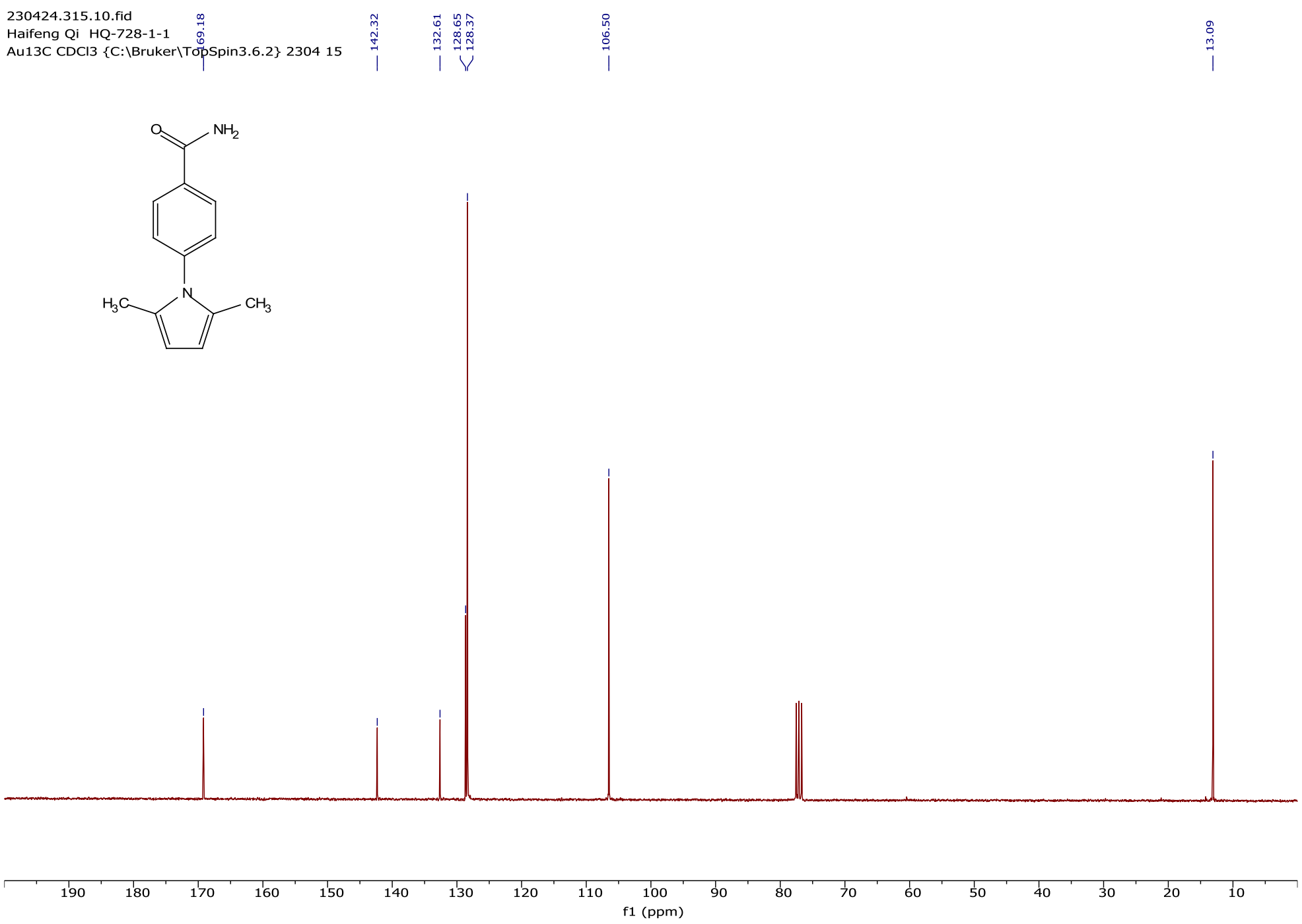

Supplementary Figure 37. ^{1}H and ^{13}C NMR spectra of product **21c**.

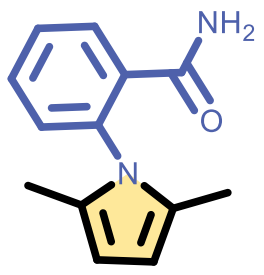

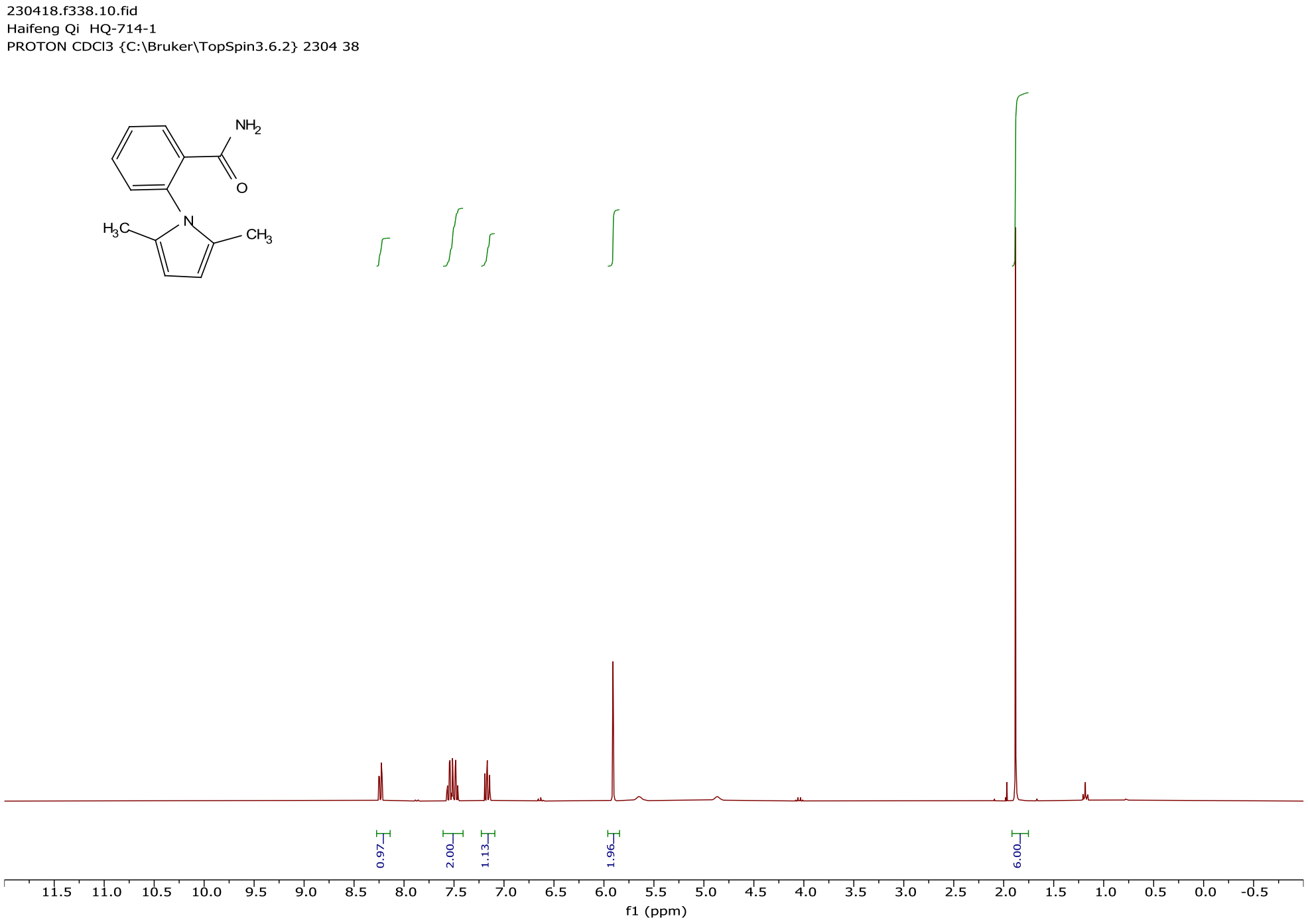

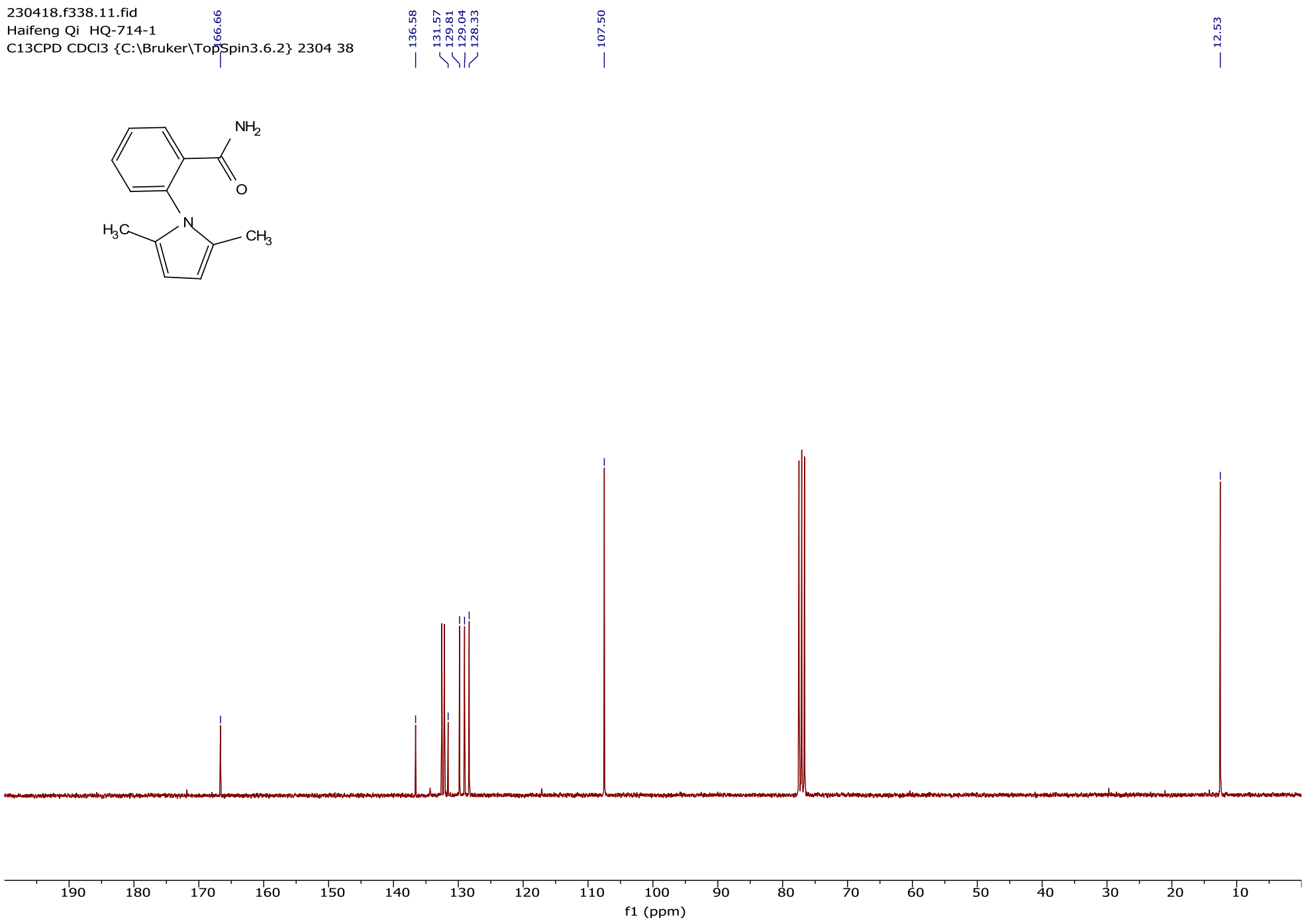

Supplementary Figure 38. ^{1}H and ^{13}C NMR spectra of product **22c**.

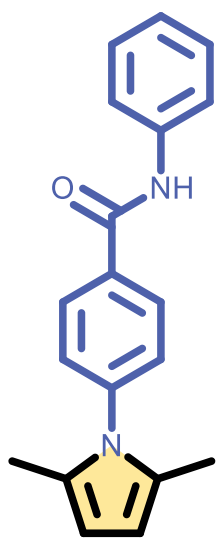

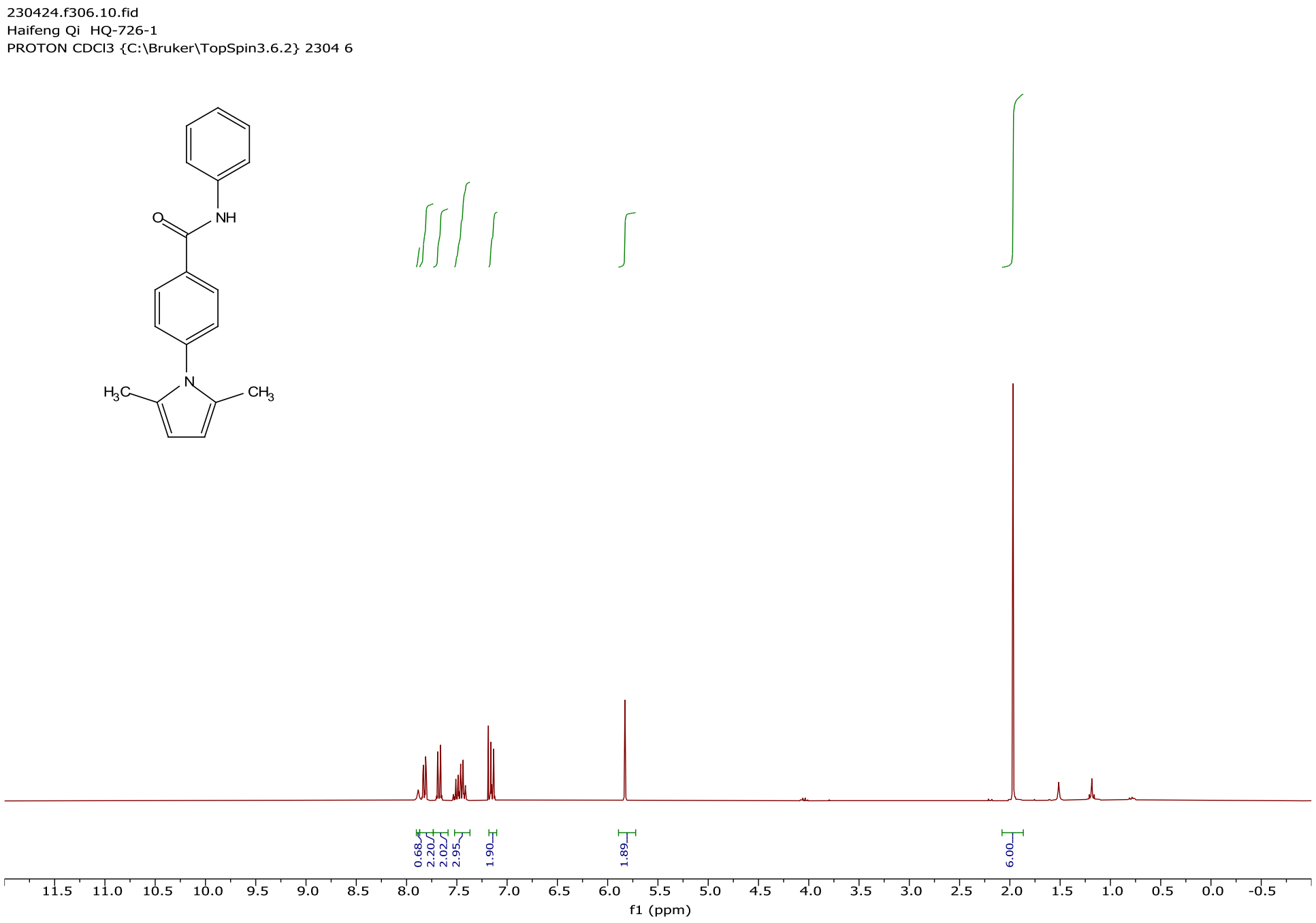

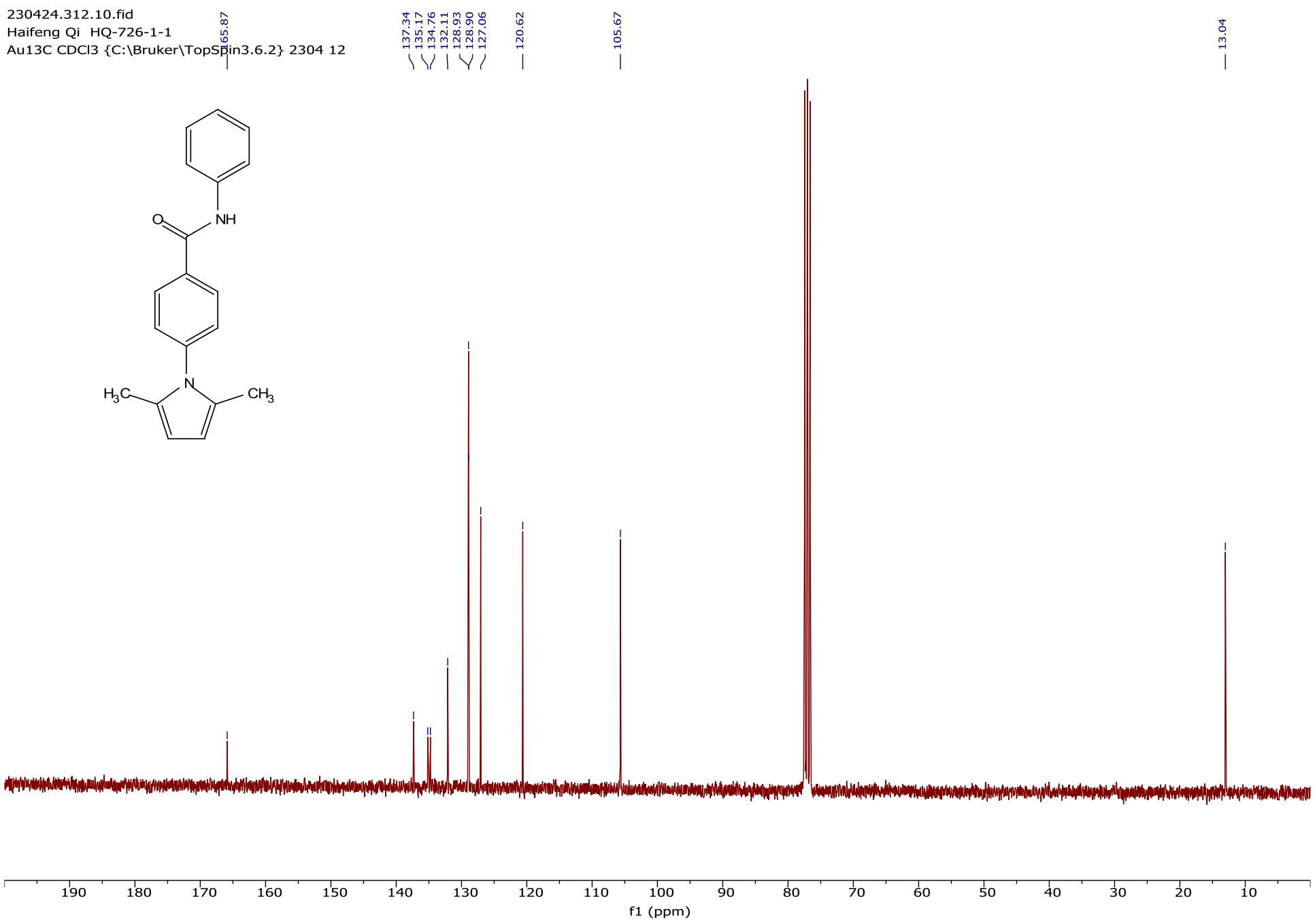

Supplementary Figure 39. ^{1}H and ^{13}C NMR spectra of product **23c**.

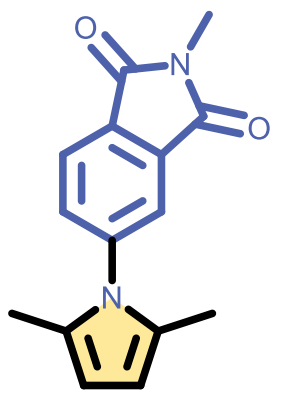

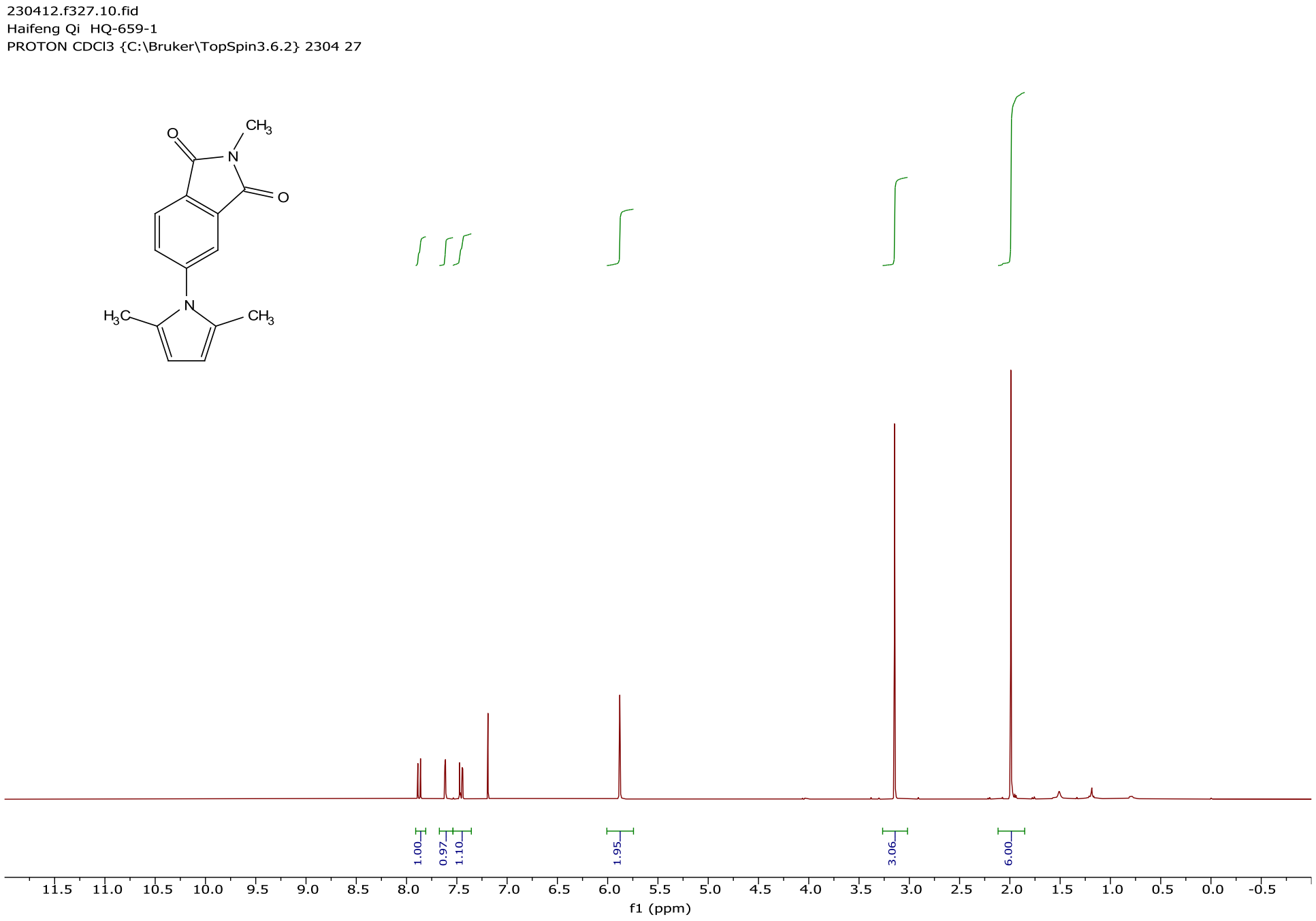

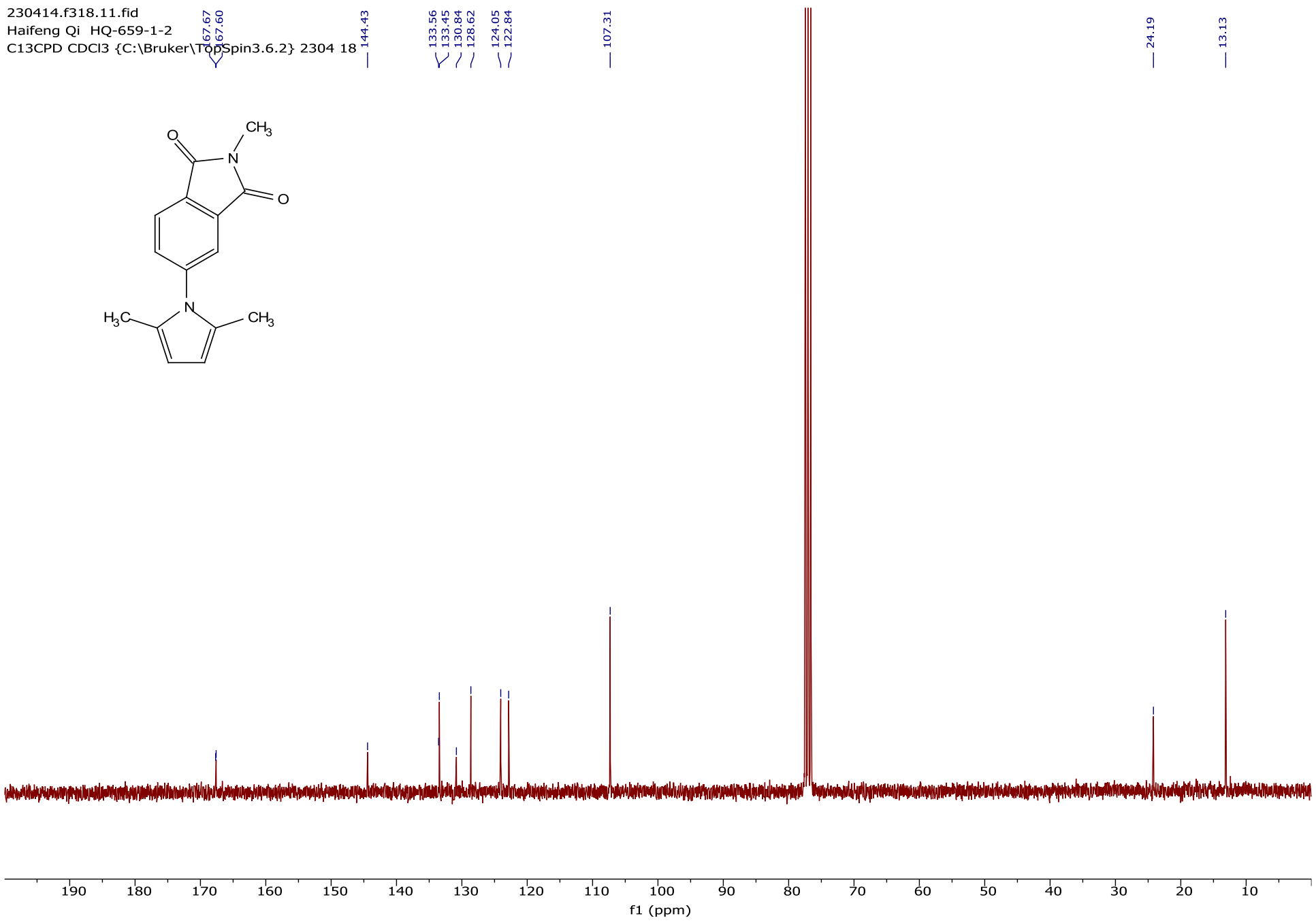

Supplementary Figure 40. ^{1}H and ^{13}C NMR spectra of product **24c**.

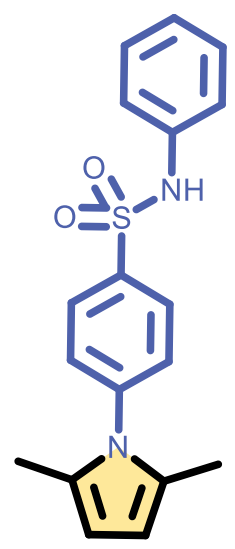

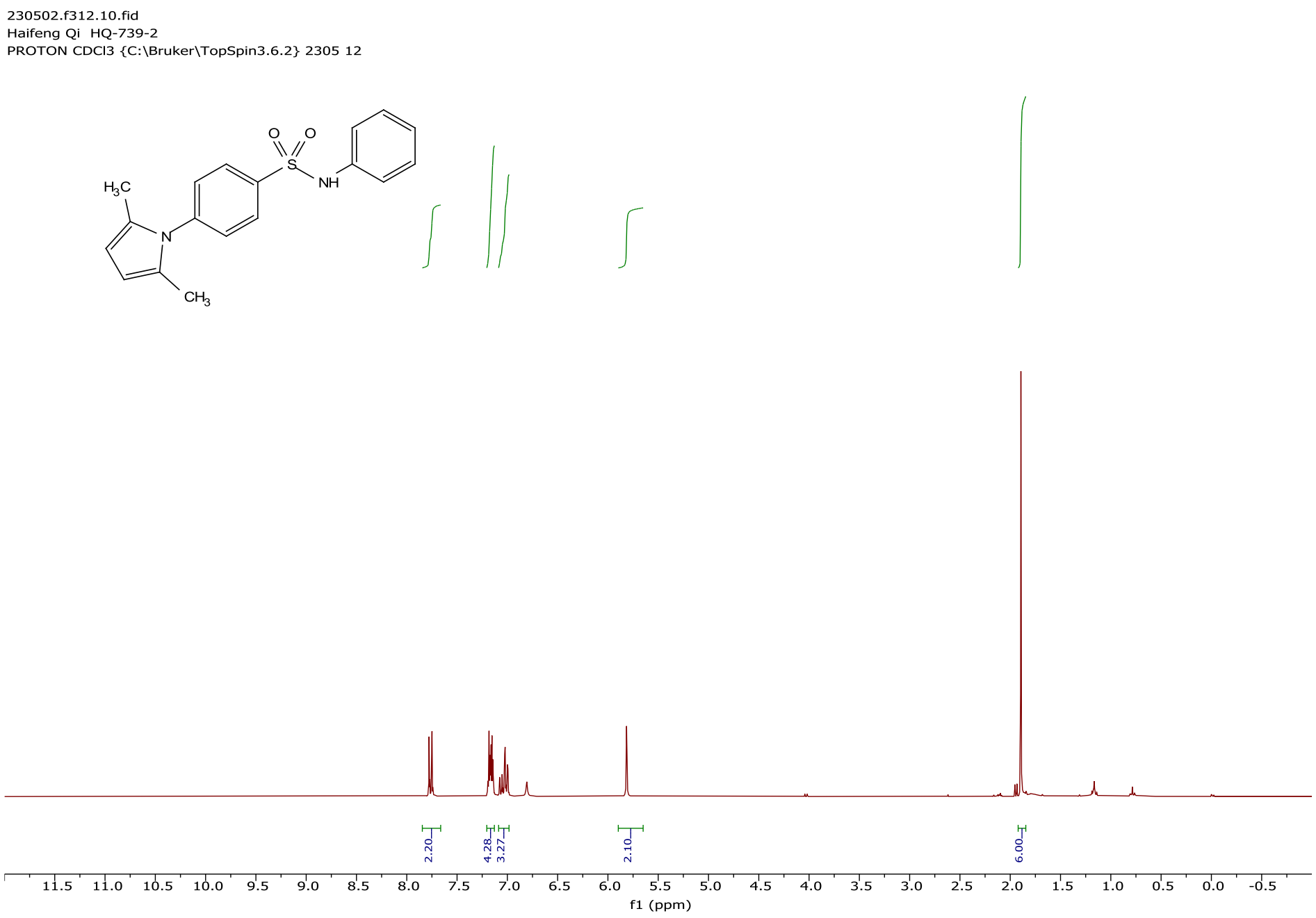

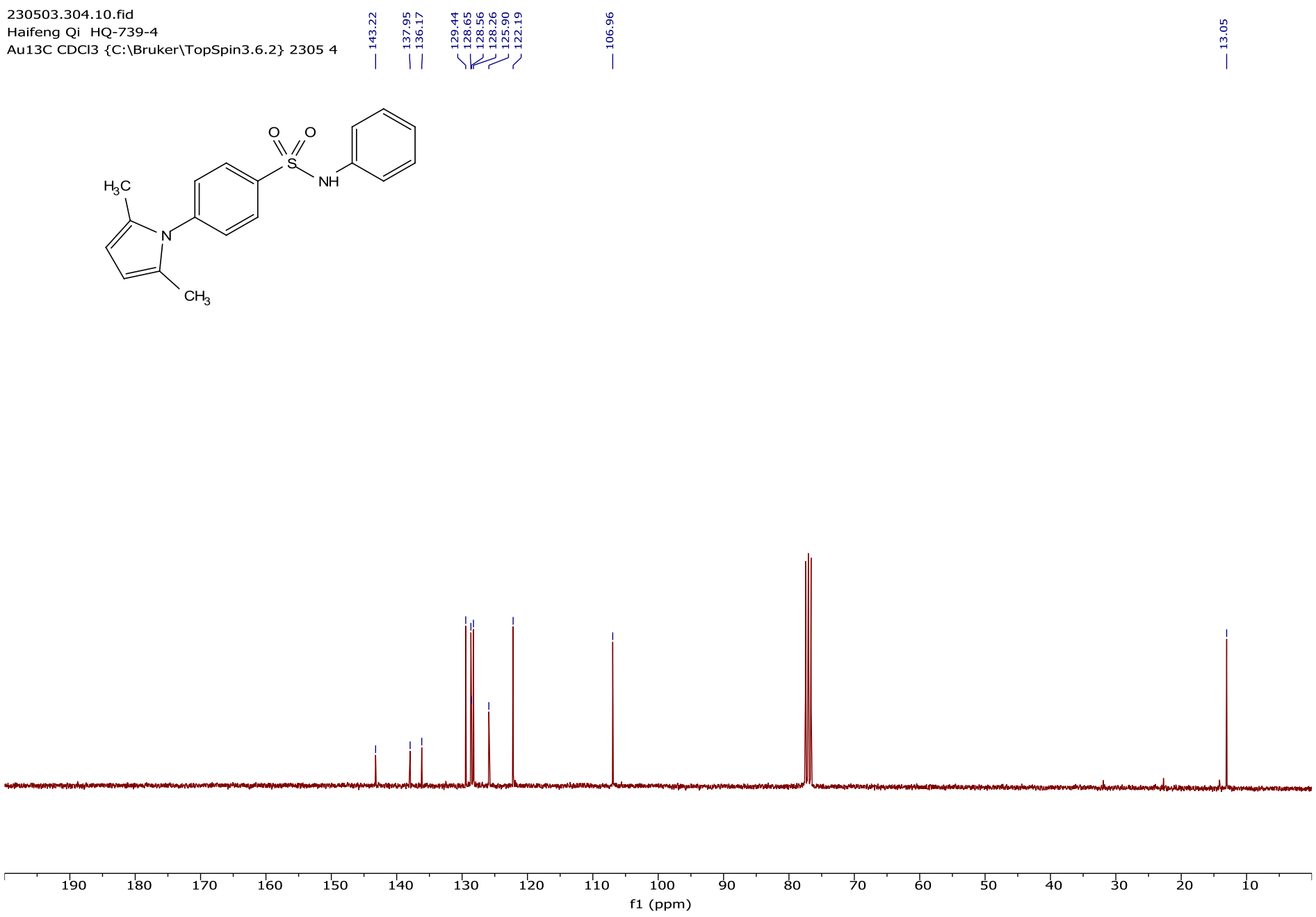

Supplementary Figure 41. ^{1}H and ^{13}C NMR spectra of product **25c**.

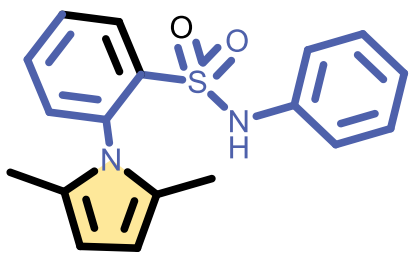

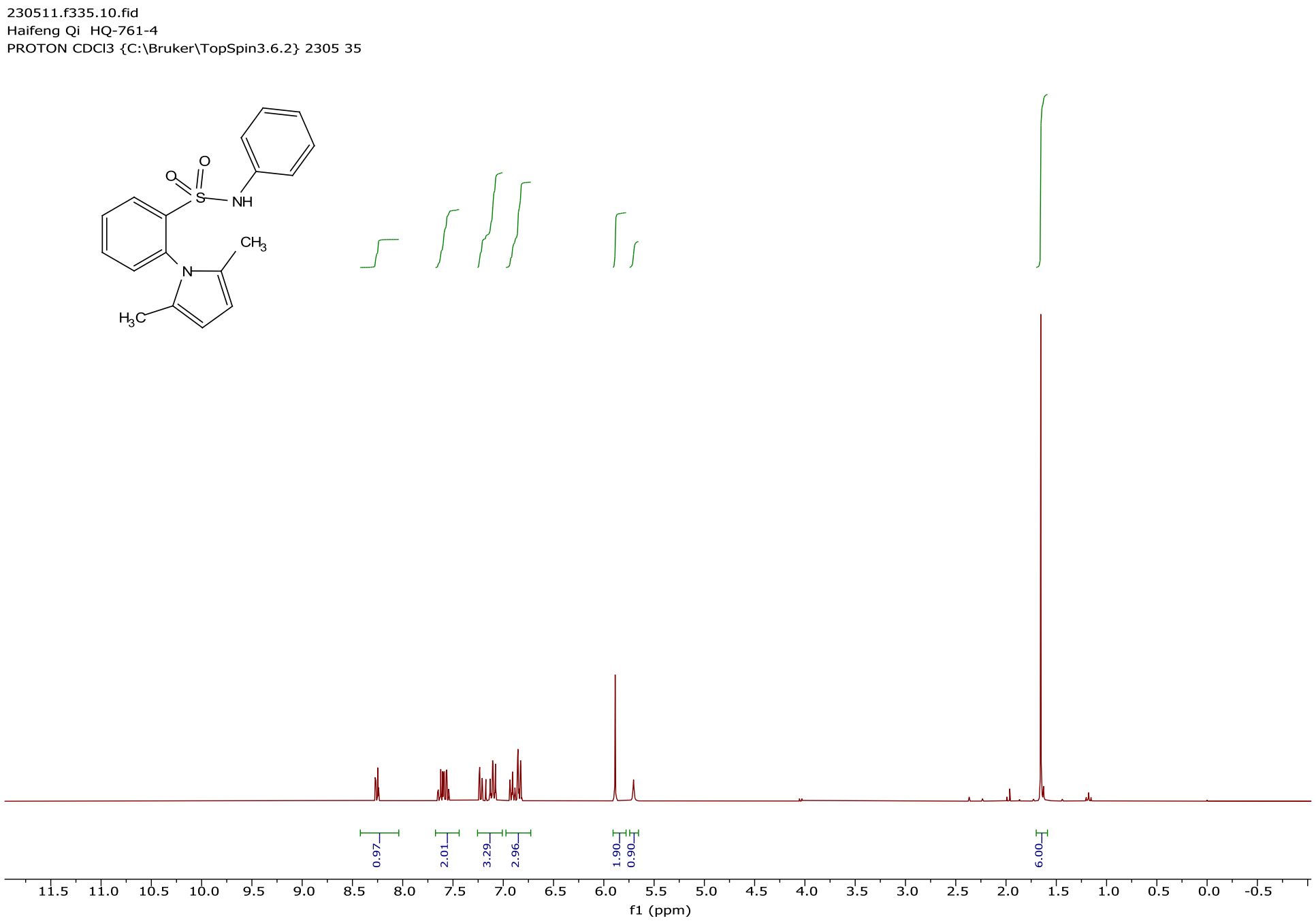

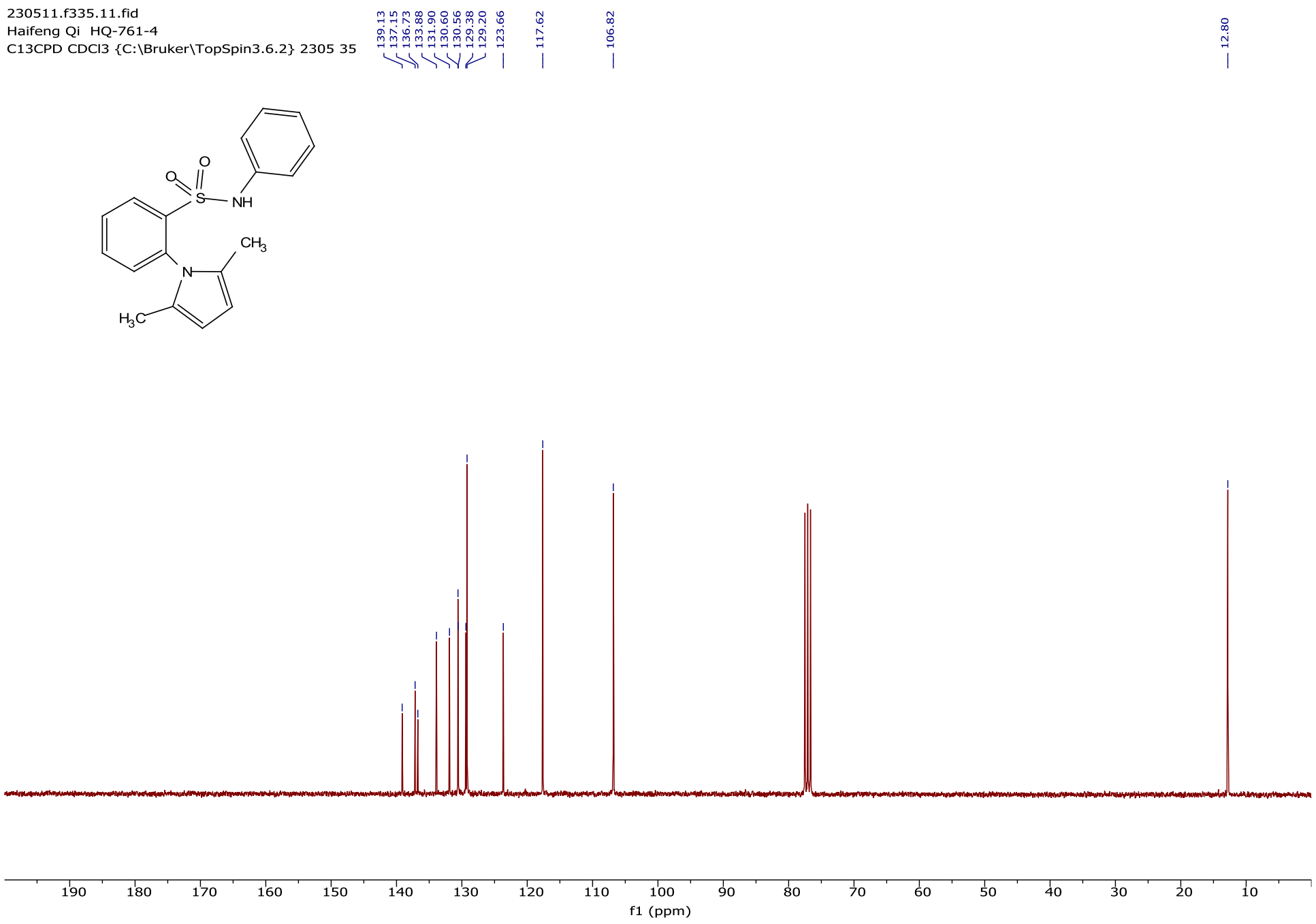

Supplementary Figure 42. ^{1}H and ^{13}C NMR spectra of product **26c**.

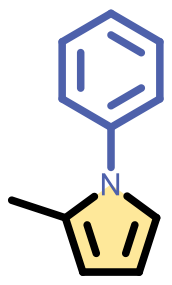

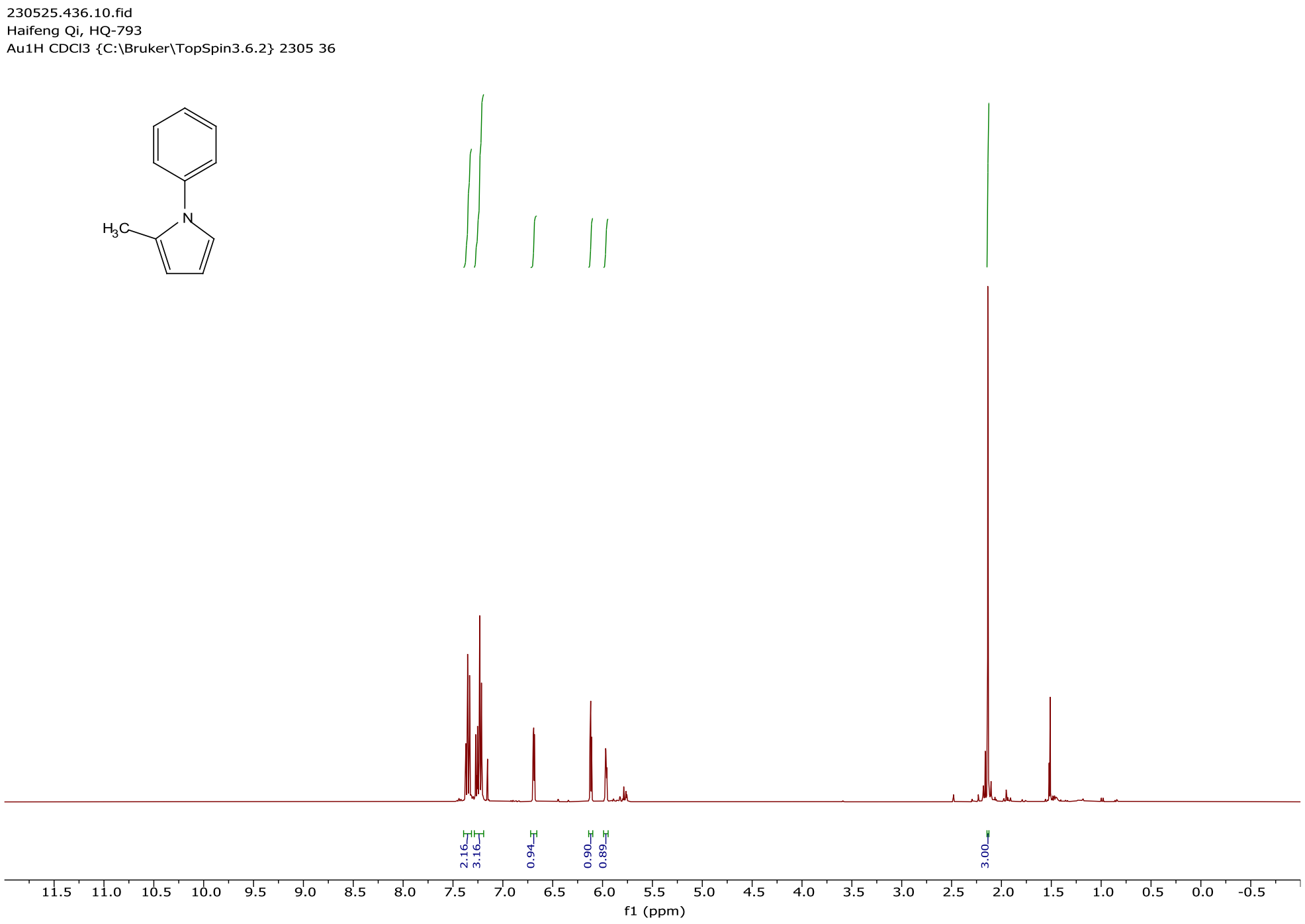

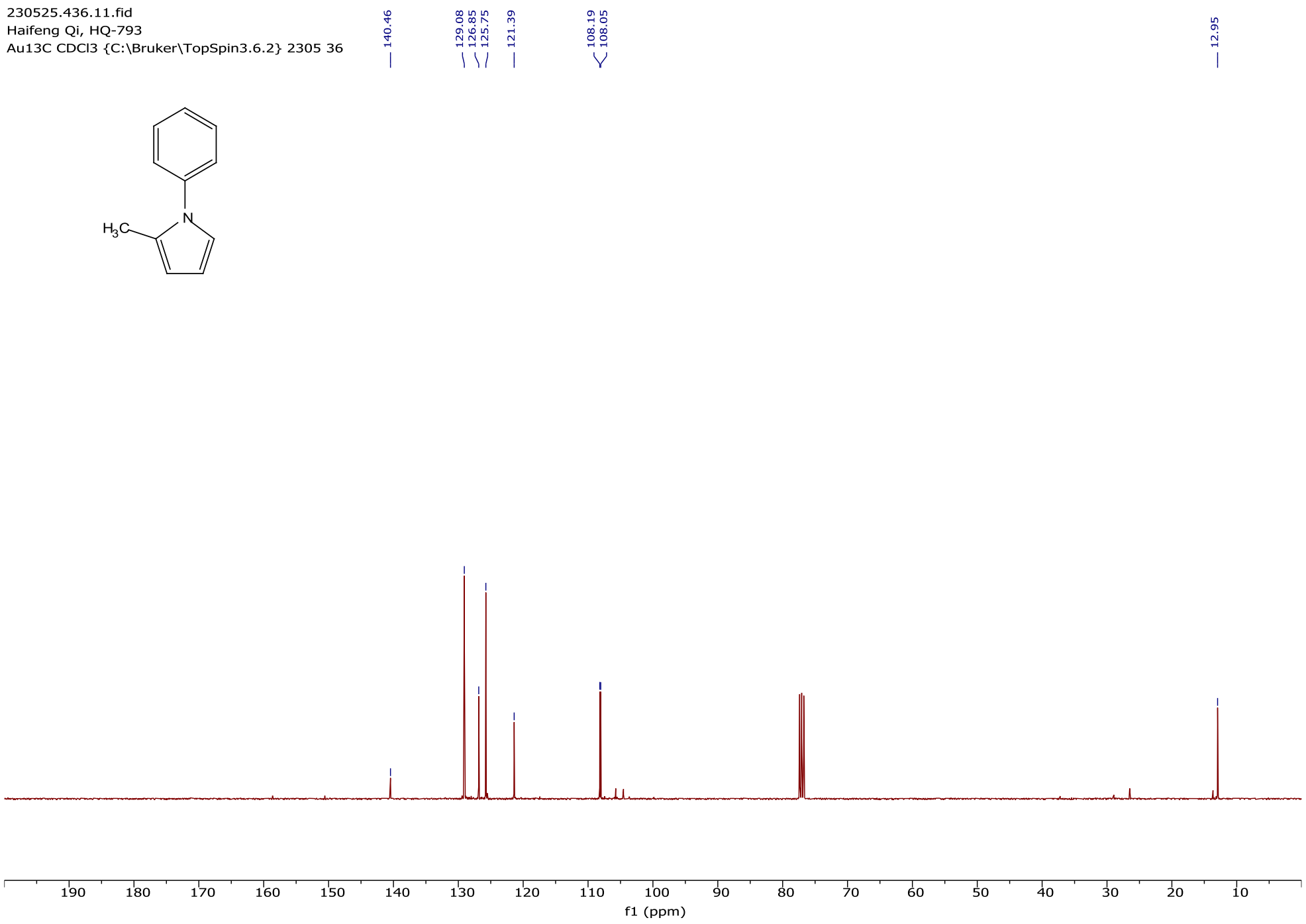

Supplementary Figure 43. ^{1}H and ^{13}C NMR spectra of product **27c**.

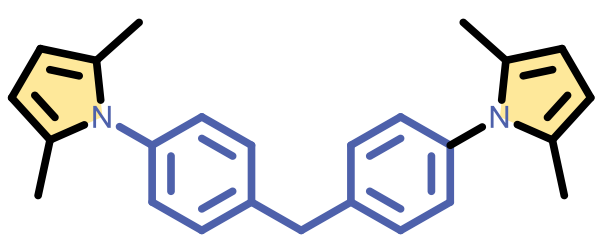

230512.334.10.fid
Haifeng Qi, HQ-759-2
Au1H CDCl3 {C:\Bruker\TopSpin3.6.2} 2305 34

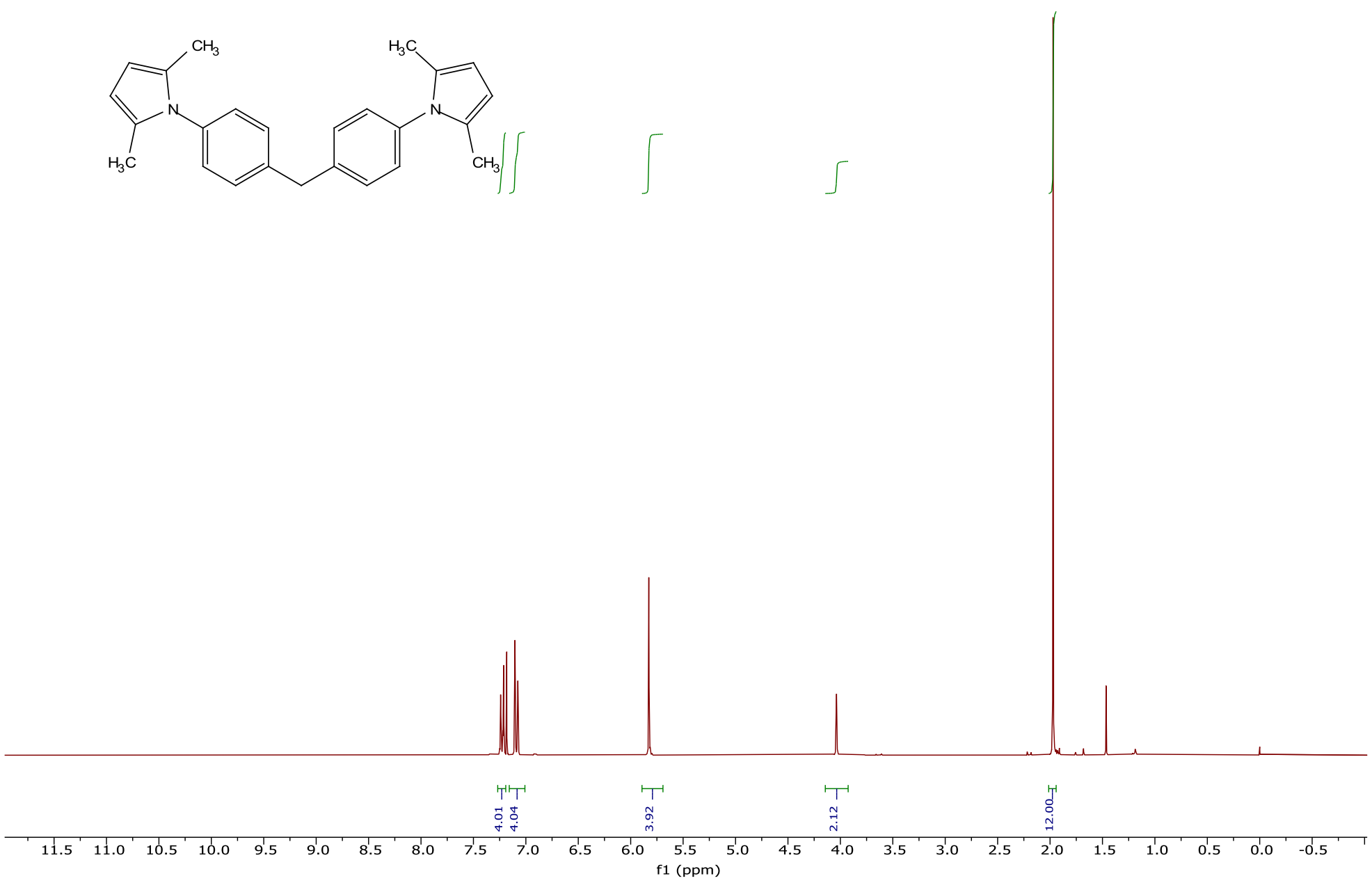

230512.334.11.fid
Haifeng Qi, HQ-759-2
Au13C CDCl3 {C:\Bruker\TopSpin3.6.2} 2305 34

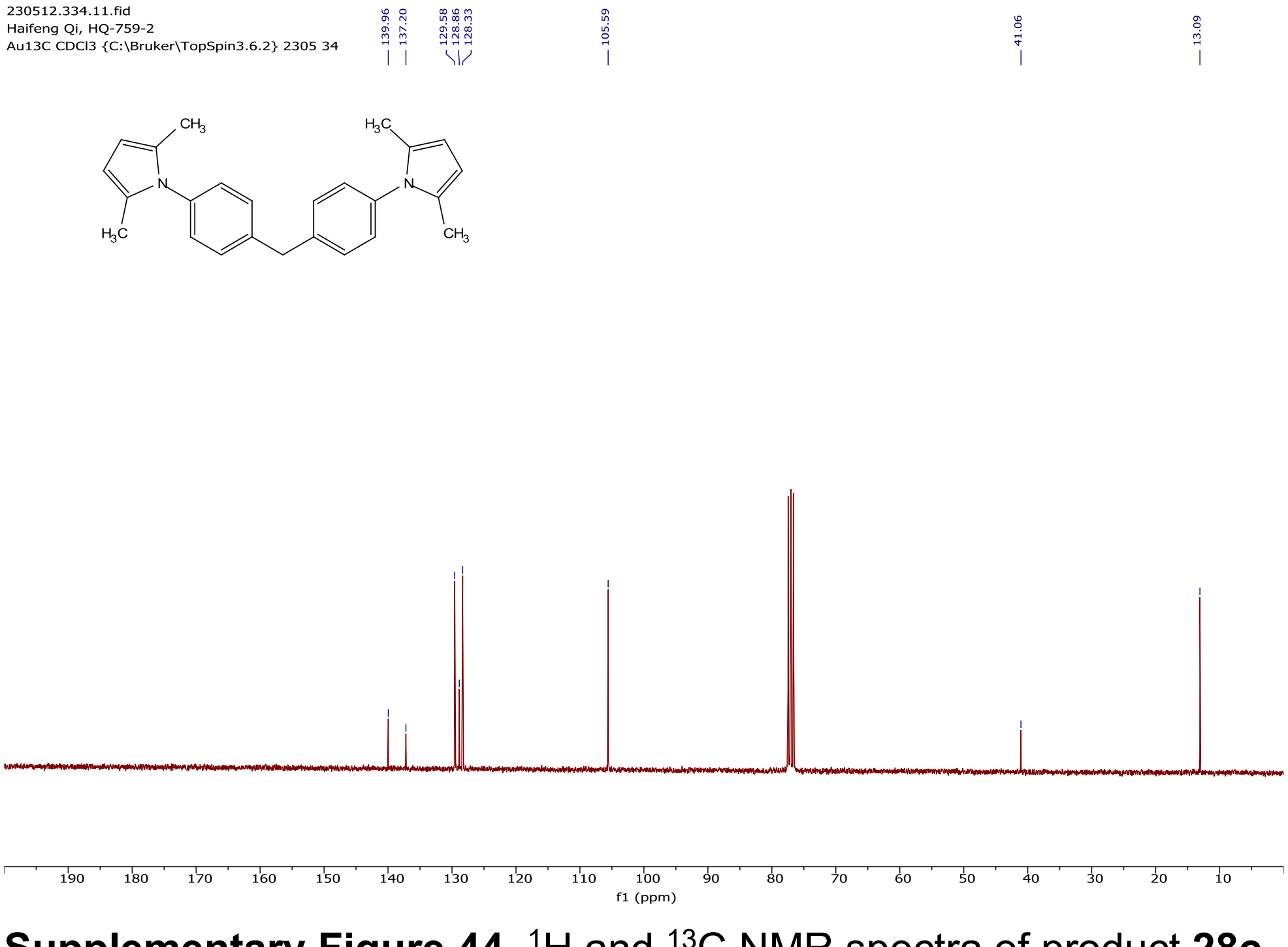

Supplementary Figure 44. ^{1}H and ^{13}C NMR spectra of product **28c**.

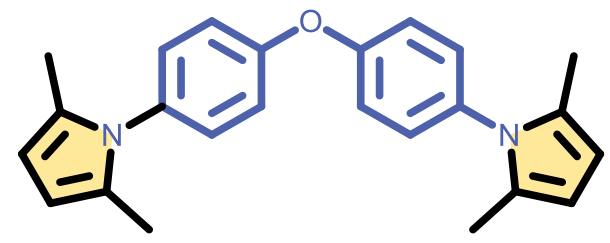

230503.344.10.fid
Haifeng Qi, HQ-742-1
Au1H CDCl3 {C:\Bruker\TopSpin3.6.2} 2305 44

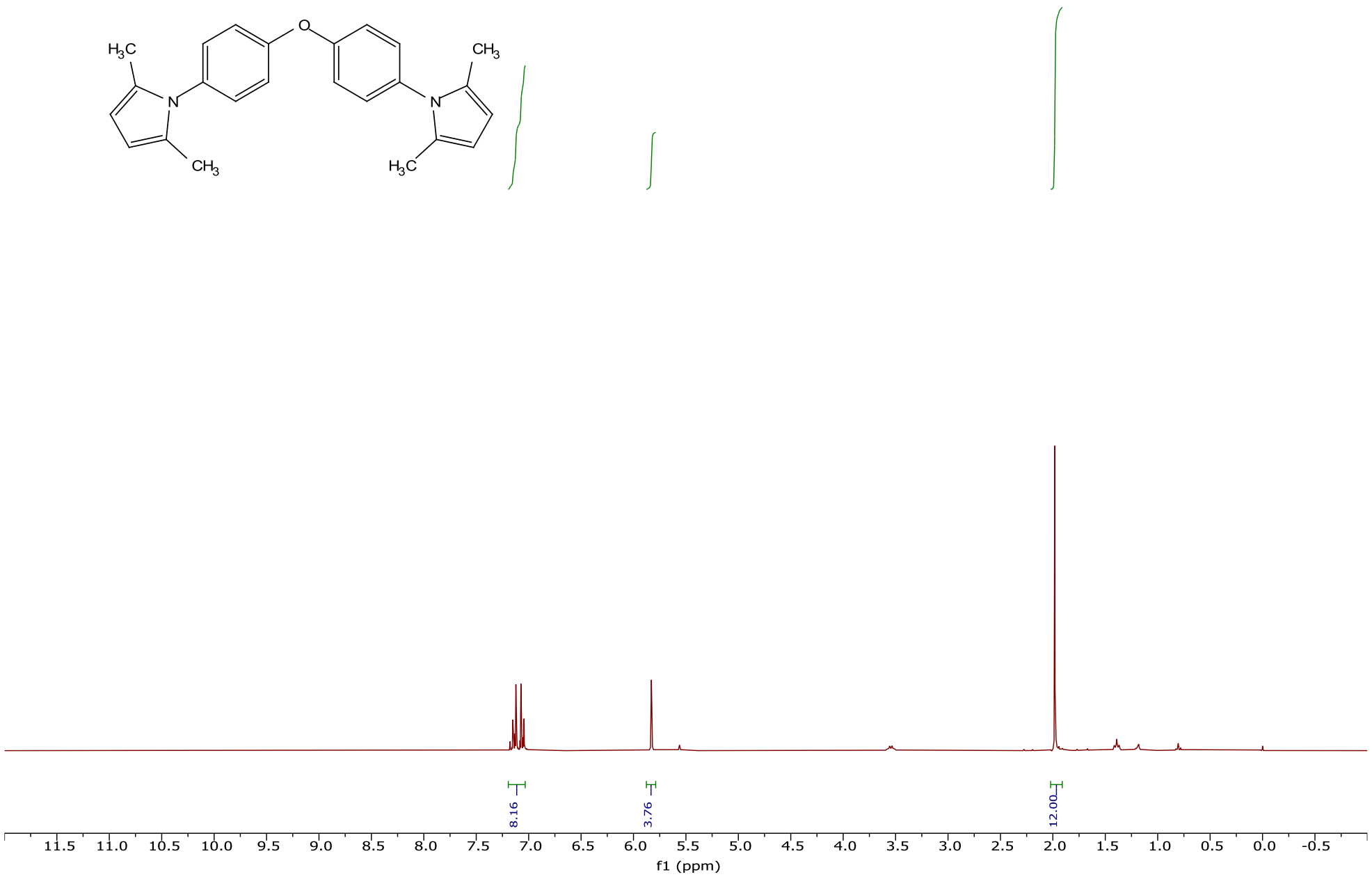

230503.344.11.fid
Haifeng Qi, HQ-742-1
Au13C CDCl3 {C:\Bruker\TopSpin3.6.2} 2305 44

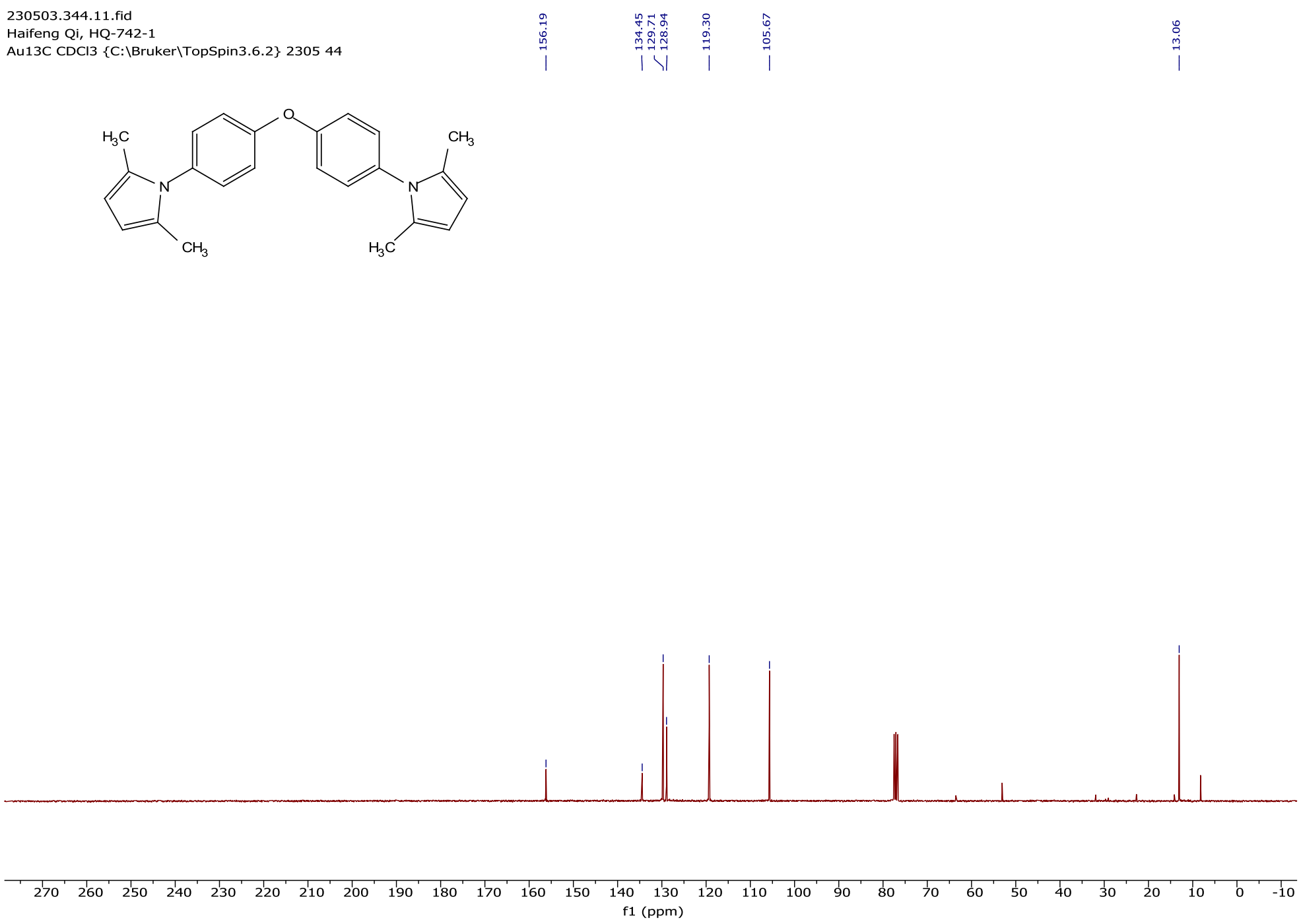

Supplementary Figure 45. ^{1}H and ^{13}C NMR spectra of product **29c**.

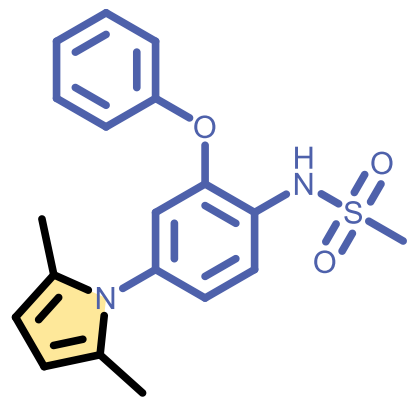

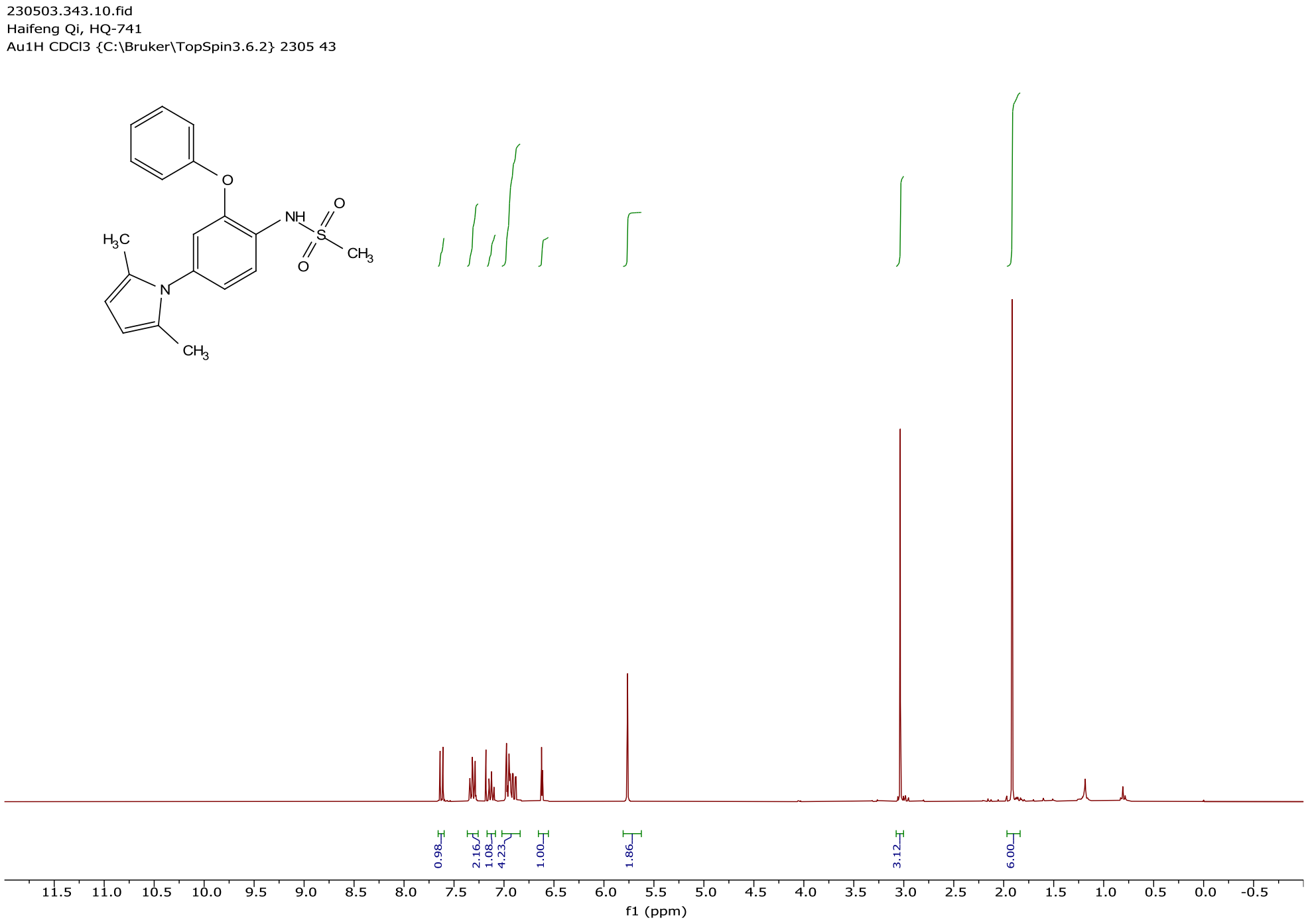

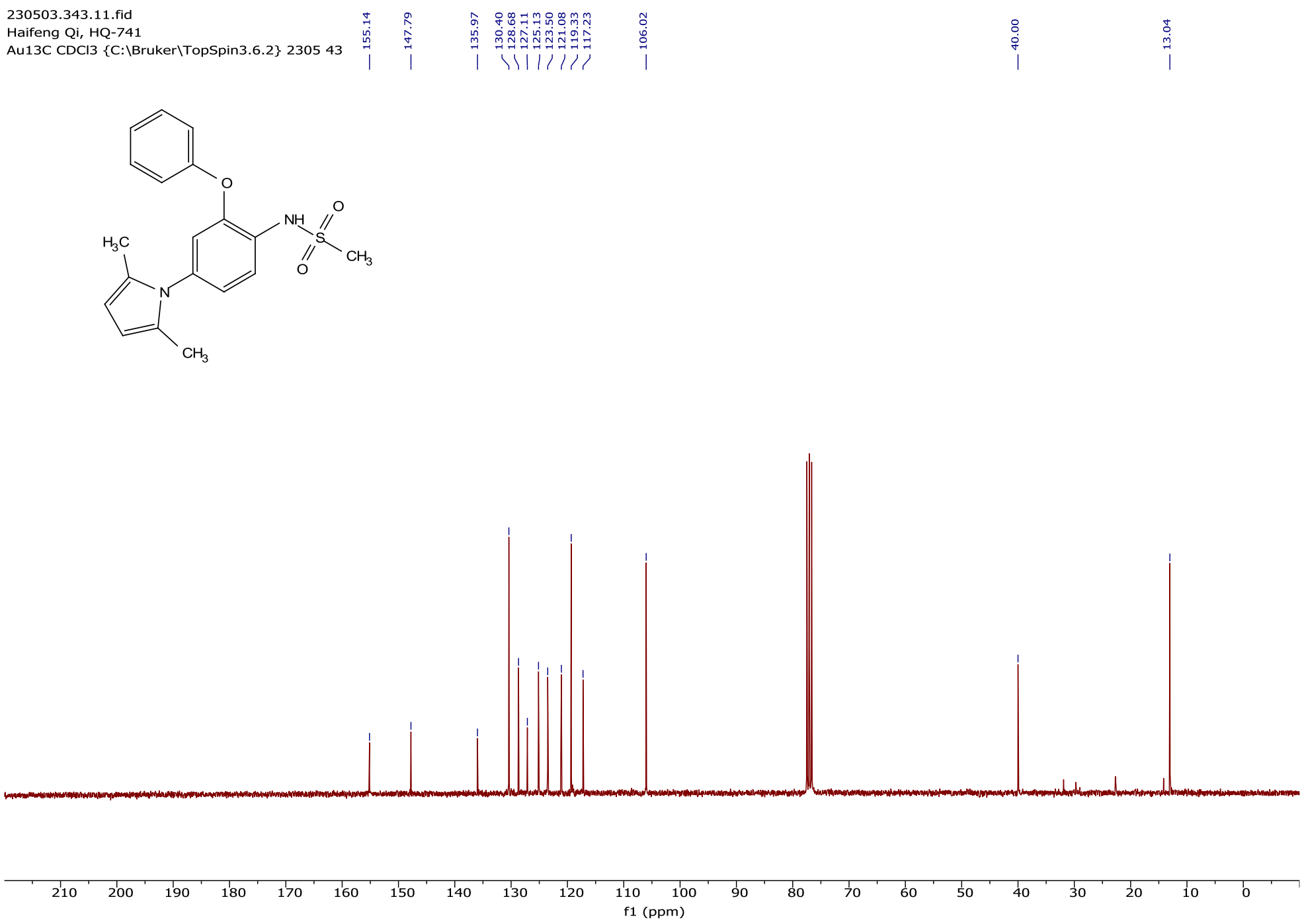

Supplementary Figure 46. ^{1}H and ^{13}C NMR spectra of product **31c**.

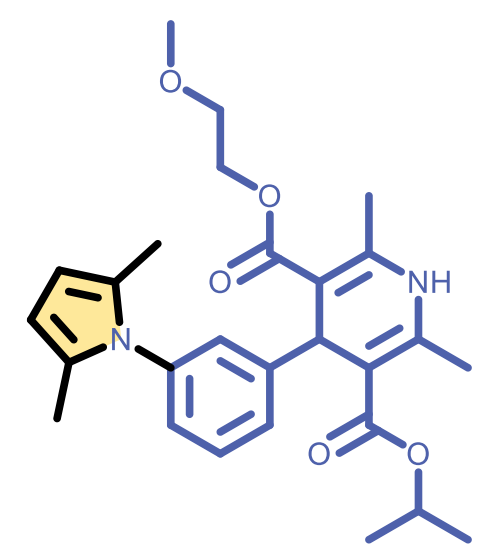

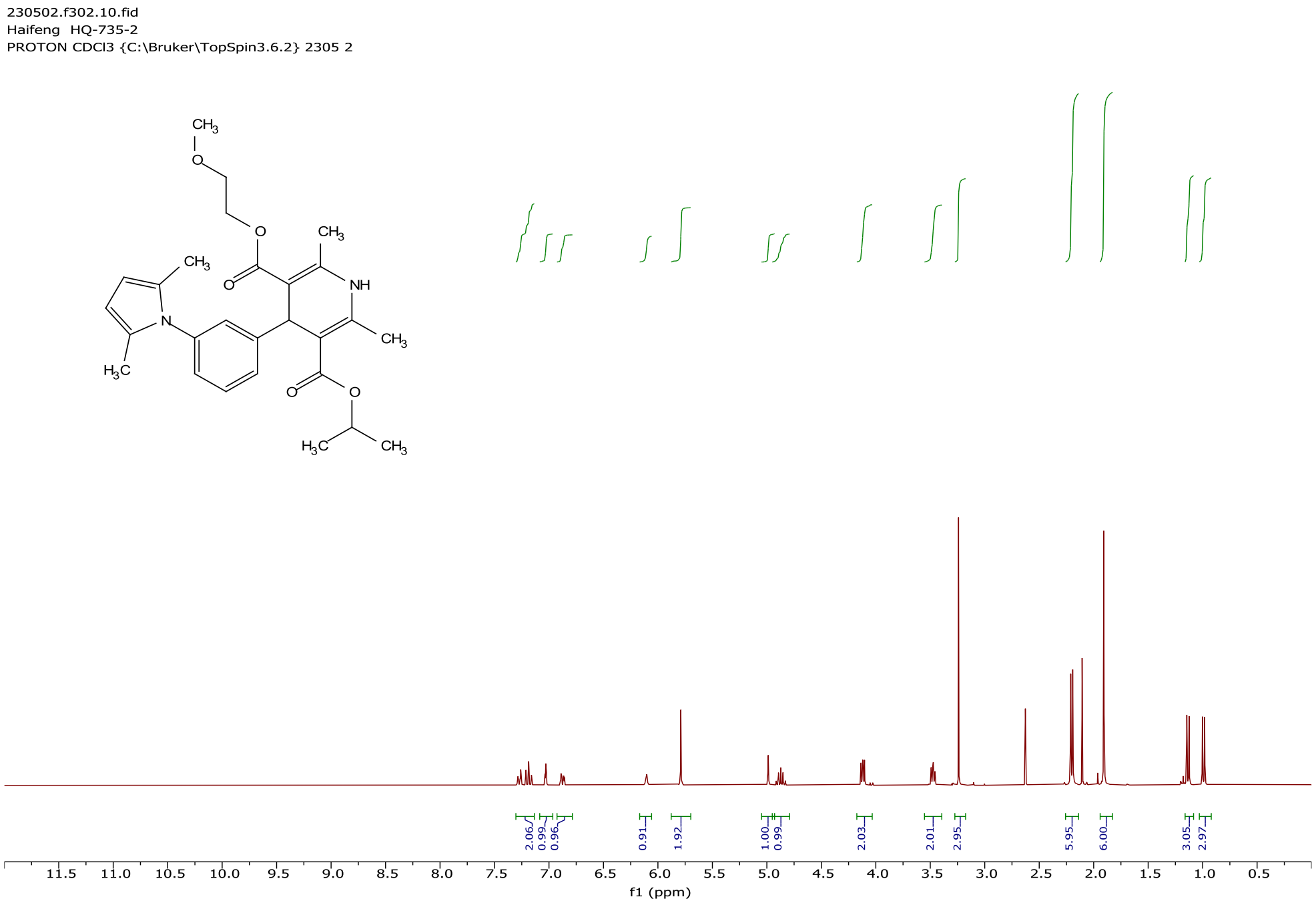

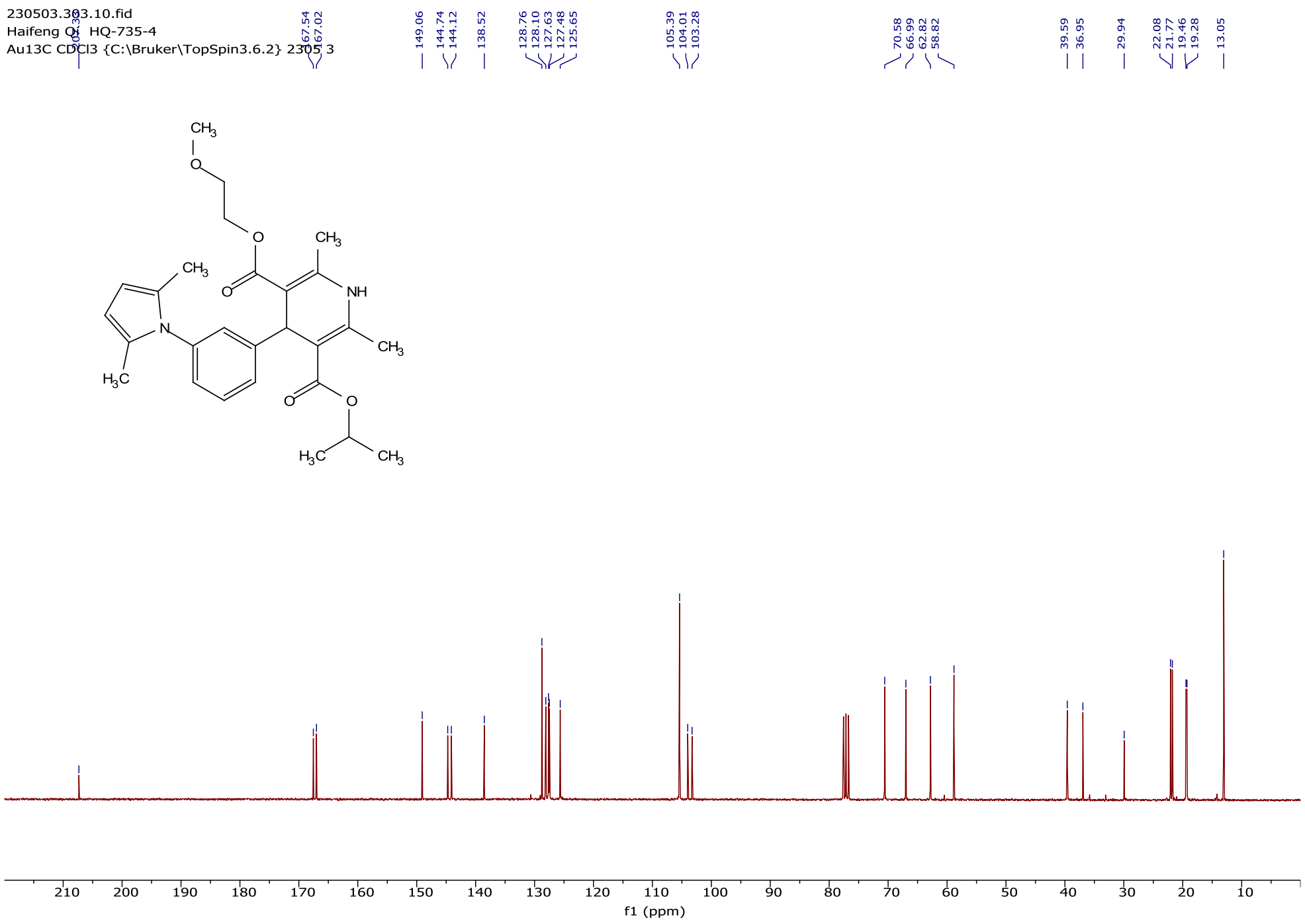

Supplementary Figure 47. ^{1}H and ^{13}C NMR spectra of product **32c**.

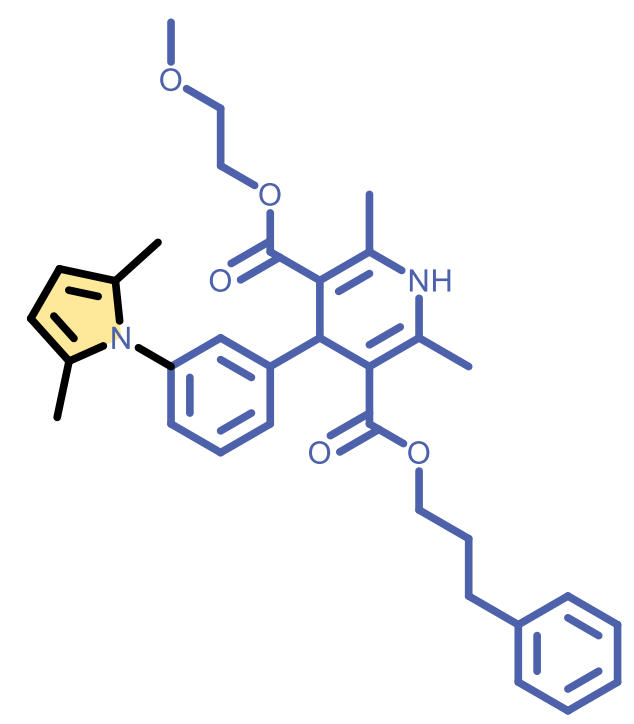

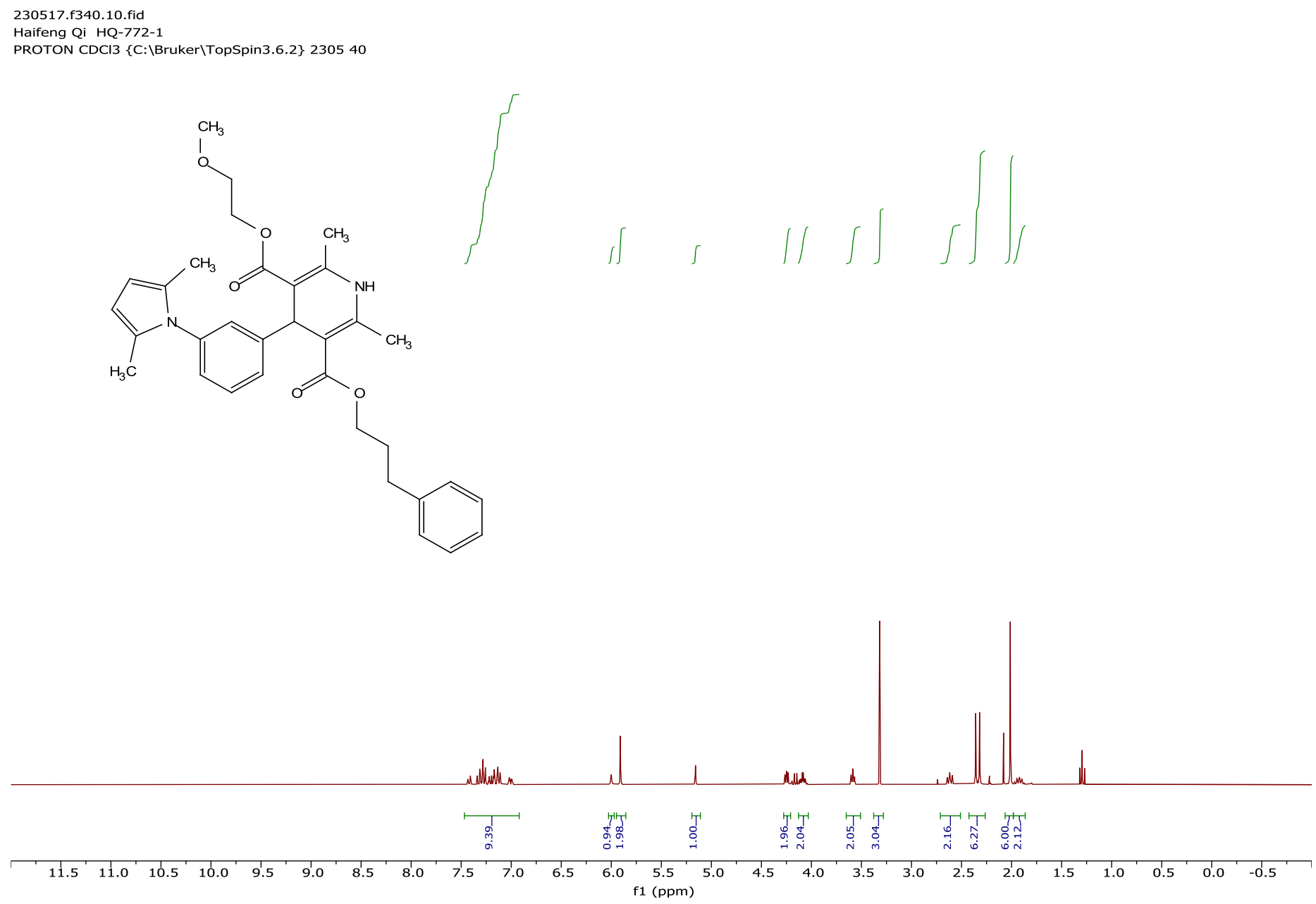

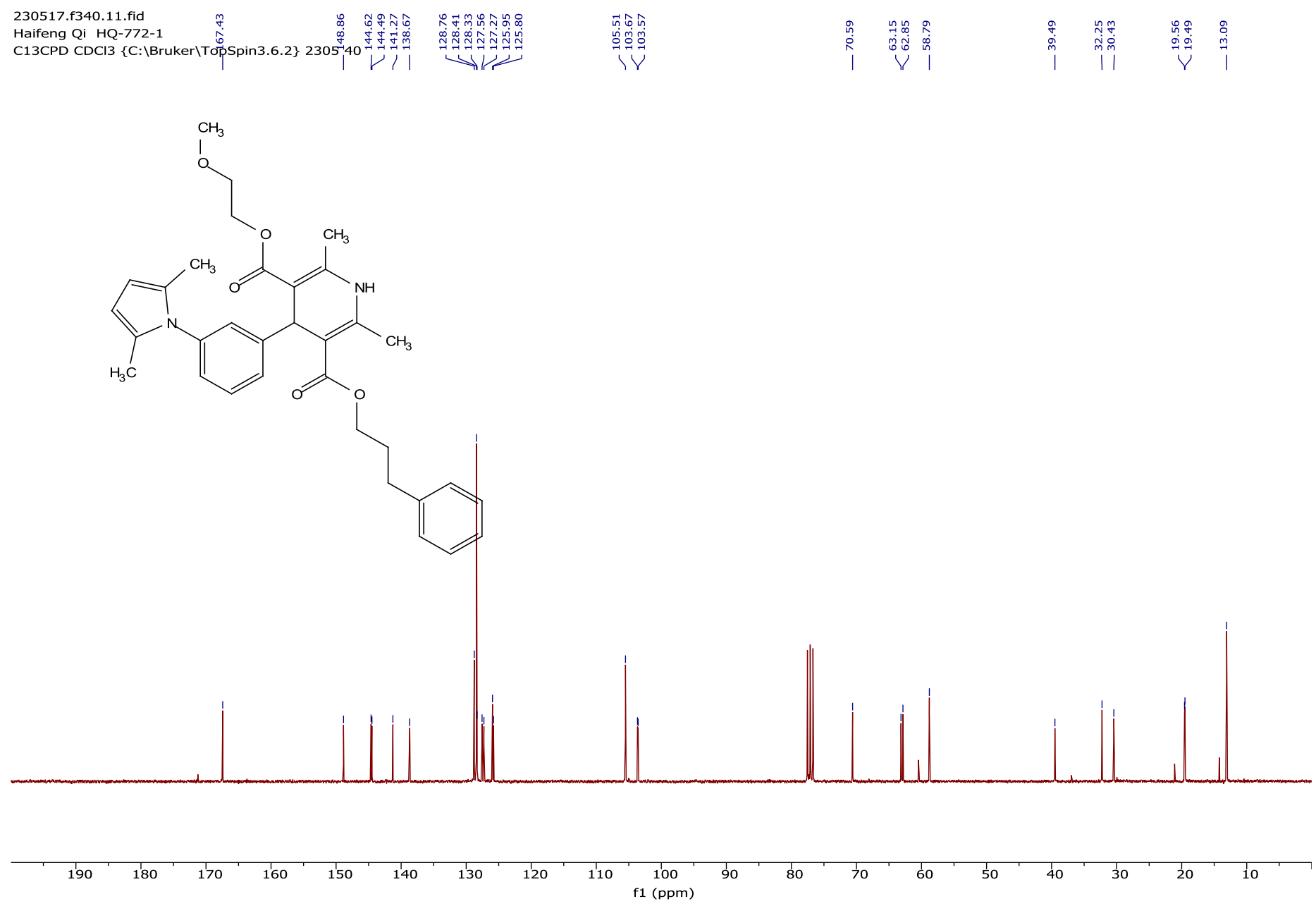

Supplementary Figure 48. ^{1}H and ^{13}C NMR spectra of product **33c**.
